# Microanatomical features of bovids long bones: What are the effects of mass and habitat?

**DOI:** 10.1111/joa.70140

**Published:** 2026-04-23

**Authors:** Morgan Proust, Cyril Etienne, Alexandra Houssaye

**Affiliations:** ^1^ UMR 7179, Mécanismes Adaptatifs et Evolution, Muséum National d'Histoire Naturelle Centre National de la Recherche Scientifique Paris France

**Keywords:** body mass, Bovidae, functional morphology, habitat, long bones, microanatomy

## Abstract

Bovids are a valuable group for studying limb long bone adaptations due to differences in size and the environment that the nearly 280 species inhabit. Their analysis can thus shed light on the constraints that occur during locomotion, which affect muscles and both the outer and inner bone structure. Nevertheless, no study has directly looked at the bone inner structure within bovids. This study is the first to analyse the inner structure of stylopod and zeugopod bones within bovids of different masses and habitats using both qualitative and quantitative approaches. Both thickness maps and virtual sections show remarkable differences in the heaviest individuals, which display a thicker compact bone layer, mostly at flexor muscle insertions associated with highly anisotropic trabeculae expending into the metaphysis. These qualitative results are supported by quantitative analyses that highlight a significant link to mass. By allowing greater dissipation of forces, these adaptations increase bone resistance to the associated higher compressive load and thus contact and ground reaction forces. As anticipated, the stabilising effect related to mass is evident in the forelimb and seems higher than that in the hindlimb. The influence of habitat is more evident in the hindlimb, but these observed variations are more subtle than those previously observed in the forelimb. Species that live in highly covered or mountainous areas, where a greater manoeuvrability is required, tend to display a globally higher bone volume fraction associated with the insertion of muscles increasing limb stability (e.g., *adductores*). While the gross shape of these bones shows a greater similarity between heavy‐bodied and open‐habitat bovids, the microanatomical features reveal a stronger similarity between heavy‐bodied bovids and those from covered and mountainous habitats than with open‐habitat species.

## Graphical abstract

This study provides the first qualitative and quantitative exploration of inter‐bones and interspecific variations in the microanatomy of long bones in bovids in response to significant changes in body mass and habitat.

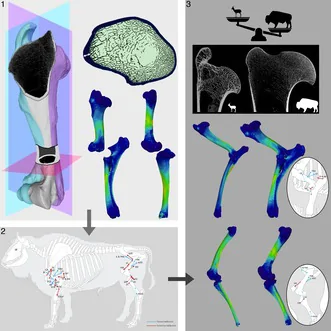

## 1 INTRODUCTION

Bovids show great variations in terms of size (from about 10 cm in *Madoqua kirkii* up to above 2 m in shoulder height in *Bos gaurus*), mass (from 4 kg to about a ton), morphology (gracile to robust), and habitat (e.g., open plains, forests, mountains) (Castelló, 2016; Scott & Barr, 2014). As a result of this diversity, various functional constraints can act on the musculoskeletal system, leading to variation, for instance, in the insertion of muscles (Grand, 1997; Maas et al., 2004; Stein & Casinos, 1997) and/or in bone morphology (Etienne et al., 2021; Mallet et al., 2021; Meloro et al., 2013; Pintore et al., 2021). This is particularly true for limb long bones, whose morphology can exhibit adaptive features related to body support and locomotion (Barone, 2017a; Biewener & Patek, 2018; Hall, 2008). These features can nevertheless also be the result of phylogenetic and structural factors (Gould, 2002; Seilacher, 1991).

Such adaptive features can also concern bone inner structure, which bears a strong functional signal, since it adapts to biomechanical constraints (Lambers et al., 2011; Nikander et al., 2010; Ruff & Hayes, 1983; Turner, 1998), maximising the resistance to loads by directing and dissipating the stress induced into the bone (Biewener & Patek, 2018; Frost, 1994; Lee & Taylor, 2003; Ruff et al., 2006; Wolff, 1986). Bone microstructure, therefore, reflects the various biomechanical constraints related to, for example, mass (Davies & Stock, 2014; Houssaye et al., 2018, 2022), habitat (Germain & Laurin, 2005; Nakajima et al., 2014; Ruimerman et al., 2005), and locomotion mode (Bishop et al., 2018; Ryan & Ketcham, 2002; Shaw & Stock, 2011). These changes can affect cortical thickness in various regions, such as underneath muscular insertion areas (Bader et al., 2022) and aspects of the trabecular network, like trabecular density and orientation (Gônet et al., 2023; Houssaye et al., 2022).

Numerous studies in bovids have demonstrated skeletal morphological adaptations to mass and habitat for limb long bones (Alexander, 1977; Etienne et al., 2021; Gregory, 1912; Kappelman, 1988; Moyà‐Solà et al., 2022; Scott, 1985). However, previous microanatomical studies have only integrated a few 2D sections, and none have focused on the specialisation of bone inner structure within bovid long bones (Canoville & Laurin, 2010; Houssaye, Sander, & Klein, 2016; Houssaye, Waskow, et al., 2016; Quemeneur et al., 2013). To highlight these potential adaptive features, this study proposes to qualitatively and quantitatively analyse the microanatomical variations present in bovid long bones based on computed tomography data of complete stylopod and zeugopod bones.

The most massive bovids are expected to display significant higher bone proportion (e.g., higher bone global compactness and trabecular density) to effectively dissipate the stress induced by their high body weight in accordance with Houssaye, Sander, and Klein (2016) and Houssaye, Waskow, et al. (2016), especially for the forelimb long bones due to their greater role in body weight support in most mammalian quadrupeds (Biewener & Patek, 2018; Schmitt, 1998). Since mass already appears to be the main factor driving external shape variation in the bovid long bones (Etienne et al., 2021), we expect to also observe significant microanatomical differences between the heaviest and lightest individuals, with a relatively thicker cortex and denser trabecular network when mass increases. An influence of the habitat is also expected with variation depending on the bone, in line with previous morphological investigations (Etienne et al., 2021), especially for the hindlimb, on which muscles with a primary propulsive role insert (Barone, 2017a; Hanot et al., 2017; Hildebrand, 1998; Lessertisseur & Saban, 1967). Species inhabiting covered or mountainous environments have enlarged insertion sites for the flexor muscles, allowing them to keep their limbs closer to their centre of mass. We therefore expect a thicker compact bone layer and a higher overall bone density, primarily in some regions of the diaphysis but also in the epiphysis, in order to better dissipate the higher induced forces associated with much more non‐linear and steeper movements than in open environments. A thicker layer of compact bone is anticipated at the insertion sites of extensor muscles in open habitats, likely to help meet the increased mechanical demands associated with an extended galloping stride. In addition, convergences might be expected between heavier bovids and species living in open habitats. Indeed, convergence linked with the respective need to move a greater mass and the higher forces required to increase speed in open environments has already been highlighted in the morphology of these bones (Etienne et al., 2021).

Overall, we aim to enhance our understanding of skeletal adaptation to functional constraints in bovids by analysing the microanatomical variation observed in their long bones with distinct masses and habitats. This study provides a unique opportunity to document intraindividual and interspecific variations, explore the major functional adaptive features, and identify bones more prone to microanatomical variation.

## 2 MATERIALS AND METHODS

### 2.1 Material

**FIGURE 1:**
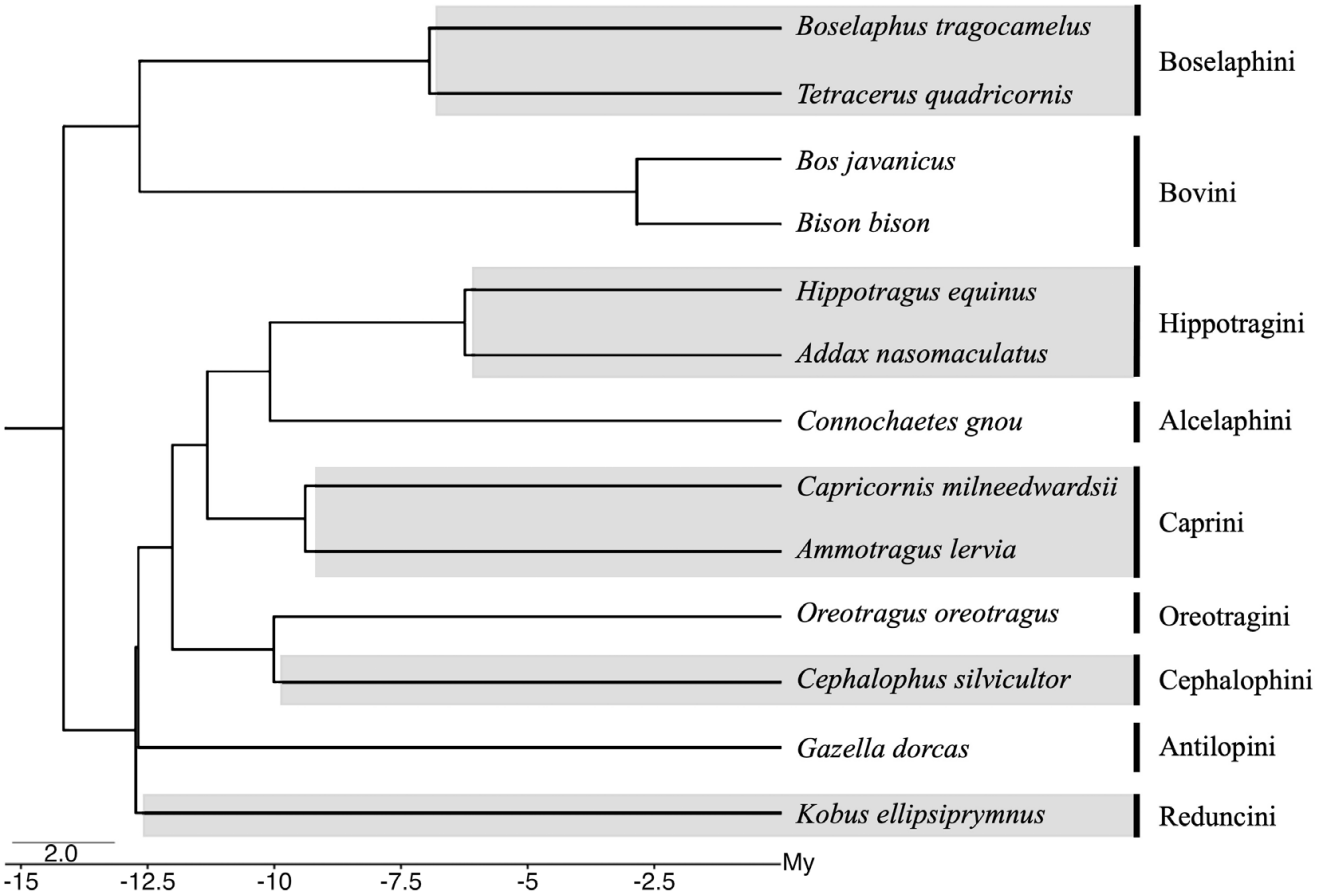
Simplified phylogeny of the Bovidae including the studied taxa, modified from Upham et al. (2019). Consensus tree of 100 trees under a majority‐rule consensus of 80% generated in Mesquite (Maddison & Maddison, 2023).

The sample (Table 1) consists of 13 species from 9 of the 12 tribes (Bibi, 2013) of extant bovids. The material used in this study consists of 52 long bones (13 humeri, femora, radii‐ulnae, and tibiae) from the comparative anatomy collections of the Museum National d'Histoire Naturelle (Paris, France). This sample covers most of the diversity (morphological, mass, and environmental) of bovids within the different tribes (Figure 1; Table S1.1). Based on the advanced degree of fusion observed (Table S1.7; Supporting Information: Data 4) between the epiphyses and diaphysis (i.e., presence/absence of epiphyseal line), the individuals are all considered adults (when absent) or sub‐adults (when present) (Duffield, 1973; Etienne et al., 2021). Mass estimates (Table 1) of the different species sampled follow those established by Castelló (2016) and range from 13.5 kg (*O. oreotragus*) to 680 kg (*B. bison*). As body size sexual dimorphism is pretty common in bovids and can have a significant effect on mass (Castelló, 2016), and as sex of the individuals is rarely recorded in the collections, we therefore averaged the smallest and largest masses for each species. As the distinction between habitats is always ambiguous, due to their continuous nature, we have separated the different environments into four main groups (Table 1), as in several studies (Castelló, 2016; Etienne et al., 2021; Scott & Barr, 2014; Takeda et al., 2023): (1) open (OE; no or low relief area with large spaces displaying a low vegetation density), (2) light cover (LCE; high vegetation density), (3) dense cover (DCE; extremely high vegetation up to forest, since our sample size did not allow the separation between these two highly covered environments), and (4) mountainous (ME; positive relief with abrupt slopes) environments.

**TABLE 1:** List of the bovid specimens used in this study with associated sex and origin when known, the habitat and approximate mass assigned based on Castelló (2016), and the associated CT‐scanned resolution.

Species	Abb.	Specimen collection number	Age	Sex	Origin	Habitat	Mass (kg)	Voxel size (μm)
*Bison bison*	Bb	MNHN‐ZM‐AC‐1885‐339	S	F	Historical zoo	Open	679 (360–998)	138
*Bos javanicus*	Bj	MNHN‐ZM‐AC‐1944‐101	A	M	Vincennes zoo	Heavy cover	600 (400–800)	139
*Tetracerus quadricornis*	Tq	MNHN‐ZM‐MO‐1994‐2317	A	F	Historical zoo	Heavy cover	20 (15–25)	70
*Boselaphus tragocamelus*	Bt	MNHN‐ZM‐AC‐1864‐103	S	F	Unknown	Light cover	205 (200–290)	133
*Addax nasomaculatus*	An	MNHN‐ZM‐AC‐1970‐277	A	F	Historical zoo	Open	92.5 (60–125)	95
*Hippotragus equinus*	He	MNHN‐ZM‐AC‐1969‐167	A	F	Wild	Light cover	275.5 (215–300)	124
*Connochaetes gnou*	Cg	MNHN‐ZM‐2013‐26	A	F	Unknown	Open	145 (110–180)	98
*Ammotragus lervia*	Al	MNHN‐ZM‐2010‐643	A	M	Vincennes zoo	Mountain	87.5 (30–145)	81
*Capricornis milneedwardsii*	Cm	MNHN‐ZM‐AC‐1874‐283	A	M	Wild	Mountain	112.5 (85–140)	112
*Kobus ellipsiprymnus*	Ke	MNHN‐ZM‐AC‐1935‐637	S	F	Vincennes zoo	Heavy cover	217.5 (160–275)	128
*Gazella dorcas*	Gd	MNHN‐ZM‐MO‐1974‐113	S	M	Wild	Open	19 (15–23)	61
*Oreotragus oreotragus*	Oo	SSN	A	NA	Unknown	Mountain	13.5 (9–18)	70
*Cephalophus silvicultor*	Cs	MNHN‐ZM‐MO‐1981‐1023	A	M	Vincennes zoo	Heavy cover	62.5 (45–80)	80

Abbreviations: A, adult; S, subadult.

### 2.2 Methods

#### 2.2.1 Imaging

Bones were scanned using a high‐resolution X‐ray tomograph (GEphoenix∣X‐ray v∣tome∣xs 240) at the AST‐RX platform (OMSI, UMS 2700) at the MNHN. Reconstructions were made using DATOX/RES, Phoenix datos|x. Voxel sizes ranged between 60 and 140 μm, depending on specimen size (Table 1).

#### 2.2.2 Image analysis

To perform quantitative analyses of the bone inner structure, bone tissue segmentation was performed, partially automatically. To that end, we carefully selected between 10 and 15 reference images with Image J/Fiji (Schindelin et al., 2012) and then segmented them into binary images (black and white levels instead of grey levels) using Avizo 2019.1 (Burlington, MA). Based on grey level differences, these images were selected to represent the most challenging cases for differentiating bone, marrow, and fixation material. Using these images as a reference, the segmentation process was automated using the Weka plugin (Arganda‐Carreras et al., 2017) of the ImageJ software. Two systematic post‐treatment verifications were then performed: First, a comparison was made between images randomly selected within the original sections and the segmented ones, ensuring the quality of the segmentation. Second, the raw data stack was compared with the segmented one on VG Studio MAX 2.2 (Volume Graphics Inc., Heidelberg, Germany) to ensure the quality and coherence of the whole segmented stack. The ‘refine’ function of VG Studio was used during the second post‐treatment, in order to better fit in 3D the segmented bone tissue to the local grayscale gradient. To analyse different compactness parameters inside trabecular bone alone and carry out compact bone thickness cartographies, we used Avizo 2019.1 and manually separated the compact (cortical in the diaphysis and endochondral in the epiphyses) layer and trabecular bone following Houssaye et al. (2021).

#### 2.2.3 Qualitative analyses

##### Virtual sections

**FIGURE 2:**
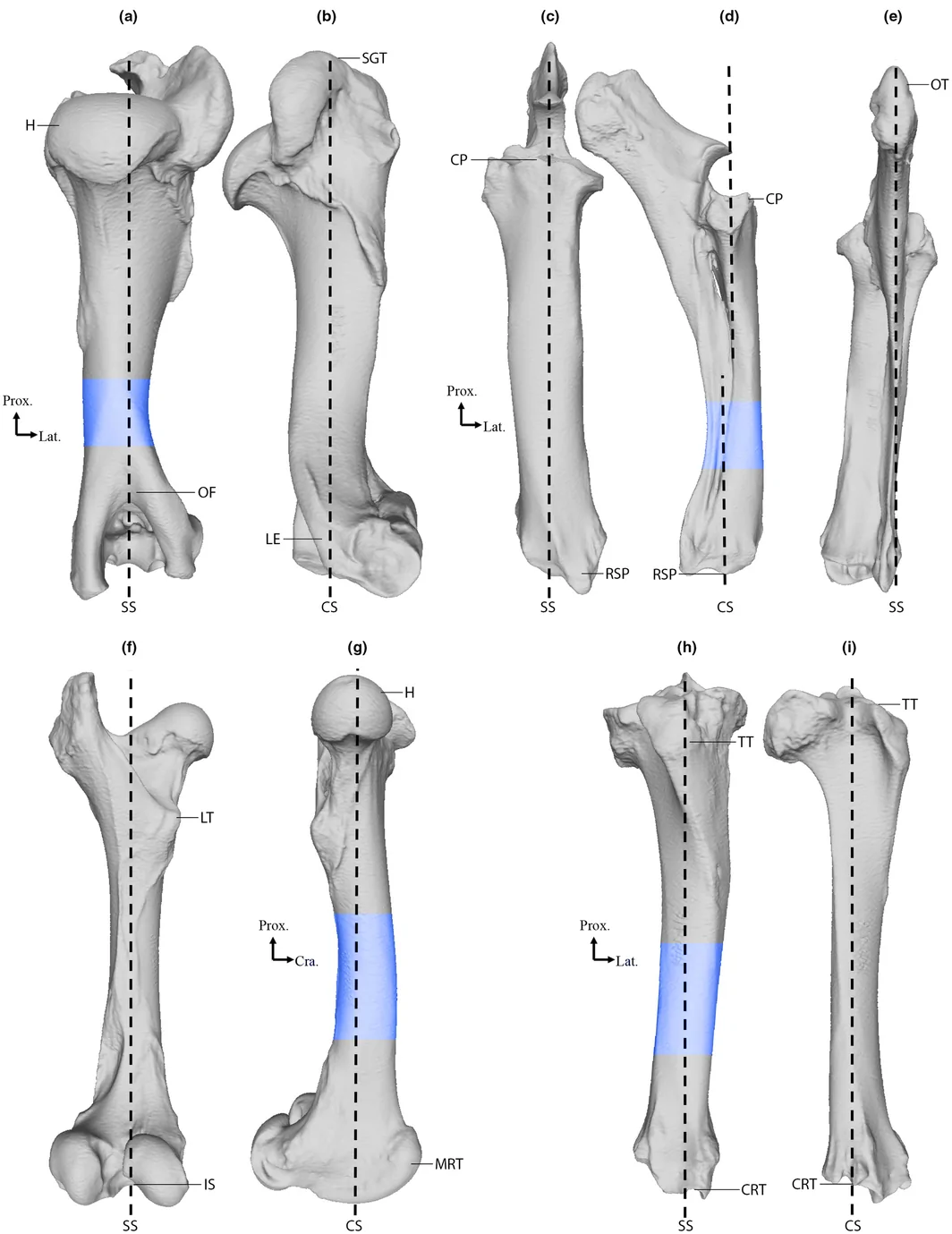
Planes used for the virtual sections. Humerus caudal (a) and lateral (b) views; Radius, frontal (c) and medial views (d); Ulna, caudal view of the olecranon (e); Femur, medial (f) and caudal views (g); Tibia, frontal (h) and lateral views (i), of Bison bison MNHN‐ZM‐AC‐1902‐316 illustrating the planes of the virtual sections (CS, coronal section; SS, sagittal section). Anatomical abbreviations: CP, coronoid process; CRT, central ridge of the trochlea; H, head; IS, intercondylar space; LE, lateral epicondyle; LT, lesser tubercle; MRT, medial ridge of the trochlea; OF, olecranon fossa; OT, olecranal tuber; RSP, radial styloid process; SGT, summit of the great tubercle; TT, tibial tuberosity. The blue areas indicate the regions exhibiting the least apparent degree of variation between the different individuals.

In order to describe the microanatomical structure along the bones, VG Studio MAX 2.2 was used to create virtual sections of the raw data. Two longitudinal sections were taken, one coronal and one sagittal, with the bones oriented based on morphological criteria from Barone (2017a), to visualize microstructure variations along the bone, following Houssaye, Fernandez, and Billet (2016) (Figure 2). To obtain the sagittal section, the bone was oriented caudally for the humerus (Figure 2a), ulna (Figure 2e), and femur (Figure 2f), and cranially for the radius (Figure 2c) and tibia (Figure 2h). The sagittal section was defined as the plane crossing the diaphysis at its centre, when the bone was viewed cranially (radius and tibia; Figure 2c,h) or caudally (humerus, ulna, femur; Figure 2a,e,f). The coronal section was defined as the plane cutting the middle of the diaphysis, perpendicular to the sagittal plane (Figure 2b,d,g,i). Diaphyseal curvature of the radius prevented sections that included the entire diaphysis; two coronal sections were thus taken, one for the proximal epiphysis and one for the distal one (Figure 2d). Since proper virtual sections for the entire ulna could not be obtained due to the fusion with the radius, and its thinness and curvature, we made an additional sagittal section of the olecranon at its centre (Figure 2e). We also carried out transverse virtual sections for all bones at the growth centre (i.e., where the cortex is the thickest) following Houssaye et al. (2018).

##### Thickness maps

Thickness maps enable us to analyse the variations in the thickness of the outer layer of compact bone along the bones and thus highlight areas where it is relatively thickened. This can be particularly relevant for understanding functional adaptations when thickening can be linked to muscle insertion (Bader et al., 2022; Harbers et al., 2020). After separating the compact and trabecular territories, compact bone thickness cartographies were made. For these, small isolated cavities and irregularities in compact bone were removed using, with caution, the Avizo ‘smooth labels’ function. The distance between the outer and inner limits of compact bone was calculated using the ‘surface distance’ function. A colour scale, specific to each bone, has been established to visualise compact bone thickness differences across the bone and subjectively classify them (Table S1.6; From blue, which corresponds to the thinnest value of 1, to red, which corresponds to the thickest value of 6).

#### 2.2.4 Quantitative analyses

##### Parameter acquisition

Quantitative analyses are based on a combination of different standardized parameters obtained with Avizo's ‘material statistics’ function or via the compact bone thickness maps (Tables S1.2–S1.4), following Houssaye et al. (2022): Whole bone volume (WBV; mm^3^); bone compactness (C; as bone tissue volume [BTV] over WBV); relative fraction of trabecular bone (%trab, as the trabecular bone volume [TBV] over BTV); trabecular compactness (Tc, as TBV over the trabecular space volume [TSV]); mean compact thickness (MeanT; in mm); maximal compact thickness values (MaxT; in mm); relative mean compact thickness (RmeanT); relative maximal compact thickness (RmaxT).

Because of the variation of size and shape in each species, we identified the portion of the diaphysis that was changing the least for each bone (Figure 2, blue shaded area). We then virtually extracted these portions using Geomagic (Hexagon AB, USA) in order to keep only this area. The relative mean compact thickness (RmeanT) and maximal compact thickness (RmaxT) were obtained by approximating these portions to cylinders, dividing the absolute values (Rmean and Rmax) by the average radius (*r*): *r* = (Vol/*h* × *π*)^1/2^, with Vol, as the cylinder WBV, h as the length of the cylinder. All statistical analyses and graphs below were coded (Supporting Information: Data 7) and performed with R (R Core Team, 2024.04.2) and RStudio (Allaire, 2011).

##### Statistical analyses

The whole bone volume (WBV) was used as a size estimate following Houssaye et al. (2021) and a least square regression (lm function in R) was performed between the cube root of WBV, the dependent variable, and the species mean mass (Table 1; Table S1.4), the independent variable, to check how this size estimate was related to species mass.

We created violin plots (geom_violin function in R) to illustrate variation in the different microanatomical parameters between habitats. Due to the limited size of the sample, the impact of size on most of the microanatomical parameters, and the absence of a robust non‐parametric test that accounts for a covariate, we chose to describe microanatomical parameters exclusively in a qualitative manner based on the violin plots (Figures S3.1–S3.3). Nevertheless, a Friedman test (friedman.test function) was performed to compare these parameters between the different bones, except for one specimen (*C. gnou*) due to the absence of fusion in its radius and ulna. We then performed a Nemenyi post‐hoc test (frdAllPairsNemenyiTest function) to test which bone(s) differ from the others.

We performed, in addition, a principal component analysis (PCA) including all microanatomical quantitative parameters, for each skeletal element, to describe the variation between the specimens (Supporting Information: Data 6; Figure S1). To assess the impact of mass on each axis, we then performed a linear regression between the WBV and PC axes.

## 3 RESULTS

Here, we first qualitatively investigate the microanatomical diversity in the various long bones through the description of the virtual sections and compact bone thickness maps. We then present the results from the quantitative analyses testing for the influence of size and habitat on the variability of the studied microanatomical parameters.

### 3.1 Qualitative description

#### 3.1.1 Virtual thin sections

##### Generality

**FIGURE 3:**
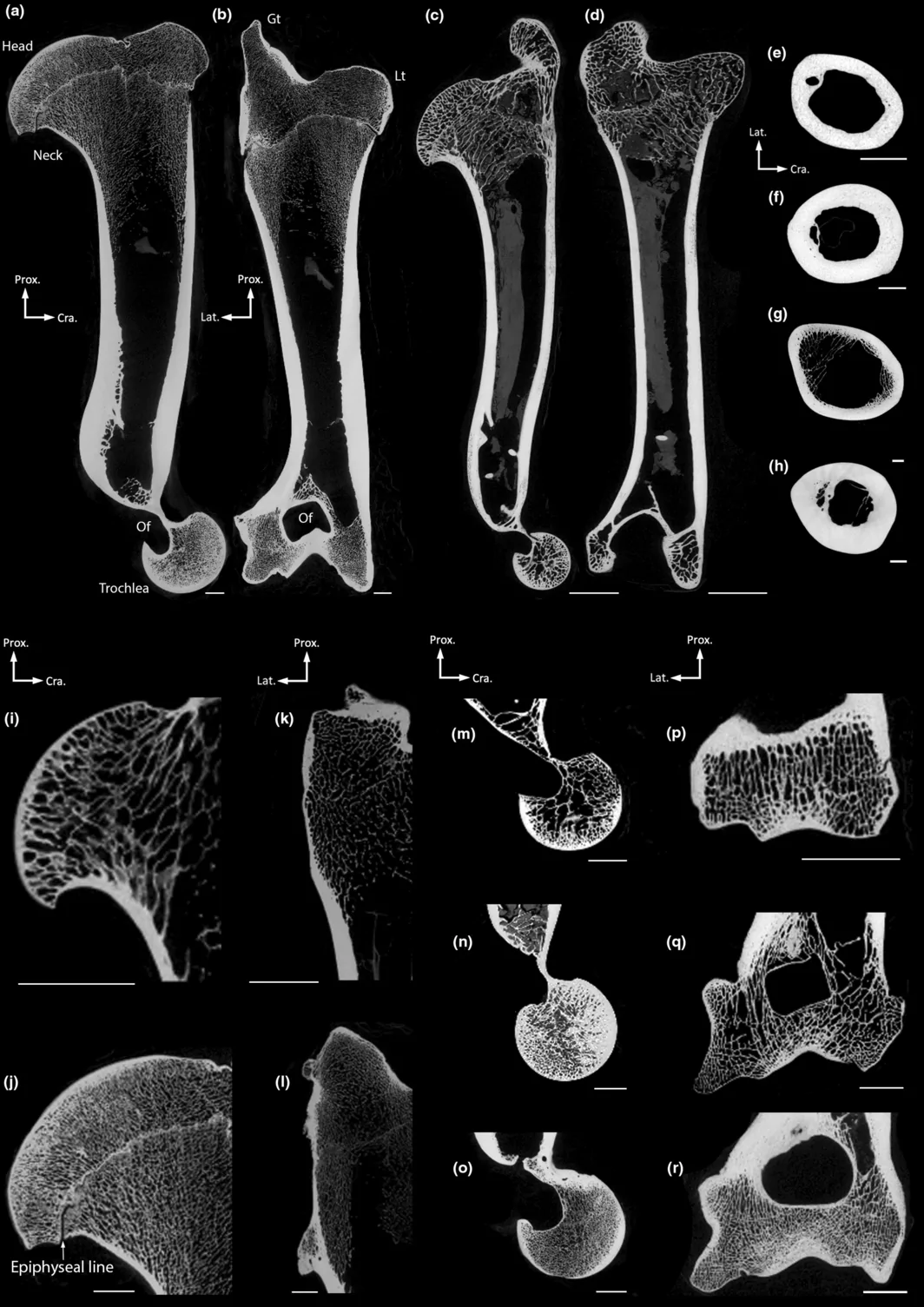
Sample of the virtual sagittal (SS), coronal (CS), and transverse (TS) sections made on the humeri displaying the most striking mass or habitat adaptations. The whole bone: (a, b) B. javanicus SS (a) and CS (b); (c, d) G. dorcas SS (c) and CS (d). Nutrient canal (TS): O. oreotragus (e); C. silvicultor (f); B. bison (g); B. javanicus (h). Zoom on the humeral head (SS): O. oreotragus (i); B. javanicus (j). Zoom on the greater tubercle (CS): T. quadricornis (k); B. tragocamelus (l). Zoom on the trochlea (SS): A. nasomaculatus (m); C. milneedwardsii (n); B. tragocamelus (o). Zoom on the distal epiphysis (CS): O. oreotragus (p); A. nasomaculatus (q); C. gnou (r). Scale bar: 10 mm. Gt, greater trochanter; Lt, lesser trochanter; Of, olecranon fossa.

The nutrient canal exits from one preferential side for each bone, except for the femur, for which it seems to exit randomly (Table S1.5; Supporting Information: Data 4). The position of the nutrient foramen along the bone varies depending on the animal's mass, being mostly more distal in heavier species (3/4 down the diaphysis) and more proximal for lighter species (mid‐diaphysis), except for the radius, for which the opposite pattern occurs (Table S1.5; Supporting Information: Data 4). Trabecular bone crossing the medullary space rarely occurs in transverse sections, especially in heavy species. This is true for all bones, except for the femur, in which a few trabeculae can especially be seen (Figure 5f). For heavier bovids, the layer of compact bone is globally thicker (Figure 3a,b). Trabecular bone organisation becomes more isotropic near the articular surfaces of each bone (Figure 5i–k).

##### Forelimb

###### Humerus

Most humerus transverse sections are oval, except for *Bos javanicus*, *Addax nasomaculatus*, *Cephalophus silvicultor*, and *Boselaphus tragocamelus*, which display rounder sections (Figure 3f,h). Bovids with the thickest compact bone are *B. tragocamelus*, *B. javanicus*, *Kobus ellipsiprymnus*, *Hippotragus equinus*, *Ammotragus lervia*, and *Connochaetes gnou*, which are the heaviest species in the sample and live mostly in DCE or ME. Thicker compact bone appears: In the proximal epiphysis, especially under the humeral neck for light species, excluding *C. silvicultor*, and for *H. equinus*; at the top of the humeral head for the heaviest species (Figure 3a,c,i,j); and underneath the greater tubercle for species mostly living in a DCE or ME (*B. javanicus*, *Tetracerus quadricornis*, *A. lervia*, *Capricornis milneedwardsii*, *C. silvicultor*, and *C. gnou*; Figure 3b,k,l). In the distal epiphysis, the compact bone is thicker proximal to the olecranon fossa for all species, excluding *Bison bison*, *A. nasomaculatus*, and *C. milneedwardsii* (Figure 3m,n), and at the trochlea for some of the heaviest species of the sample (*B. javanicus*, *B. tragocamelus*, *K. ellipsiprymnus*, *C. milneedwardsii*, *A. lervia*; Figure 3n,o). Trabecular bone under the greater and lesser tubercles appears predominantly proximo‐distally anisotropic, but more isotropic in the lightest species (Figure 3k) than in heavier ones (Figure 3l). In the distal epiphysis, a gradient of anisotropy can also be observed with the trabeculae oriented at first medio‐laterally, above the olecranon fossa, then shifting to nearly proximo‐distal at the olecranon fossa up to the distal articular surfaces, where trabecular organisation becomes more random. This shift is very clear for the heaviest species of the sample, but also for *Oreotragus oreotragus*, which is one of the lightest (Figure 3p–r). Bovids with greater apparent trabecular bone density in the proximal epiphyses are *B. javanicus*, *K. ellipsiprymnus*, *A. lervia*, *B. tragocamelus*, *H. equinus*, *C. silvicultor*, and *B. bison*, which are mostly the heaviest species of the sample; higher density in trabecular bone occurs in specific regions like the bicipital groove, the neck of the lesser tubercle, near the deltoidal tuberosity, the great round tuberosity (Figure 3b,l), and on the caudal part of the humeral head (Figure 3i,j). It is also seen in the trochlea in the heaviest individuals, and for species living in DCE or ME (Figure 3n,o).

###### Radius

**FIGURE 4:**
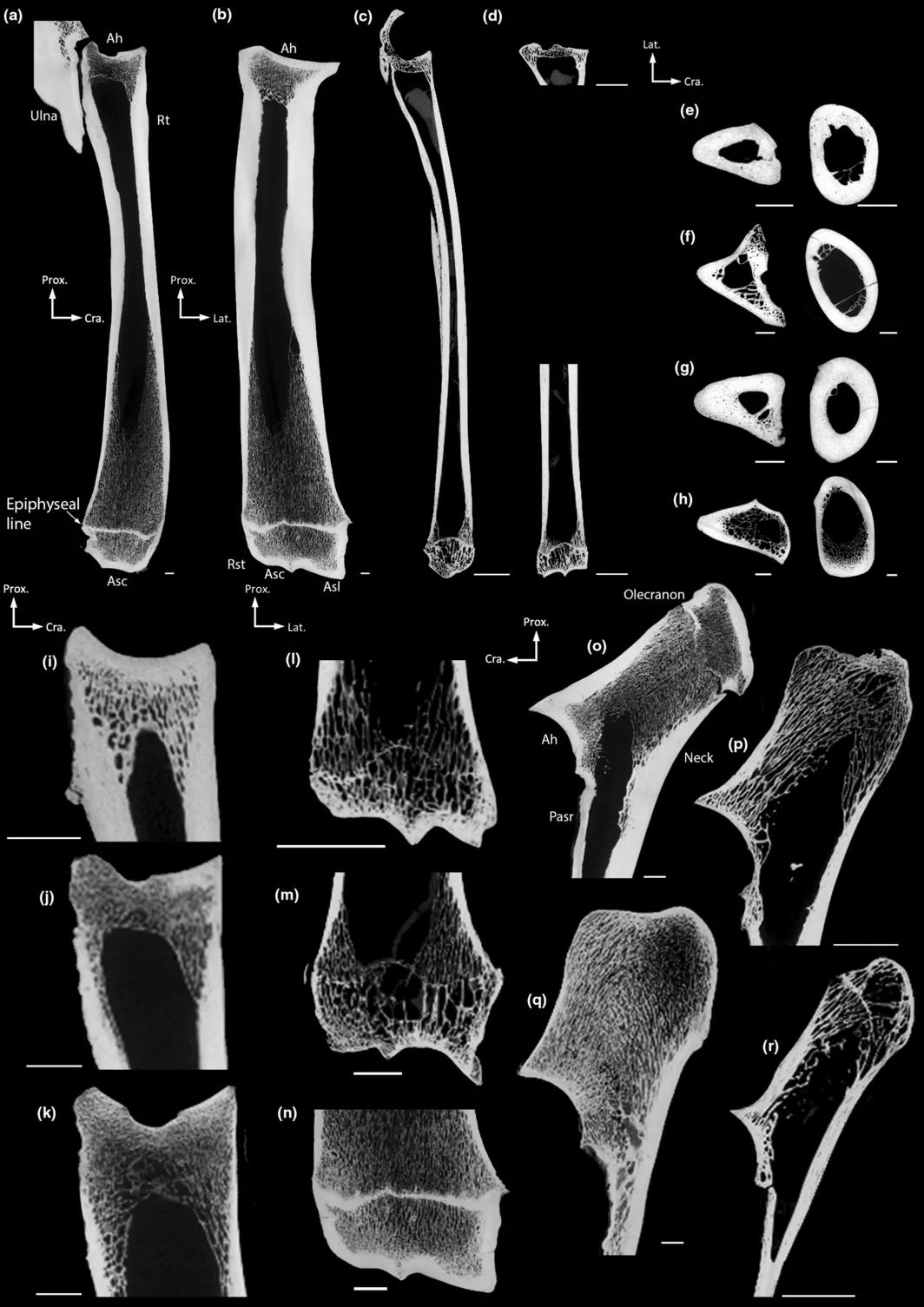
Sample of the virtual sagittal (SS), coronal (CS), and transverse (TS) sections made on the radius‐ulna displaying the most striking mass or habitats adaptations. The whole bone: (a, b) B. javanicus SS (a) and CS (b); (c, d) G. dorcas SS (c) and CS (d). Nutrient canal (TS): T. quadricornis (e); C. milneedwardsii (f); C. gnou (g); B. bison (h). Zoom on the articular surface with the humerus (CS): A. lervia (i); B. tragocamelus (j); B. bison (k). Zoom on the articular surface with the carpus (CS): O. oreotragus (l); A. nasomaculatus (m); B. javanicus (n). Zoom on the olecranon of the ulna (SS): B. javanicus (o); A. nasomaculatus (p); C. milneedwardsii (q); G. dorcas (r). Scale bar: 10 mm. Ah, articular surface for the humerus; Asc, articular surface for the scaphoid; Asl, articular surface for the semilunar; Pasr, proximal articular surface for the radius; Rst, radial styloid process; Rt, radial tuberosity.

All radius transverse sections are oval (Figure 4e–h), and the compact bone thickness is rather homogeneous, except for *B. javanicus*, *C. gnou*, *K. ellipsiprymnus*, *C. silvicultor*, and *B. tragocamelus*, for which compact bone is thicker on the medial side (Figure 4g). Bovids with the thickest compact bone in transverse section are *A. nasomaculatus*, then *B. javanicus*, *C. gnou*, and *K. ellipsiprymnus*, which are some of the heaviest bovids of our sample and live mostly in a DCE or ME. Thicker compact bone appears: at the articular surface with the humerus, on the cranial side, but it is less visible for OE and LCE species (Figure 4k) compared with the others (Figure 4i,j); and on the caudal side for species living in a DCE or ME (Figure 4i). In the distal epiphysis, compact bone is thicker at the articular surface of the scaphoid for most individuals (Figure 4l–n); it also occurs at the radial styloid process (Figure 4n) and at the articular surface with the semilunar (Figure 4l,n) for species living in a DCE or ME. On the caudal side, the trabeculae are slightly oriented proximo‐distally near the proximal interosseous space and then oriented cranio‐caudally next to the articular surface of the humerus (Figure 4j,k) in most individuals (Figure 4i); on the most medial and lateral parts of the proximal epiphysis, they are oriented proximo‐distally, while they radiate more medio‐laterally from the centre of the epiphysis to the proximal metaphysis (angle around 10°; Figure 4a,c). In the heaviest species, trabecular bone extends from the mid‐diaphysis to the distal epiphysis, with trabeculae oriented approximately proximo‐distally (first at a ~10° angle, from the medial to the lateral side, then nearly perfectly aligned). In the lightest species, trabecular bone follows the same pattern, but with a lower angle (angle around 20°; Figure 4l–n). The trabecular organisation tends to be more random at the end of the distal epiphysis (where the radius articulates with the carp) in all species. As for the humerus, the apparent trabecular density is the highest in the heaviest species, especially in the distal epiphysis, and maximal in *B. javanicus*.

###### Ulna

A medullary space is present in the olecranon for most of the sampled species, but it is reduced in species mostly living in a DCE (*B. bison*, *C. silvicultor*, *K. ellipsiprymnus*, *T. quadricornis*) and absent in *C. milneedwardsii* (Figure 4q). There is no medullary space in the ulnar diaphysis, except in the large *B. bison* and *K. ellipsiprymnus*, which present, respectively, a medullary space filled with trabeculae and an extremely reduced medullary space. Ulna transverse sections vary from oval to triangular (Figure 4e–h) without regard to mass, but occur with a thicker compact bone on the caudal side in heavier species (*C. gnou*, *B. bison*, *B. javanicus*, *H. equinus*, and *A. lervia*; Figure 4g,h). Bovids with the thickest compact bone in transverse section are *C. gnou*, then *A. lervia*, *B. javanicus*, and *B. bison*. Thicker compact bone appears: at the olecranon, on the cranial and caudal sides, mostly in heavy individuals living in a DCE or ME (Figure 4o); at the articular surface of the humerus, except in *C*. *milneedwardsii*, *G. dorcas*, and *H. equinus* (Figure 4q,r); and at the proximal articular surface of the radius in *B. bison*, *C. milneedwardsii*, *O. oreotragus*, and *G. dorcas* (Figure 4q,r). Trabecular bone is highly anisotropic and is oriented from the cranial and caudal borders to the extremity of the olecranon, where trabecular organisation is more random (Figure 4o–r). The apparent trabecular density is the highest for the heaviest species, especially in the cranial side of the olecranon, at the articular surface for the humerus, and on the caudal side of the olecranial neck (Figure 4o,q). It is lower at the top centre of the olecranon for some species, but without any special link with ecology, or size (Figure 4p–r). The species with the highest trabecular density in the olecranon seem to be *K. ellipsiprymnus*, then *C. milneedwardsii*, *B. javanicus*, and *B. bison*, which are mostly heavy animals living in a DCE or ME.

##### Hindlimb

###### Femur

**FIGURE 5:**
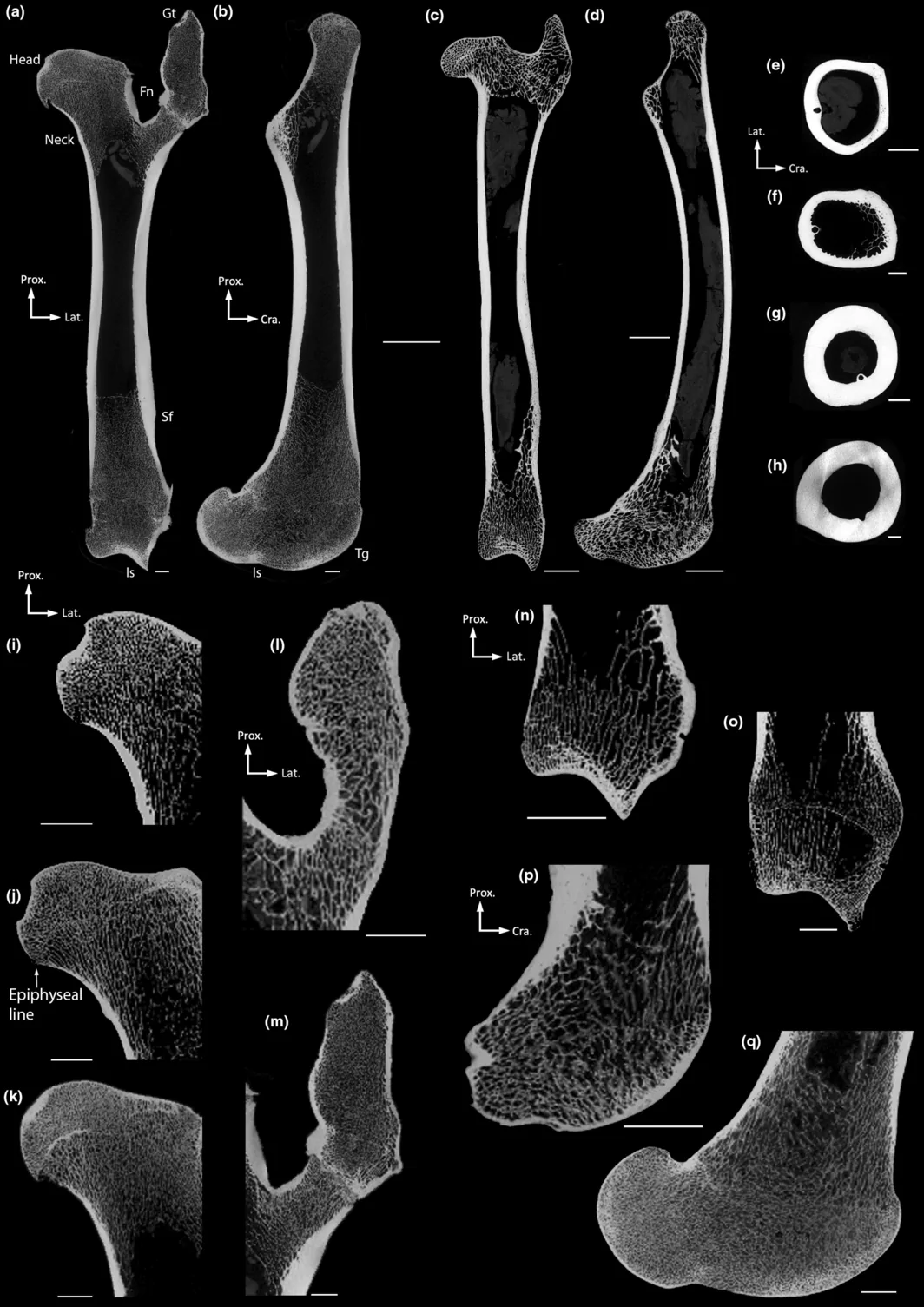
Sample of the virtual sagittal (SS), coronal (CS), and transverse (TS) sections made on the femora displaying the most striking mass or habitat adaptations. The whole bone: (a, b) Bos javanicus SS (a) and CS (b); (c, d) Gazella dorcas SS (c) and CS (d). Nutrient canal (TS): Gazella dorcas (e); Cephalophus silvicultor (f); Addax nasomaculatus (g); Bos javanicus (h). Zoom on the femoral head (CS): Cephalophus silvicultor (i); Connochaetes gnou (j); Boselaphus tragocamelus (k). Zoom on the greater tubercle (CS): Ammotragus lervia (l); Bos javanicus (m). Zoom on the intercondylar space (CS): Oreotragus oreotragus (n); Addax nasomaculatus (o). Zoom on the distal epiphysis (SS): Tetracerus quadricornis (p); Hippotragus equinus (q). Scale bar: 10 mm. Fn, femoral neck; Gt, greater trochanter; Is, intercondylar space; Sf, supracondylar fossa; Tf, trochanteric fossa; Tg, trochlear groove.

Most femoral transverse sections are round except in *B. bison*, *C. gnou*, and *C. silvicultor*, where they are oval (Figure 5f), and the compact bone thickness is rather homogeneous along the transverse sections, except in *B. javanicus* and *T. quadricornis*, where it is thicker on the caudal side (Figure 5h). Bovids with the relatively thickest compact bone are *A. lervia*, then *B. javanicus*, and *C. gnou*, which are some of the heaviest species of the sample. Thicker compact bone appears: under the neck in the lightest species (*T. quadricornis*, *O. oreotragus*, *G. dorcas*, *A. lervia*, *A. nasomaculatus*, and *C. silvicultor*); on the fovea capitis in heavy species living mostly in a covered environment (and *C. silvicultor*); and on the top of the femoral head in *B. javanicus* and *C. gnou* (Figure 5i–k). Thicker compact bone also appears at the femoral neck and on the greater trochanter in species living mostly in a LCE, DCE, or ME (Figure 5l,m). Most individuals present a thickening at the intercondylar space, except for species living mostly in a DCE and ME, which, instead, present a thickening on the lateral side of the intercondylar space (Figure 5n,o), on the trochlear groove, and on the caudal part of the condyle (Figure 5p,q). Underneath the lesser trochanter, trabecular bone is very anisotropic, going through the medial part of the femoral neck to the entirety of the femoral head border. The trabecular organisation is more random, closer to the compact bone layer of the femoral head, for all individuals (Figure 5i–k). Under the crest of the greater trochanter, trabeculae are very anisotropic and oriented in two preferential directions: (1) from the crest to the lateral tip of the femoral head, in a medio‐lateral axis, except in *B. javanicus* (Figure 5l,m), and to (2) the proximal extremity of the greater trochanter itself, along a proximo‐distal axis. On the lateral side of the greater trochanter, trabeculae are nearly proximo‐distally oriented in the heaviest bovids; they are mostly limited to the tip of the greater trochanter in the lightest individuals. On the medial and lateral sides from the distal metaphysis to the distal epiphysis, the orientation of the trabeculae is more anisotropic in heavier species. Within the distal epiphysis, they are almost perfectly oriented proximo‐distally in all individuals, although to a lesser extent on the cranial and caudal sides (Figure 5c,d,p,q).

Trabecular density in the proximal epiphysis appears maximal in the femoral head near the fovea capitis, especially in the heaviest bovids, but also in species living in a ME and globally in heavy bovids living in a DCE, while for the distal epiphysis, trabecular density is higher in heavier individuals (Figure 5q) and in OE and ME species.

###### Tibia

**FIGURE 6:**
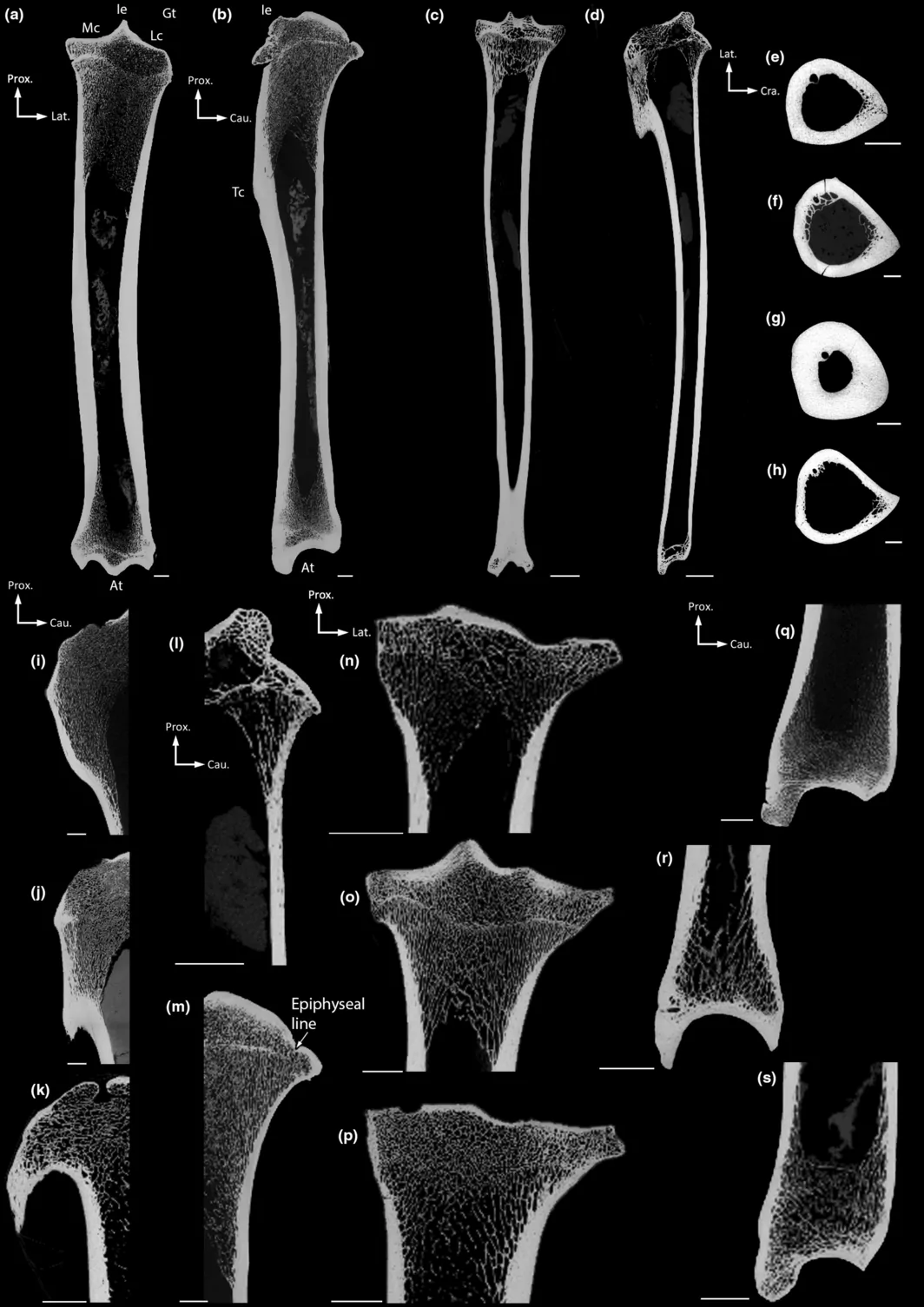
Sample of the virtual sagittal (SS), coronal (CS), and transverse (TS) sections made on the tibia displaying the most striking mass or habitats adaptations. The whole bone: (a, b) B. javanicus CS (a) and SS (b); (c, d) G. dorcas CS (c) and SS (d). Nutrient canal (TS): O. oreotragus (e); Capricornis milneedwardsii (f); Ammotragus lervia (g); Addax nasomaculatus (h). Zoom on the tibial crest (SS): Bison bison (i); Kobus ellipsiprymnus (j); Cephalophus silvicultor (k). Zoom on the tibial plate (SS): G. dorcas (l); Bos javanicus (m). Zoom on the intercondylar eminence (CS): Tetracerus quadricornis (n); Connochaetes gnou (o); Cephalophus silvicultor (p). Zoom on the distal epiphysis (CS): Bison bison (q); C. silvicultor (r); Capricornis milneedwardsii (s). Scale bar: 10 mm. At, articular surface of the talus; Ie, intercondylar eminence; Lc, lateral condyle; Mc, medial condyle; Tc, tibial crest.

All of the tibia transverse sections are quite triangular (Figure 6e–h), and compact bone thickness is rather homogeneous along the section, except for species living in a DCE or ME in which it is thicker on the cranial side (Figure 6h). Bovids with the thickest compact bone in transverse section are *A. lervia*, then *C. gnou*, and *B. javanicus*, which are some of the heaviest species of the sample. Thicker compact bone appears: in the proximal epiphysis at the tibial crest (Figure 6j) in species living mostly in a LCE, DCE, or ME; at the intercondylar eminence in some of the heaviest species of the sample and/or species living in a LCE, DCE, or ME; at the medial condyle (Figure 6o) in the heaviest species of the sample; and at the lateral condyle (Figure 6o) in species living in an OE or with a high body mass. In the distal epiphysis, a thick layer of compact bone occurs in most species (Figure 6q,s). Trabecular bone at the tibial crest is very anisotropic with two preferential orientations: (1) from the tibial crest to the intercondylar eminence cranio‐caudally (Figure 6j), in most heavy species living in a DCE; (2) nearly perfectly proximo‐distally from the tibial crest to the tibial tuberosity and then cranio‐caudally from the tibial tuberosity to the intercondylar eminence in the other taxa (Figure 6i). On the caudal side, trabeculae are very anisotropic and oriented nearly perfectly proximo‐distally from the insertion of the popliteus muscle to the tibial plate (lateral articular surface) in all bovids (Figure 6l,m). Trabecular organisation is more random at the summit of the tibia. On the medial and lateral sides, trabeculae are nearly perfectly proximo‐distal and more randomly oriented under the intercondylar eminence (Figure 6n,o). *C. silvicultor* presents a generally more random organisation (Figure 6k,p). In the distal epiphysis, trabeculae are weakly oriented along the proximo‐distal axis (Figure 6q,r), although to a much stronger extent in species living in a ME.

Species with the highest trabecular density are the heaviest species of the sample, which live in a DCE. Proximally, trabecular density is the highest at the tibial plate and the lowest at the caudal metaphysis, and distally, it is the highest at the articular surface with the talus and the lowest on the cranial side of the distal epiphysis.

#### 3.1.2 Thickness maps

**FIGURE 7:**
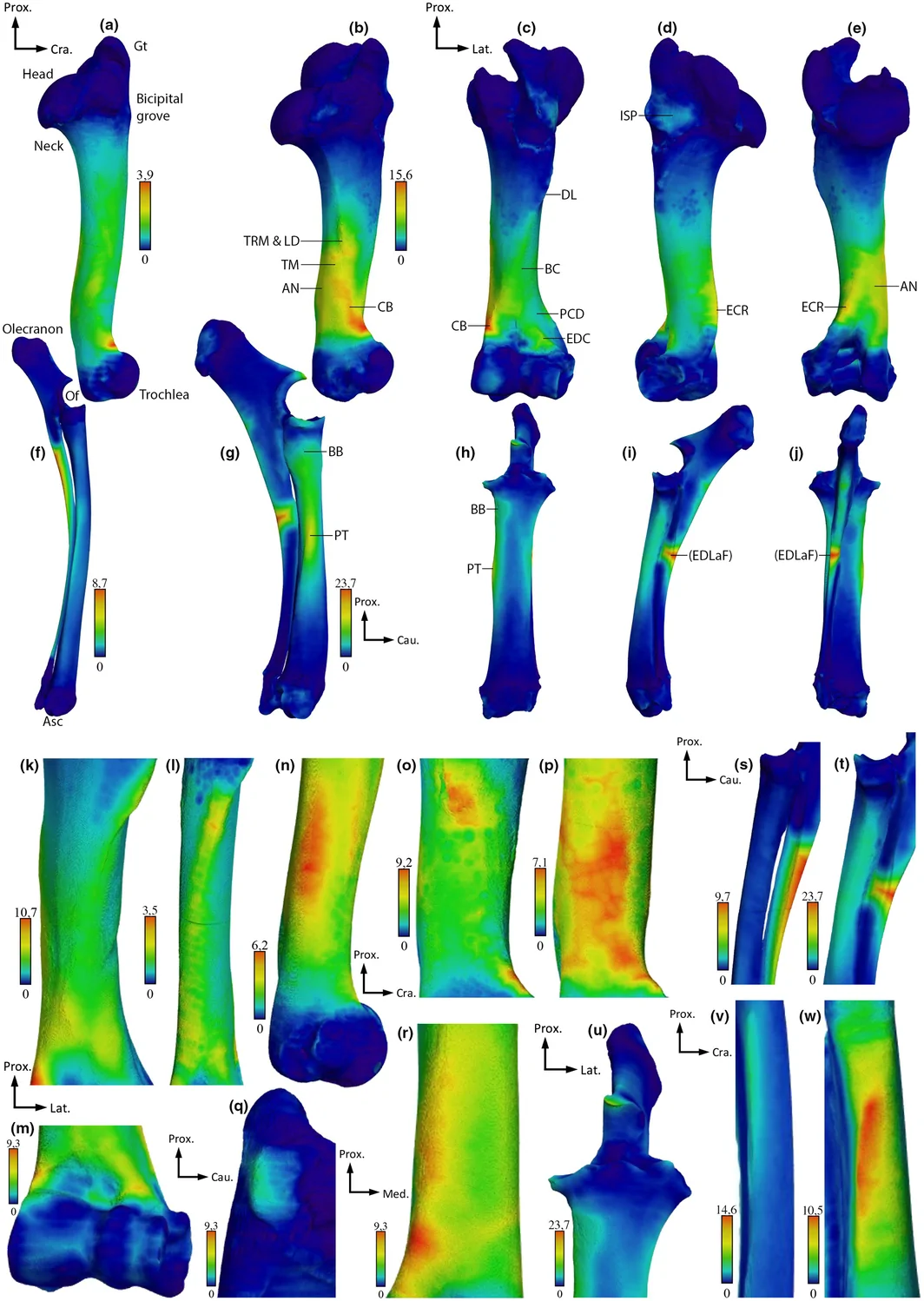
Cortical bone thickness maps made on the humeri and the radius‐ulna. Warm colors indicate thicker cortical bone, whereas cooler colors indicate thinner cortical bone (in mm). The whole humerus: (a) G. dorcas and (b) B. javanicus medial side (b), cranial side (c), lateral side (d), and caudal side (e). The whole radius‐ulna: (f) G. dorcas and (g) B. javanicus medial side (g), cranial side (h), lateral side (i), and caudal side (j). Zoom on the cranial side of the diaphysis of the humerus: B. bison (k); O. oreotragus (l). Zoom on the extensor medial digitorum: A. lervia (m). Zoom on the medial side of the diaphysis of the humerus: C. milneedwardsii (n); B. bison (o); H. equinus (p). Zoom on the infraspinatus: A. lervia (q). Zoom on the extensor carpi radialis: A. lervia (r). Zoom on the extensor digitorum lateralis: O. oreotragus (s); B. javanicus (t). Zoom on the olecranon fossa: B. javanicus (u). Zoom on the pronator teres: A. nasomaculatus (v); B. bison (w). AN, anconeus; Asc, articular surface with the carpus; BB, biceps brachii; BC, brachiocephalicus; CB, coracobrachialis; DL, deltoidus; ECR, extensor carpi radialis; EDC, medial extensor digitorum; EDLaF, extensor digitorum lateralis; Gt, greater trochanter; ISP, infraspinatus; Of, olecranon fossa; PCD, pectoralis descendens; PT, pronator teres; TM, triceps brachii caput mediale; TRM & LD, teres major tuberosity & latissimus dorsi.

**FIGURE 8:**
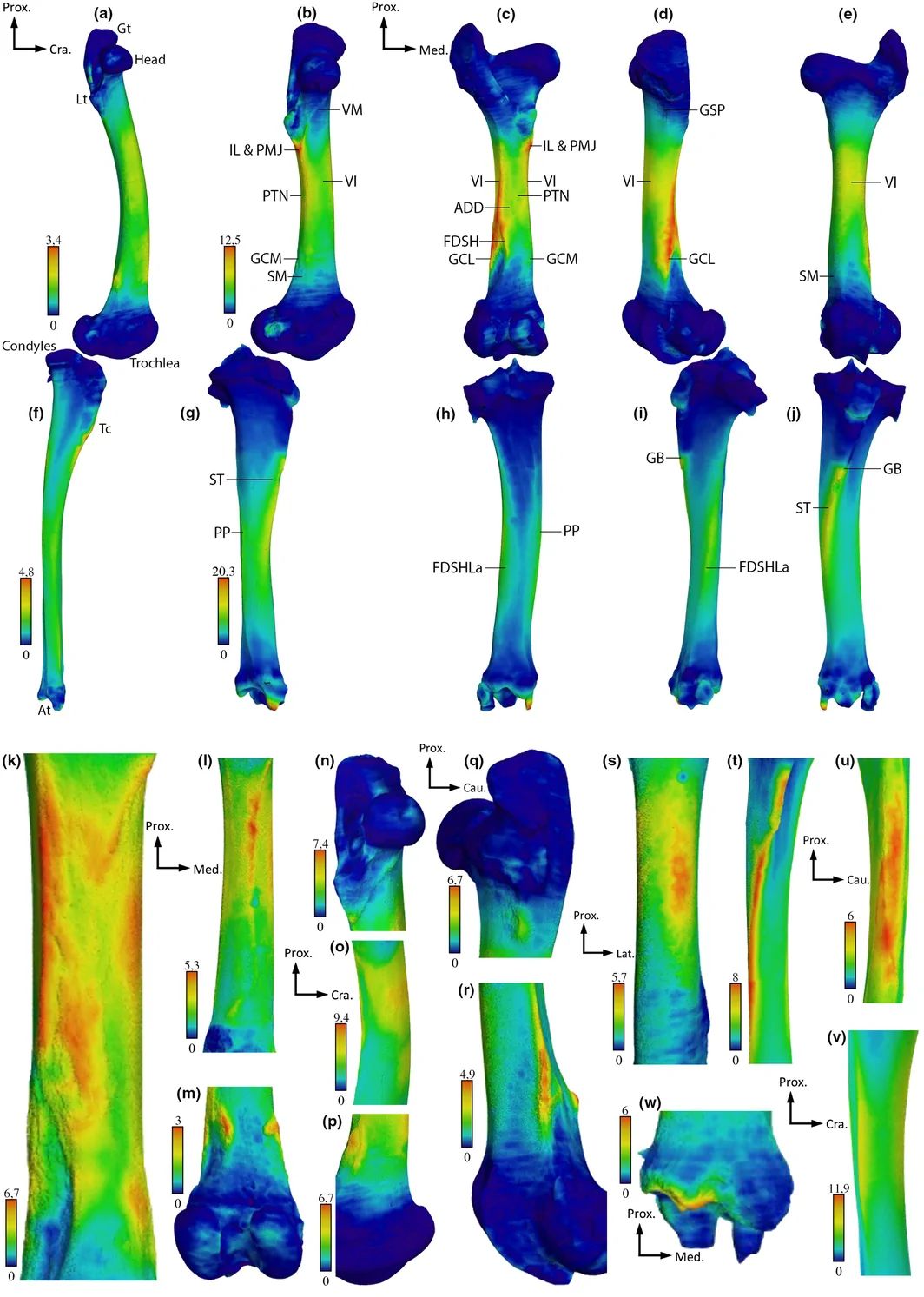
Cortical bone thickness maps made on the femur and tibia. Warm colors indicate thicker cortical bone, whereas cooler colors indicate thinner cortical bone (in mm). The whole femur: (a) G. dorcas and (b) B. javanicus medial side (b), cranial side (c), lateral side (d), and caudal side (e). The whole tibia: (f) Gazella Dorcas and (g) Bos javanicus medial side (g), cranial side (h), lateral side (i), and caudal side (j). Zoom on the caudal side: C. gnou (k); C. milneedwardsii (l). Zoom on the flexor digitorum superficialis and the gastrocnemius caput mediale: O. oreotragus (m). Zoom on the vastus medialis: A. lervia (n). Zoom on vastus intermedius: K. ellipsiprymnus (o); A. nasomaculatus (s). Zoom on the semimembranosus: C. gnou (p). Zoom on the gluteus superficialis: C. gnou (q). Zoom on the gastrocnemius caput laterale: T. quadricornis (r). Zoom on the gluteobiceps and the semitendinosus: C. milneedwardsii (t). Zoom on the flexor digitorum lateralis: A. nasomaculatus (u). Zoom on the gluteobiceps and the semitendinosus: K. ellipsiprymnus (v). Zoom on the distal epiphysis: A. nasomaculatus (w). ADD, adductores; At, articular surface of the talus; FDSH, flexor digitorum superficialis; FDSHLa, flexor digitorum lateralis; GB, gluteobiceps; GCL, gastrocnemius caput laterale; GCM, gastrocnemius caput mediale; GSP, gluteus superficialis; Gt, greater tubercle; IL & PMJ, iliacus and psoas major; Lt, lesser tubercle; PP, popliteus; PTN, pectineus; SM, semimembranosus; ST, semitendinosus; Tc, tibial crest; VI, vastus intermedius; VM, vastus medialis.

Compact bone thickness is globally maximal along the diaphysis, especially on the medial side, for the forelimb bones, while it is globally thicker on the cranial side for the hindlimb ones. It always appears the thinnest on the lateral side, except in the radius, for which it is thinnest on the medial side. Thickness progressively decreases away from the diaphysis up to the metaphyses, particularly clearly in the heaviest species (Figures 7a–e and 8a–e; Supporting Information: Data 5). Areas of cortical thickening appear to be mostly linked to areas of muscle insertion (Table 2).

**TABLE 2:** Summary of the insertions and actions of the muscles observed on the thickness maps according to Barone (2017b).

Bone	Muscle	Abb.	Action
Humerus	*Coracobrachialis*	CB	Shoulder adductor and internal rotation
	*Anconeus*	AN	Elbow extension
	*Teres major & Latissimus dorsi*	TRM & LD	Shoulder adductor and internal rotation
	*Extensor carpi radialis*	ECR	Wrist extension
	*Brachiocephalicus*	BC	Neck flexion and rotation, forelimb protraction
	*Pectoralis descendens*	PCD	Shoulder adduction
	*Deltoidus*	DL	Shoulder abduction and shoulder flexion when combined with teres major
	*Medial extensor digitorum*	EDC	Metacarpo/interphalangeal extension
	*Triceps brachii caput mediale*	TM	Elbow extension
	*Infraspinatus*	ISP	Shoulder abduction, stabilisation, and extension
Radius‐ulna	*Extensor digitorum lateralis*	EDLaF	Digits IV joints extension
	*Biceps brachii*	BB	Elbow and shoulder flexion
	*Pronator teres*	PT	Forearm flexor
Femur	*Vastus medialis*	VM	Knee extension
	*Vastus intermedius*	VI	Knee extension
	*Gastrocnemius caput mediale*	GCM	Ankle extension
	*Gastrocnemius caput laterale*	GCL	Ankle extension
	*Semimembranosus*	SM	Hip extension and knee flexion
	*Iliacus & Psoas major*	IL & PMJ	Hip flexion, hip external rotation, and lumbar region flexion
	*Pectineus*	PTN	Hip adduction, flexion, and internal rotation
	*Adductores*	ADD	Hip adduction
	*Flexor digitorum superficialis*	FDSH	Metacarpo/interphalangeal joints flexion
	*Gluteus superficialis*	GSP	Hip abduction
Tibia	*Gluteobiceps*	GB	Hip, knee, and ankle extension
	*Semitendinosus*	ST	Hip extension, ankle extension, and knee flexion
	*Lateral flexor digitorum superficialis*	FDSHla	Metacarpo/interphalangeal joints flexion
	*Popliteus*	PP	Knee flexion and internal rotation

##### Forelimb

###### Humerus

The zones of insertion of the *infraspinatus* (shoulder abduction, stabilisation, and extension, Figure 7d,p) and of the *deltoidus* (shoulder abduction and shoulder flexion when combined with the *teres major*, Figure 7c,k) only show a slight thickening of compact bone. By contrast, some muscle insertion areas appear clearly on the cartographies because of a thicker compact bone, like those of the *coracobrachialis* (shoulder adductor and internal rotation, Figure 7b,o), the *teres major* and the *latissimus dorsi* (shoulder adductor and internal rotation, Figure 7b,n), and of the *anconeus* (elbow extension, Figure 7m,o) muscles, especially in the heaviest species. Some local compact bone thickening seems to occur in species from a particular environment, as for the *infraspinatus*, the *extensor carpi radialis* (wrist extension, Figure 7d,r), and the *extensor digitorum* (metacarpo/interphalangeal extension, Figure 7c,m) in ME species or the *teres major*, the *latissimus dorsi* and the *triceps brachii caput mediale* (elbow extension, Figure 7j) in species living in an OE or LCE. Furthermore, even if no common pattern can be observed for species living in a DCE, they are slightly more similar to species living in a ME, with rather similar thickenings associated with the *extensor carpi radialis* and the *teres major* and *latissimus dorsi* insertions (Table S1.6).

###### Radius‐ulna

Fewer thickenings occur than in the humerus, but they can be related to muscular insertions. The *extensor digitorum lateralis*' insertion (Digit IV joints extension, Figure 7i,s) is visible in all species, but relatively thinner in heavy bovids, except those living in ME. The *pronator teres* (forearm flexor; Figure 7g,v) is associated with a thicker layer of compact bone in most heavy species. This pattern is particularly marked in the OE heavy species compared to those inhabiting a DCE or a ME. Thicker compact bone also occurs at the olecranon fossa in some of the heaviest bovids. No clear pattern permits us to distinguish species from different habitats.

##### Hindlimb

###### Femur

The insertion of the *vastus intermedius* (knee extension, Figure 8k,o,s), one of the largest muscles inserting on the femur (Barone, 2017b), is marked by a thickening in all bovids. Conversely, such a thickening for the *gluteus superficialis* (hip abduction, Figure 8q) occurs only in *C. gnou* and *A. lervia*, although a corresponding slight thickening is observed in species living in an OE, LCE, or ME relative to species living in a DCE. The heaviest species seem to show a stronger thickening of compact bone than the lightest bovids for the insertion of most of the flexors, especially for the *adductores* (hip adduction, Figure 8k) muscles, while most of the extensors' insertions do not show any thickening, even the *vastus intermedius* muscle, which has a large insertion in bovids (Barone, 2017a).

Some compact bone thickening also seems to occur mostly in species living in a ME at the insertion of the *gastrocnemius caput laterale* (ankle extension, Figure 8m), the *semimembranosus* (hip extension and knee flexion, Figure 8p), the *iliacus* and *psoas major* (hip flexion, hip external rotation, and lumbar region flexion, Figure 8k,l), and the *pectineus* (hip adduction, flexion, and internal rotation, Figure 8k), and, to a lesser extent, at the insertion of the *adductores*. A thickening can be observed at the *gastrocnemius caput mediale* for all habitats, but to a much lesser extent in ME species. Furthermore, the distribution of the compact bone thickness in the femur seems rather similar between species living in a DCE and species living in an OE, although with less intense thickenings in the former. Finally, species living in a DCE always display a relatively lower thickening at the *semimembranosus* muscle insertion than the others.

###### Tibia

The insertion of the *gluteobiceps* (hip, knee, and ankle extension, Figure 8t), one of the largest muscles inserting on the tibia, is associated with compact bone thickening in all bovids, except in *B. tragocamelus* and *H. equinus*, which both live in a DCE. Conversely, the thickening at the *popliteus* (knee flexion and internal rotation, Figure 8u) insertion is thicker only, and slightly, in a few species without regard to mass or habitat. As for the other bones, the insertions of flexor muscles seem to be marked by a higher thickening in heavier individuals. A thickening is observed for species living in a ME at the *semitendinosus* insertion (hip extension, ankle extension, and knee flexion, Figure 8v). Furthermore, even if it is not linked to any muscle insertion, a thickening is observed at the articular surface with the talus, more marked in species living in a DCE. As for the radius, no clear pattern exists to clearly distinguish species living in different environments.

### 3.2 Quantitative analyses

#### 3.2.1 Allometry

The cube root of the whole bone volume (WBV), used as a size estimate, is positively correlated with species mass for all bones (Table S2.1). In addition, size correlates with some microanatomical parameters for all bones except the humerus. Trabecular compactness (Tc) and the relative fraction of trabecular bone (%trab) are always correlated with size, whereas relative maximal compact thickness (RmaxT) is only correlated for the radius‐ulna. Size is also positively correlated with the first axis of principal component analysis (PC1) for the radius‐ulna only, and with the second axis (PC2) for the femur and tibia only (Supporting Information: Data 6).

#### 3.2.2 Variation of the microanatomical parameters between bones

**FIGURE 9:**
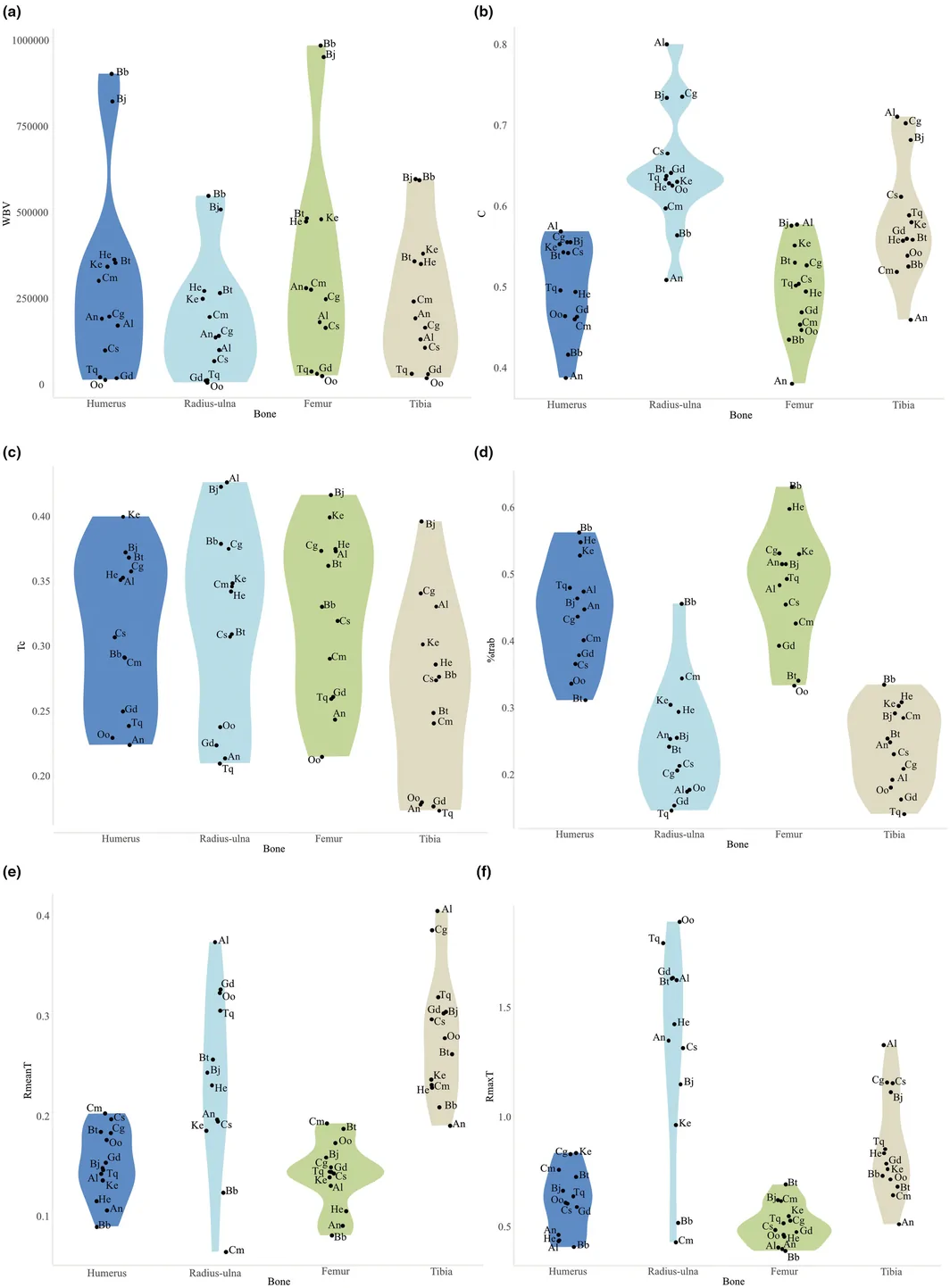
Violin plots comparing values obtained for the whole bone volume (a), bone compactness (b), trabecular compactness (c), relative fraction of trabecular bone (d), relative mean compact thickness (e), and relative maximal compact thickness (f) between the different sampled bone.

Stylopods (humerus and femur) seem to follow a similar trend for the WBV and all the microanatomical parameters, as well as zeugopods (radius‐ulna and tibia). However, stylopods and zeugopods seem to differ between them. For the WBV, while the lightest individuals tend to display similar values for all bones, once mass becomes greater than 80 kg (Table 1), stylopods display clearly increased values compared with zeugopods (Figure 9a). This is especially striking for the two heaviest bovids of our sample (more than 600 kg). Trabecular compactness (Tc) seems to be rather similar for all bones, except for the tibia, which always displays the lowest values for the same individual (Figure 9c). The relative portion of trabecular bone (%trab) is always greater for the stylopods (Figure 9d). The zeugopods always display greater values and variability for the global compactness (C), relative mean compact thickness (RmeanT), and relative maximal compact thickness (RmaxT) (Figure 9b,e,f).

The results of the Friedman test (Table 3) demonstrate significant differences between the bones (*p*‐value < 0.01) for all the microanatomical parameters. The following Nemenyi's post‐hoc comparisons (Figure S2.2) showed overall significant differences for the WBV between all bones, except for the tibia when compared, respectively, to the humerus and the femur. As observed on the violin plots, stylopods and zeugopods are significantly different for the global compactness and the relative fraction of trabecular bone, and the tibia is significantly different from the other bones for the trabecular compactness. Contrary to our expectations, differences between the tibia are not significant for the relative mean compact thickness and the relative maximal compact thickness, respectively, for the femur and the humerus. Otherwise, all the observed differences are significant.

**TABLE 3:** Summary of the Friedman test for the WBV and all microanatomical parameters.

	Statistic	*p*‐Value
WBV	30.7	<0.01
C	32.4	<0.01
Tc	20.2	<0.01
%trab	31.4	<0.01
RmeanT	24.5	<0.01
RmaxT	22.3	<0.01

#### 3.2.3 Variations of the microanatomical parameters with habitat

The WBV appears to differ significantly for species inhabiting an OE or DCE compared to those inhabiting an LCE or ME (Figure S3.1). However, this is likely due to the presence of Bj and Bb. Stylopod bones exhibit the lowest global compactness values (C) in individuals inhabiting open habitats or, to a lesser extent, mountainous environments (Figure S3.1). They also appear to exhibit greater variation in zeugopod bones, particularly in species inhabiting open environments. By contrast, species with heavy cover exhibit the least variation and the highest values. No clear distinctions can be made between the different habitats for all other microanatomical parameters (Figures S3.2 and S3.3). Overall, values seem to be higher and to vary less between individuals living in a DCE than in the other environments for %trab, RmeanT, and RmaxT, but the pattern remains unclear.

## 4 DISCUSSION

The present study focused on the bone microanatomy of the stylopod and zeugopod bones of bovids with different masses and habitats.

### 4.1 Microanatomical specialisations in bovid long bones

All bones of our sample show a medullary cavity (Figures 3, 4, 5, 6), although this cavity is very small in the ulna of all individuals (absent for *C. milneedwardsii* and filled with trabecular bone). As expected, the bone microanatomy of bovids undergoes changes in both the thickness of the compact bone layer and the trabecular architecture by influencing the orientation of the trabecular bone as well as the apparent bone volume fraction. Most of these appear linked with changes in mass (based on the qualitative and quantitative analyses) and habitat (only based on qualitative observations). These changes manifest in different ways depending on the bones and taxa, but general trends can be highlighted. As mass increases, the overall thickness of compact bone also increases relatively, particularly at the mid‐diaphysis, but also at muscle insertion areas, and so do the trabecular bone density and anisotropy, with trabeculae mainly oriented proximo‐distally. Habitat‐related differences are only distinguished in areas of muscle insertion or in specific articulation regions, such as the trochlea.

#### 4.1.1 Microanatomical specialisations to body mass in bovid long bones

The forelimb shows greater microanatomical specialisations for body weight support, especially in the humerus. Within the shoulder region, in heavy taxa, a thicker compact bone is observed in the humeral head with a more randomly oriented and denser trabecular bone, which then becomes more anisotropic, with a proximo‐distally orientation, more distally to the contact with the scapula. The isotropy of the trabecular bone in the humeral head might be explained by direct contact forces with the scapula acting with multiple orientations on the humeral head, and the need to absorb the associated shocks and redirect them to the diaphyseal compact bone through very anisotropic trabecular bone in the metaphyses, as observed in rhinoceroses (C.E. pers. obs.). In the bicipital groove and beneath the greater tubercle, trabeculae are more proximo‐distally oriented in heavy taxa. This pattern may be due to the passage of tendons (Barone, 2017a, 2017b) such as that of the *triceps brachii*, and of the insertion of strong muscles on the proximal epiphysis, such as those of the *supraspinatus* and the *infraspinatus*, whose insertion areas are furthermore enlarged on the external structure in heavy bovids (Barone, 2017a, 2017b; Etienne et al., 2021). Within the greater and the lesser tubercles, trabeculae are oriented more medio‐laterally, which could result from greater contact forces and/or forces exerted by the aforementioned muscles, as they contract, on the humeral ligament bridging the two tubercles (Barone, 2017b).

**FIGURE 10:**
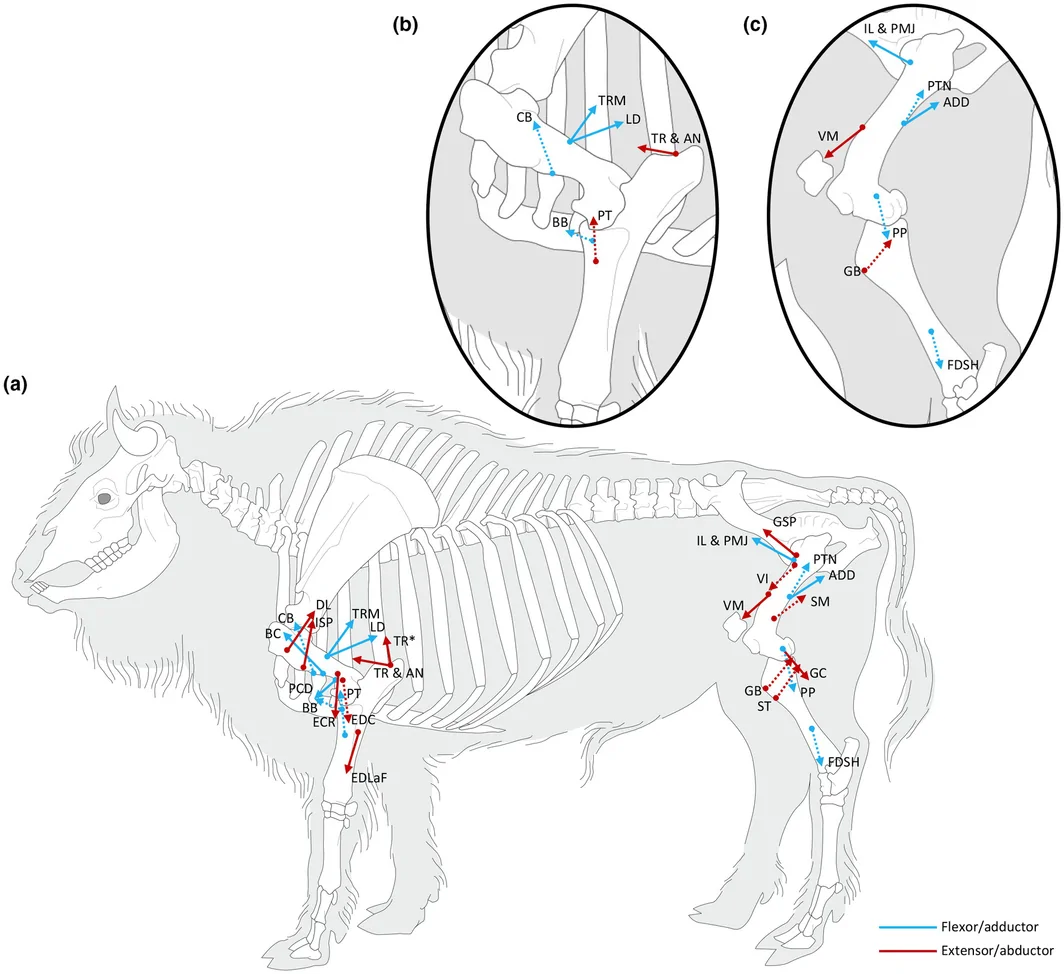
Summary of the forces exerted by the principal muscle groups on the limbs long bones occurring for heavy bovids on Bison bison MNHN‐ZM‐AC‐1902‐316 (a). Scheme of the limbs; since the triceps inserts on both the scapula and humerus, TR is for the medial head and lateral head of the triceps and TR* is for the long head of the triceps. Muscles insertions for which a thicker compact bone layer is visible in heavier bovids on (b) the forelimb and (c) the hindlimb modified from Barone (2017b). See Table 2 for the entire muscles names. Model obtained from www.archeozoo.org.

More distally in the humerus, a thicker compact bone layer is visible for the muscle insertions of the *teres major* and *latissimus dorsi*, which are the main flexors of the forelimb shoulder and perhaps of the *coracobrachialis* (flexor; shoulder adductor and internal rotation), as well as of the *anconeus* (extensor), although the scarcity of available dissections of non‐domesticated bovids complicates the identification of the insertions. However, the orientation of the trabecular bone in a more proximo‐distal direction suggests that it is more likely to have resulted from contact forces induced by a greater body weight than from the pulling effect of the muscles (for the *latissimus dorsi*, for example, trabecular bone would otherwise rather follow the muscle's tensile stress and thus display a more medio‐lateral orientation, Figure 10b). In the deltoidal tuberosity, where the *deltoidus* muscle, one of the main flexors of the forelimb, with the *teres major* and the *latissimus dorsi* (Barone, 2017b), inserts, a medio‐lateral orientation would have been expected here, given the pulling effect of these muscles, but instead, a proximo‐distal orientation is observed again resulting probably from contact forces with increasing mass. These observations support the idea that trabecular bone orientation in the humerus is strongly impacted by contact forces due to greater body weight, rather than mostly by the pulling effect of the muscles.

Forearm adaptations to increased ground reaction forces with increasing mass are associated with the insertion of the *pronator teres* and *biceps brachii* on the radius, and with the *triceps brachii* and the *anconeus* muscles on the ulna, which contribute to forearm extension and stabilisation by pulling on the olecranon which acts as a very efficient lever arm (Barone, 2017b). Adaptations to greater body weight are particularly visible on the radius, especially with regard to anisotropy in the insertion areas of the muscles mentioned above (*triceps brachii* and *anconeus*). However, the compact bone thickening is particularly marked on the caudal side of the olecranon, where highly anisotropic trabecular bone extends from the neck to the top. The top of the olecranon corresponds to the insertion area of the *triceps brachii* and of the *anconeus* and this structure probably results from a stronger pulling effect of these muscles in heavier species, which would be consistent with the thickening for the *triceps brachii* and the *anconeus* muscles insertions on the humerus (Figure 7). The absence, or at least reduced effect, of ground forces on the olecranon may be caused by the fusion of both zeugopods, which places more stress linked to body weight on the radius than on the olecranon, the last remaining element of the ulna. This is consistent with results on the external shape of the bones, which indicate a larger and more robust olecranon in heavier bovid species (Etienne et al., 2021). At the articulation with the carpus, compact bone appears to be thicker in heavy taxa, possibly in association with the insertion of the radial collateral ligament to prevent dislocation of the carpus (Takeda et al., 2023). Nevertheless, and contrary to the observed enlarged lateral part of the radial distal epiphysis in heavy bovids (Etienne et al., 2021), no microanatomical adaptative features were observed in association with the insertion of the extensors of the carpus and digits, suggesting that such morphological enlargement might be sufficient to dissipate the forces induced by muscles.

Moreover, the rather strong impact of mass on microanatomical structure in forelimb long bones is consistent with the forelimb's main role being to carry most of the weight (Amson & Bibi, 2021; Barone, 2017a; Biewener & Patek, 2018; Etienne et al., 2021; Schmitt, 1998; Scott, 1985). The associated microanatomical features nevertheless differ from those seen in heavier mammals such as rhinoceros and hippopotamus, which are characterized by a strong thickening of the cortex and the lack of a medullary space (Etienne et al., 2024; Houssaye et al., 2021; Houssaye, Waskow, et al., 2016).

The hindlimb also shows microanatomical specialisations adapted for bearing heavy loads, although these adaptations are less pronounced than in the forelimb. For instance, the cortex of the hindlimb long bones is relatively thinner than that of the forelimb bones (Supporting Information: Data 5). Compact bone is nevertheless clearly thicker in massive individuals at the *fovea capitis* (head of the femur), where the foveal ligament inserts, which is involved in limiting hip dislocation (O'Donnell et al., 2014). The greater tubercle of the femur displays trabeculae that are oriented even more proximo‐distally in heavy bovids, which is likely to reflect the enlarged muscular insertions observed for the *gluteus medius*, *vastus lateralis*, and *gluteus superficialis* (Etienne et al., 2021). These muscles are all key contributors to limb extension and body propulsion (Barone, 2017b). Moving distally along the limb, the *pectineus*, *iliacus*, and *psoas major* muscles insert on a thickened compact bone compared with the rest of the diaphysis, especially in heavy bovids (Figure 10). These adductor and protractor muscles also insert on the hip bone, helping to maintain limb stability. The *vastus intermedius* is a powerful muscle that plays a significant role in propulsion. It inserts onto the femur via a broad area characterised by a thickening of the cortex, which occurs in all bovids, but is particularly intense in heavier species. This is likely to help distribute forces efficiently as body mass increases (Barone, 2017b; Etienne et al., 2021).

A cortical thickening is observed close to the articulation between the femur and the tibia, particularly on the medial side of both bones. This may be a result of greater contact forces near the patella, where numerous ligaments and muscles insert or pass close by (Barone, 2017a, 2017b). Furthermore, a thicker compact bone layer and more proximo‐distally oriented trabeculae are observed in the heaviest species at the insertions of the *gluteobiceps* and *popliteus* muscles (respectively, an extensor and a flexor of the knee). Beyond their important role in cursorial abilities (explaining why we can also observe this pattern, although to a much lesser extent, in lighter species), these features may be additionally essential for maintaining an upright posture in heavier species, which can still gallop. Nevertheless, the *popliteus* muscle is, to our knowledge, a muscle that exerts relatively weak force compared with most muscles in bovids (Barone, 2017b). In addition to the aforementioned protractor muscles, Etienne et al. (2021) suggested that the *flexor digitorum* muscle may play a significant role in maintaining an upright posture, particularly in heavy bovids. This muscle contributes to plantarflexion and supports upright posture, and strong limb protraction thanks to its enlarged insertion zone in gross morphology. Consequently, the observed microanatomical adaptations, such as a thicker compact bone and proximo‐distally aligned trabeculae, may help dissipate the increased contact forces caused by greater body weight by redirecting tensile stresses into the compact bone. The fewer microanatomical adaptations to body mass compared with the forelimb are consistent with the hindlimb's main role of propelling the body forward (Barone, 2017a; Doube et al., 2011; Hanot et al., 2017; Hildebrand, 1998; Lessertisseur & Saban, 1967).

#### 4.1.2 Microanatomical specialisations to habitat in bovid long bones

Open (OE) and light cover (LCE) taxa display relatively few common microanatomical features, suggesting fewer shared dominant constraints acting on their long bones compared with taxa in other habitats. This pattern indicates that the observed morphological enlargement of the articulations in these taxa (Etienne et al., 2021) might be sufficient to withstand the higher forces encountered during high‐speed locomotion in OE, thus reducing the need for additional microanatomical adaptations. Although the distinction between OE and LCE based on microanatomical features appears rather ambiguous, a marked separation is evident between these habitats and both dense cover (DCE) and mountainous (ME) environments. In DCE and ME, species exhibit a thicker compact layer at the greater tubercle of the humerus, where key muscles such as the *pectoralis ascendans*, *supraspinatus*, and *infraspinatus* insert (Figure 7q). This thicker layer is associated with proximo‐distally oriented trabecular bone in the humeral proximal portion and a higher trabecular bone density (Figure 3k,l). The muscles, tendons, and ligaments connected to this area, or those that pass between the two tubercles, are crucial for maintaining an upright posture (Barone, 2017a, 2017b). This stabilising adaptation is also observed in the forearm (e.g., on the medial side of the radius, at the radial styloid process, and on the cranial side of the ulna, at the articular surface with the humerus, where the *flexor digitorum lateralis* inserts) and seems to be particularly important for navigating cover‐dense or steep environments. Steep and overgrown environments require more manoeuvring to navigate them, which might induce more robust muscles as well as greater tensile forces, leading to a need for greater force dissipation, as suggested by the generally higher trabeculae density exhibited by these species than those from open habitats (Figure 4p,q). By contrast, the humeral head and the trochlea display more isotropic trabeculae, which may aid in distributing ground reaction forces in multiple directions, potentially preventing injury in uneven terrain. Furthermore, similarities between DCE and ME may reflect the higher demand for manoeuvrability, likely to avoid carpal twisting (Takeda et al., 2023) and reduce the risk of dislocation. Beyond these shared traits, dense cover species tend to have a thicker compact bone layer at the humeral greater tubercle and a reduced medullary space in the ulnar olecranon (Figures 3k,l and 4o–r), likely as an adaptation for enhanced shoulder stabilisation and manoeuvrability. ME species' specific adaptations are all the more visible at the insertions of the *infraspinatus* (for shoulder abduction, stabilisation, and extension), *extensor carpi radialis* (for wrist extension), and *medial extensor digitorum* (for metacarpal/interphalangeal extension) muscles, which feature a thicker compact bone on the humerus, distinguishing them from taxa from all other environments.

In contrast to the forelimb, the hindlimb exhibits greater similarity among species from OE, LCE, and DCE. Near the knee, they all display a thicker, compact bone and a higher trabecular density in the lateral condyle. This feature may serve different functions across habitats: In open habitats, it might support stronger ligament or tendon insertions (such as the patellar tendon) to prevent dislocation during high‐speed transverse galloping, which requires robust ligaments due to frequent use of this gait (Biancardi & Minetti, 2012). Meanwhile, in covered environments, this thickening may enhance manoeuvrability in dense vegetation. Additionally, this thicker compact bone might relate to knee asymmetry, which is more pronounced in galloping species from open environments (Janis et al., 2012). In both LCE and DCE, species exhibit a thicker compact bone in the femoral head's *fovea capitis*, where the ligament of the head of the femur inserts, along with a higher trabecular density (Figure 5i–k). This structural reinforcement might help better resist forces during large‐amplitude hip movements, supporting greater freedom of motion as facilitated by the enlarged femoral head (Etienne et al., 2021; O'Donnell et al., 2014). In the tibia, species across covered habitats generally exhibit thicker compact bone where the patellar ligament inserts, as well as at the intercondylar eminence, where the meniscus and other ligaments are located. This adaptation could help stabilize the limb and better resist forces on uneven substrates by increasing resistance while the limb is close to the body during wide‐ranging movements.

Species in LCE do not show a thickened compact bone at the *gluteobiceps* muscle insertion. However, the insertion of this muscle seems to be associated with microanatomical reinforcement in taxa from all the other habitats, suggesting different pressures and needs (Tables S1.5 and S1.6). It may be the result of a reduced necessity for efficient galloping, being less crucial in this environment, and a necessity for efficient hip, knee, and ankle extension being less prevalent than in DCE and ME.

Species in DCE show a thicker compact bone at the articulation with the talus, possibly for better stress dissipation near the cruciate ligament during rotational movements (Figure 6q–s).

ME species show the strongest hindlimb specialisation, with a notably high density in the femoral head and intercondylar space, even compared to species in DCE. The femur of ME species supports muscles critical for supporting an upright posture and propulsion, such as the *gastrocnemius caput laterale*, *semimembranosus*, *iliacus*, *psoas major*, *pectineus*, adductores (Figures 5 and 8; Table S1.6), promoting stability on steep terrain. The tibia in these species has a more compact intercondylar eminence, proximo‐distally oriented trabeculae, and a strengthened insertion for the *semitendinosus* muscle, which might facilitate ‘locking’ the limb when necessary, while also dissipating ground reaction forces efficiently.

#### 4.1.3 Shared adaptations in relation to mass and habitat

Despite these differences, heavy bovids seem to share similarities with DCE and ME species, even if fewer similarities can also be found with OE and LCE species, regarding the inner structure. It is unexpected regarding the results on the external shape of the bones (Etienne et al., 2021), for which there appear to be greater similarities between heavy bovids and species living in an OE. The similarities are characterized here by a thicker compact bone and changes in the trabecular orientation as well as a higher apparent trabecular density (e.g., Figures 3p,r and 6o,p; Figure S3.2). It is especially true for the insertion area of flexor muscles but can also be seen in articulation areas such as the head of the femur. It could be a response to the need for stronger muscle insertions for flexor muscles, such as the *brachiocephalicus* and the *iliacus* and *psoas major*, and of reinforcing articulation areas, ensuring stability and better forces dissipating in response to a greater mass or denser and steeper environments. It could also result from a need for greater manoeuvrability, which would imply a more effective use of muscles that are not the usual propulsory muscles used during galloping in OE.

Bone areas showing shared adaptations between mass and habitat are mostly on the forelimb. It is especially true for the humerus of OE and LCE, which display a thicker compact bone where the *triceps brachii caput laterale*, the *teres major* and *latissimus dorsi*, and the *pronator teres* insert. These adaptations are strongly marked for the heavier bovids living in these environments, which additionally exhibit a thinner compact bone on the cranial side of the radius and on its articular surface with the humerus. For species living in a denser or steeper environment, similarities with heavy bovids are very visible at the insertions of the *pectoralis ascendans* and of the *infraspinatus*, which allow medio‐lateral limb movement. It could thus be an adaptation to an environment that is complex to navigate, such as dense vegetation or steep terrain (Kappelman, 1988), and may be highly required to stabilise the shoulder, even more for heavy species living in those environments. Contrary to our expectations regarding the outer shape, changes in extensors' insertion areas, and especially of the *extensor digitorum lateralis*, do not occur in heavy bovids for which the area of insertion of the muscle is relatively enlarged (Etienne et al., 2021). However, the insertion area of this extensor is quite visible on ME species regardless of mass. Thus, it could mean that the strongest needs of these muscles are to lock the joint in place or to prevent dislocation in a steep environment, while digits are widely spread (Takeda et al., 2023), different constraints leading thus to similar adaptations.

### 4.2 Limits

Due to the small size of our sample and the natural sensitivity of PGLS to sampling bias across the phylogeny (Pearse et al., 2025), we dismissed any test involving the impact of phylogeny. This impact should therefore be investigated in more extensive studies.

In addition, because of the wish to describe the microanatomy of all the stylopod and zeugopod bones for each taxon, our study is limited by the number of specimens. Only one specimen for each of the 13 taxa may not be representative of the species. Additionally, our study shows a complexity of patterns related to habitat and body mass, as some traits are influenced by both factors. Nevertheless, we were able to identify clear qualitative patterns (a generally thicker compact bone and an increasement of a more anisotropic and dense trabecular bone) confirmed by quantitative microparameters (Tc, %trab, and RmaxT) associated with mass. The results for the humerus were, however, quite surprising, as they were not significant, although the *p*‐value was close to 0.05, whereas the forelimb bones are the main weight‐bearing bones, and the impact of mass was statistically significant for all the other bones. We would have expected less influence from the hindlimb than from the humerus, as highlighted by Etienne et al. (2021). It may be due to structural differences among the four largest taxa from our sample. For instance, while *Kobus ellipsiprymnus* and *Hippotragus equinus* have equal limb heights, *Bos bison* and *Bos javanicus* have forelimbs that are higher than hindlimbs, accompanied by more developed shoulder musculature and a well‐developed scapula with an enlarged supraspinatus process (Castelló, 2016; Table S1.1). These anatomical differences could have an impact on the humerus microanatomy and might contribute to diminishing the impact of size in our analyses. Overall, body mass shows limited influence on microanatomical parameters compared with the outer bone structure previously described (Etienne et al., 2021), but it was tested here on a smaller sample. Now that the microanatomical specialisations have been described in various taxa, and that regions of interest for morphofunctional interpretations have been identified, a larger sample would thus naturally enable us to better test the impact of size and to decipher it from the impact of habitat.

Some specimens used in this study come from zoos, which are known to limit movement abilities and might alter bone microstructure (Kilbourne & Hutchinson, 2019; Parsi‐Pour & Kilbourne, 2020; Scheidt et al., 2019; Turner, 1998) (Supporting Information: Data 6, Figure S1). However, some previous studies have also shown that when major constraints apply to bones, their microanatomy can be quite conserved despite very different living conditions (e.g., in wild vs. zoo vs. circus black rhinos; C.E. pers. obs.). Here, certain specimens showed surprising microanatomies, such as *B. bison*, with a surprisingly thin compact bone and low trabecular density. Greater bone porosity in captive specimens may notably result from osteoporosis, which has a higher prevalence in females (Bonucci & Ballanti, 2014; Epker et al., 1965; Epker & Frost, 1965), and this bison was a female, which might have caused a bias.

## 5 CONCLUSION

This study provides the first detailed analysis of the microanatomical adaptations in the stylopod and zeugopod bovid limb bones, highlighting how bone structure responds to functional demands in these taxa. In heavier bovids, particularly in the forelimb, a thicker compact bone layer and more proximo‐distally oriented trabeculae support increased mass, with muscle insertions further shaping bone structure to enhance stability. The forelimb exhibits more significant changes due to its role in supporting body weight, while habitat influences are more evident in the hindlimb, with subtle bone variations reflecting specific functional needs. The combined effects of mass and habitat present challenges for deciphering the form–function relationships and thus for future paleontological interpretations, supporting a need for analysing a larger, more diverse sample in future research to deepen our understanding of bovid bone adaptations as well as the phylogenetic effect.

## AUTHOR CONTRIBUTIONS

A.H. and C.E. designed the study. A.H. and C.E. did the bone data acquisition. M.P. conducted the segmentation. M.P. conducted the analyses. A.H., C.E., and M.P. prepared the figures and drafted the manuscript. All authors contributed to the final manuscript, read it, and approved it.

## Supporting information


Appendix S1.

## Data Availability

The data that support the findings of this study are available from the corresponding author upon reasonable request.

## References

[joa70140-bib-0001] Alexander, R.M.N. (1977) Allometry of the limbs of antelopes (Bovidae). Journal of Zoology, 183(1), 125–146. Available from: 10.1111/j.1469-7998.1977.tb04177.x

[joa70140-bib-0003] Allaire, J. (2011) RStudio.

[joa70140-bib-0004] Amson, E. & Bibi, F. (2021) Differing effects of size and lifestyle on bone structure in mammals. BMC Biology, 19, 87. Available from: 10.1186/s12915-021-01016-1 33926429 PMC8086358

[joa70140-bib-0005] Arganda‐Carreras, I. , Kaynig, V. , Rueden, C. , Eliceiri, K.W. , Schindelin, J. , Cardona, A. et al. (2017) Trainable Weka segmentation: a machine learning tool for microscopy pixel classification. Bioinformatics, 33(15), 2424–2426. Available from: 10.1093/bioinformatics/btx180 28369169

[joa70140-bib-0006] Bader, C. , Böhmer, C. , Abou, M. & Houssaye, A. (2022) How does bone microanatomy and musculature covary? An investigation in the forelimb of two species of martens (*Martes foina*, *Martes martes*). Journal of Anatomy, 241, 145–167. Available from: 10.1111/joa.13645 35266144 PMC9178392

[joa70140-bib-0007] Barone, R. (2017a) Anatomie comparée des. mammifères domestiques. Vol. Tome 1.‐Ostéologie. Paris: Vigot Frères.

[joa70140-bib-0008] Barone, R. (2017b) Anatomie comparée des mammifères domestiques. Vol. Tome 2: Arthrologie et myologie. Paris: Vigot Frères.

[joa70140-bib-0009] Biancardi, C. & Minetti, A. (2012) Biomechanical determinants of transverse and rotary gallop in cursorial mammals. Journal of Experimental Biology, 215(23), 4144–4156. Available from: 10.1242/jeb.073031 22933611

[joa70140-bib-0010] Bibi, F. (2013) A multi‐calibrated mitochondrial phylogeny of extant Bovidae (Artiodactyla, Ruminantia) and the importance of the fossil record to systematics. BMC Evolutionary Biology, 13(1), 166. Available from: 10.1186/1471-2148-13-166 23927069 PMC3751017

[joa70140-bib-0011] Biewener, A. & Patek, S. (2018) Animal locomotion, 2nd edition. New York, USA; Oxford, UK: Oxford University Press.

[joa70140-bib-0012] Bishop, P. , Hocknull, S. , Clemente, C. , Bishop, P.J. , Hocknull, S.A. , Clemente, C.J. et al. (2018) Cancellous bone and theropod dinosaur locomotion. Part I—an examination of cancellous bone architecture in the hindlimb bones of theropods. PeerJ, 6, e5778. Available from: 10.7717/peerj.5778 30402347 PMC6215452

[joa70140-bib-0014] Bonucci, E. & Ballanti, S. (2014) Osteoporosis—bone remodeling and animal models. Toxicologic Pathology, 42(6), 957–969. Available from: 10.1177/0192623313512428 24285673

[joa70140-bib-0016] Canoville, A. & Laurin, M. (2010) Evolution of humeral microanatomy and lifestyle in amniotes, and some comments on palaeobiological inferences. Biological Journal of the Linnean Society, 100(2), 384–406. Available from: 10.1111/j.1095-8312.2010.01431.x

[joa70140-bib-0018] Castelló, J. (2016) Bovids of the world; antelopes, gazelles, cattle, goats, sheep, and relatives. Princeton field guildes, 1st edition. Princeton, USA: Princeton University Press.

[joa70140-bib-0020] Davies, T. & Stock, J. (2014) The influence of relative body breadth on the diaphyseal morphology of the human lower limb. American Journal of Human Biology, 26(6), 822–835. Available from: 10.1002/ajhb.22606 25163696

[joa70140-bib-0022] Doube, L. , Kłosowski, M. , Wiktorowicz‐Conroy, A. , Hutchinson, J. & Shefelbine, S. (2011) Trabecular bone scales allometrically in mammals and birds. Proceedings. Biological sciences, 278(1721), 3067–3073. Available from: 10.1098/rspb.2011.0069 21389033 PMC3158937

[joa70140-bib-0023] Duffield, L. (1973) Aging and sexing the post‐cranial skeleton of bison. Plains Anthropologist, 18(60), 132–139.

[joa70140-bib-0024] Epker, B. & Frost, H. (1965) A histological study of remodeling at the periosteal, haversian canal, cortical endosteal, and trabecular endosteal surfaces in human rib. The Anatomical Record, 152(2), 129–135. Available from: 10.1002/ar.1091520203 5842156

[joa70140-bib-0025] Epker, B. , Kelin, M. & Frost, H. (1965) Magnitude and location of cortical bone loss in human rib with aging. Clinical Orthopaedics and Related Research, 41, 198–203. 5832732

[joa70140-bib-0026] Etienne, C. , Filippo, A. , Cornette, R. & Houssaye, A. (2021) Effect of mass and habitat on the shape of limb long bones: a morpho‐functional investigation on Bovidae (Mammalia: Cetartiodactyla). Journal of Anatomy, 238(4), 886–904. Available from: 10.1111/joa.13359 33210307 PMC7930763

[joa70140-bib-0027] Etienne, C. , Houssaye, A. , Fagan, M. & Hutchinson, J. (2024) Estimation of the forces exerted on the limb long bones of a White rhinoceros (*Ceratotherium simum*) using musculoskeletal modelling and simulation. Journal of Anatomy, 245(2), 240–257. Available from: 10.1111/joa.14041 38558391 PMC11259748

[joa70140-bib-0029] Frost, H. (1994) Wolff's law and bone's structural adaptations to mechanical usage: an overview for clinicians. The Angle Orthodontist, 64(3), 175–188. Available from: 10.1043/0003-3219(1994)064<0175:WLABSA>2.0.CO;2 8060014

[joa70140-bib-0030] Germain, D. & Laurin, M. (2005) Microanatomy of the radius and lifestyle in amniotes (Vertebrata, Tetrapoda). Zoologica Scripta, 34(4), 335–350. Available from: 10.1111/j.1463-6409.2005.00198.x

[joa70140-bib-0031] Gônet, J. , Laurin, M. & Hutchinson, J.R. (2023) Evolution of posture in amniotes–diving into the trabecular architecture of the femoral head. Journal of Evolutionary Biology, 36, 1150–1165. Available from: 10.1111/jeb.14187 37363887

[joa70140-bib-0032] Gould, S. (2002) The structure of evolutionary theory. Harvard, USA: Harvard University Press.

[joa70140-bib-0033] Grand, T. (1997) How muscle mass is part of the fabric of behavioral ecology in East African bovids (*Madoqua*, *Gazella*, *Damaliscus*, *Hippotragus*). Anatomy and Embryology, 195(4), 375–386. Available from: 10.1007/s004290050058 9108204

[joa70140-bib-0034] Gregory, W. (1912) Notes on the principles of quadrupedal locomotion and on the mechanism of he limbs in hoofed animals. Annals of the New York Academy of Sciences, 22(1), 267–294. Available from: 10.1111/j.1749-6632.1912.tb55164.x

[joa70140-bib-0035] Hall, B. (2008) Fins into limbs: evolution, development, and transformation. Chicago, USA: University of Chicago Press.

[joa70140-bib-0036] Hanot, P. , Herrel, A. , Guintard, C. & Cornette, R. (2017) Morphological integration in the appendicular skeleton of two domestic taxa: the horse and donkey. Proceedings of the Royal Society B: Biological Sciences, 284(1864), 20171241. Available from: 10.1098/rspb.2017.1241 PMC5647294 28978726

[joa70140-bib-0037] Harbers, H. , Zanolli, C. , Cazenave, M. , Theil, J.C. , Ortiz, K. , Blanc, B. et al. (2020) Investigating the impact of captivity and domestication on limb bone cortical morphology: an experimental approach using a wild boar model. Scientific Reports, 10(1), 19070. Available from: 10.1038/s41598-020-75496-6 33149160 PMC7643176

[joa70140-bib-0038] Hildebrand, M. & Goslow, G. (1998) Analysis of vertebrate structure, 5th edition. New York: Wiley.

[joa70140-bib-0039] Houssaye, A. , Fernandez, V. & Billet, G. (2016) Hyperspecialization in some south American endemic ungulates revealed by long bone microstructure. Journal of Mammalian Evolution, 23(3), 221–235. Available from: 10.1007/s10914-015-9312-y

[joa70140-bib-0040] Houssaye, A. , Martin, F. , Boisserie, J.‐R. & Lihoreau, F. (2021) Paleoecological inferences from long bone microanatomical specializations in Hippopotamoidea (Mammalia, Artiodactyla). Journal of Mammalian Evolution, 28(3), 847–870. Available from: 10.1007/s10914-021-09536-x

[joa70140-bib-0041] Houssaye, A. , Perthuis, A. & Houée, G. (2022) Sesamoid bones also show functional adaptation in their microanatomy—the example of the patella in Perissodactyla. Journal of Anatomy, 240(1), 50–65. Available from: 10.1111/joa.13530 34402049 PMC8655183

[joa70140-bib-0042] Houssaye, A. , Sander, M. & Klein, A. (2016) Adaptive patterns in aquatic amniote bone microanatomy—more complex than previously thought. Integrative and Comparative Biology, 56(6), 1349–1369. Available from: 10.1093/icb/icw120 27794536

[joa70140-bib-0044] Houssaye, A. , Taverne, M. & Cornette, R. (2018) 3D quantitative comparative analysis of long bone diaphysis variations in microanatomy and cross‐sectional geometry. Journal of Anatomy, 232(5), 836–849. Available from: 10.1111/joa.12783 29411354 PMC5879956

[joa70140-bib-0045] Houssaye, A. , Waskow, K. , Hayashi, S. , Cornette, R. , Lee, A. & Hutchinson, J. (2016) Biomechanical evolution of solid bones in large animals: a microanatomical investigation. Biological Journal of the Linnean Society, 117(2), 350–371. Available from: 10.1111/bij.12660

[joa70140-bib-0046] Janis, C. , Shoshitaishvili, B. , Kambic, R. & Figueirido, B. (2012) On their knees: distal femur asymmetry in ungulates and its relationship to body size and locomotion. Journal of Vertebrate Paleontology, 32(2), 433–445. Available from: 10.1080/02724634.2012.635737

[joa70140-bib-0047] Kappelman, J. (1988) Morphology and locomotor adaptations of the bovid femur in relation to habitat. Journal of Morphology, 198(1), 119–130. Available from: 10.1002/jmor.1051980111 3199446

[joa70140-bib-0048] Kilbourne, B. & Hutchinson, J. (2019) Morphological diversification of biomechanical traits: mustelid locomotor specializations and the macroevolution of long bone cross‐sectional morphology. BMC Evolutionary Biology, 19(1), 37. Available from: 10.1186/s12862-019-1349-8 30700269 PMC6354431

[joa70140-bib-0049] Lambers, F. , Schulte, A. , Kuhn, G. , Webster, D. & Müller, R. (2011) Mouse tail vertebrae adapt to cyclic mechanical loading by increasing bone formation rate and decreasing bone resorption rate as shown by time‐lapsed in vivo imaging of dynamic bone morphometry. Bone, 49(6), 1340–1350. Available from: 10.1016/j.bone.2011.08.035 21964411

[joa70140-bib-0050] Lee, T. & Taylor, D. (2003) Quantification of ovine bone adaptation to altered load: morphometry, density, and surface strain. European Journal of Morphology, 41(3–4), 117–125. 16225262

[joa70140-bib-0052] Lessertisseur, J. & Saban, R. (1967) Squelette appendiculaire. Traite de Zoologie Tome XVI, 1er Fasc: 709–1078.

[joa70140-bib-0053] Maas, H. , Baan, G. & Huijing, P. (2004) Muscle force is determined also by muscle relative position: isolated effects. Journal of Biomechanics, 37(1), 99–110. Available from: 10.1016/S0021-9290(03)00235-5 14672573

[joa70140-bib-0054] Maddison, W. & Maddison, D. (2023) Mesquite: a modular system for evolutionary analysis. Version 3.81. Available from: http://www.mesquiteproject.org [Accessed 12th May 2023].

[joa70140-bib-0056] Mallet, C. , Houssaye, A. , Cornette, R. & Billet, G. (2021) Long bone shape variation in the forelimb of Rhinocerotoidea: relation with size, body mass and body proportions. Zoological Journal of the Linnean Society, 196, zlab095. Available from: 10.1093/zoolinnean/zlab095

[joa70140-bib-0057] Meloro, C. , Elton, S. , Louys, J. , Bishop, L. & Ditchfield, P. (2013) Cats in the Forest: predicting habitat adaptations from humerus morphometry in extant and fossil Felidae (Carnivora). Paleobiology, 39(3), 323–344. Available from: 10.1666/12001

[joa70140-bib-0059] Moyà‐Solà, S. , Cardona, J. & Köhler, M. (2022) *Ebusia moralesi* n. gen. nov. sp, a new endemic caprine (Bovidae, Mammalia) from the Neogene of Eivissa Island (Balearic Islands, Western Mediterranean): evolutionary implications. Historical Biology, 34(8), 1642–1659. Available from: 10.1080/08912963.2022.2060099

[joa70140-bib-0060] Nakajima, Y. , Hirayama, R. & Endo, H. (2014) Turtle humeral microanatomy and its relationship to lifestyle. Biological Journal of the Linnean Society, 112(4), 719–734. Available from: 10.1111/bij.12336

[joa70140-bib-0061] Nikander, R. , Kannus, P. , Rantalainen, T. , Uusi‐Rasi, K. , Heinonen, A. & Sievänen, H. (2010) Cross‐sectional geometry of weight‐bearing tibia in female athletes subjected to different exercise loadings. Osteoporosis International, 21(10), 1687–1694. Available from: 10.1007/s00198-009-1101-0 19921084

[joa70140-bib-0062] O'Donnell, J. , Pritchard, M. , Salas, A. & Singh, P. (2014) The ligamentum teres—its increasing importance. Journal of Hip Preservation Surgery, 1(1), 3–11. Available from: 10.1093/jhps/hnu003 27011796 PMC4765261

[joa70140-bib-0063] Parsi‐Pour, P. & Kilbourne, B. (2020) Functional morphology and morphological diversification of hind limb cross‐sectional traits in mustelid mammals. Integrative Organismal Biology, 2(1), obz032. Available from: 10.1093/iob/obz032 33791583 PMC7671153

[joa70140-bib-0064] Pearse, W. , Davies, T. & Wolkovich, E. (2025) How to define, use, and interpret Pagel's (lambda) in ecology and evolution. Global Ecology and Biogeography, 34, e70012. Available from: 10.1111/geb.70012

[joa70140-bib-0065] Pintore, R. , Houssaye, A. , Nesbitt, S. & Hutchinson, J. (2021) Femoral specializations to locomotor habits in early archosauriforms. Journal of Anatomy, 240, 867–892. Available from: 10.1111/joa.13598 34841511 PMC9005686

[joa70140-bib-0066] Quemeneur, S. , Buffrénil, V. & Laurin, M. (2013) Microanatomy of the amniote femur and inference of lifestyle in limbed vertebrates. Biological Journal of the Linnean Society, 109(3), 644–655. Available from: 10.1111/bij.12066

[joa70140-bib-0069] Ruff, C. & Hayes, W. (1983) Cross‐sectional geometry of Pecos Pueblo femora and tibiae—a biomechanical investigation: I. Method and general patterns of variation. American Journal of Physical Anthropology, 60(3), 359–381. Available from: 10.1002/ajpa.1330600308 6846510

[joa70140-bib-0070] Ruff, C. , Holt, B. & Trinkaus, E. (2006) Who's afraid of the big bad Wolff?: “Wolff's law” and bone functional adaptation. American Journal of Physical Anthropology, 129(4), 484–498. Available from: 10.1002/ajpa.20371 16425178

[joa70140-bib-0071] Ruimerman, R. , Hilbers, P. , Rietbergen (van), B. & Huiskes, R. (2005) A theoretical framework for strain‐related trabecular bone maintenance and adaptation. Journal of Biomechanics, 38(4), 931–941. Available from: 10.1016/j.jbiomech.2004.03.037 15713314

[joa70140-bib-0072] Ryan, T. & Ketcham, R. (2002) Femoral head trabecular bone structure in two omomyid primates. Journal of Human Evolution, 43(2), 241–263. Available from: 10.1006/jhev.2002.0575 12160718

[joa70140-bib-0074] Scheidt, A. , Wölfer, J. & Nyakatura, J. (2019) The evolution of femoral cross‐sectional properties in sciuromorph rodents: influence of body mass and locomotor ecology. Journal of Morphology, 280(8), 1156–1169. Available from: 10.1002/jmor.21007 31169943

[joa70140-bib-0075] Schindelin, J. , Arganda‐Carreras, I. , Frise, E. , Kaynig, V. , Longair, M. , Pietzsch, T. et al. (2012) Fiji: an open‐source platform for biological‐image analysis. Nature Methods, 9(7), 676–682. Available from: 10.1038/nmeth.2019 22743772 PMC3855844

[joa70140-bib-0076] Schmitt, D. (1998) Forelimb mechanics during arboreal and terrestrial quadrupedalism in old world monkeys. In: Strasser, E. , Fleagle, J. , Rosenberger, A. & McHenry, H. (Eds.) Primate locomotion: recent advances. Boston, MA: Springer, pp. 175–200. Available from: 10.1007/978-1-4899-0092-0_11

[joa70140-bib-0077] Scott, K. (1985) Allometric trends and locomotor adaptations in the Bovidae. Bulletin of the American Museum of Natural History, 179, Article 2. Available from: https://digitallibrary.amnh.org/handle/2246/1005 [Accessed 10th February 2022].

[joa70140-bib-0078] Scott, R. & Barr, A. (2014) Ecomorphology and phylogenetic risk: implications for habitat reconstruction using fossil bovids. Journal of Human Evolution, 73, 47–57. Available from: 10.1016/j.jhevol.2014.02.023 25038957

[joa70140-bib-0079] Seilacher, A. (1991) Self‐organizing mechanisms in morphogenesis and evolution. In: Schmidt‐Kittler, N. & Vogel, K. (Eds.) Constructional morphology and evolution. Berlin, Heidelberg: Springer, pp. 251–271. Available from: 10.1007/978-3-642-76156-0_17

[joa70140-bib-0080] Shaw, C. & Stock, J. (2011) The influence of body proportions on femoral and tibial midshaft shape in hunter‐gatherers. American Journal of Physical Anthropology, 144(1), 22–29. Available from: 10.1002/ajpa.21363 20623683

[joa70140-bib-0081] Stein, B. & Casinos, A. (1997) What is a cursorial mammal? Journal of Zoology, 242(1), 185–192. Available from: 10.1111/j.1469-7998.1997.tb02939.x

[joa70140-bib-0082] Takeda, S. , Oshida, T. , Motokawa, M. , Kawada, S.‐I. & Endo, H. (2023) Morphology of metapodiophalangeal joints and mobility of finger and toe in bovids. Mammal Study, 48(3), 145–157. Available from: 10.3106/ms2022-0049

[joa70140-bib-0083] Turner, C. (1998) Three rules for bone adaptation to mechanical stimuli. Bone, 23(5), 399–407. Available from: 10.1016/S8756-3282(98)00118-5 9823445

[joa70140-bib-0084] Upham, N. , Esselstyn, J. & Jetz, W. (2019) Inferring the mammal tree: species‐level sets of phylogenies for questions in ecology, evolution, and conservation. PLoS Biology, 17(12), e3000494. Available from: 10.1371/journal.pbio.3000494 31800571 PMC6892540

[joa70140-bib-0085] Wolff, J. (1986) Concept of the law of bone remodelling. In: Wolff, J. (Ed.) The law of bone remodelling. Berlin, Heidelberg: Springer, pp. 1–2. Available from: 10.1007/978-3-642-71031-5_1

