# Supplementary material for: Microanatomical features of bovids long bones: What are the effects of mass and habitat?
Source: J Anat. 2026 Apr 23:10.1111/joa.70140. Online ahead of print. doi: 10.1111/joa.70140 (PMC13398651; doi:10.1111/joa.70140)
Supplement: Supplementary file 1 — Appendix S1. [file JOA-9999-0-s001.zip › S2_lm_table_post_hoc_nemeniy.docx]

*Table S2.1: results of the linear regression of the microanatomical parameters and first two axis of the PCA with the WBV*

|  | Parameters | P-value | Mean R^2^ |
| --- | --- | --- | --- |
| Humerus | Mass | **5.95e-10** | **0.97** |
|  | Bone compactness | 0.91 | <0.05 |
|  | Trabecular compactness | 0.13 | 0.12 |
|  | Relative fraction of trabecular bone | 0.08 | 0.25 |
|  | Relative mean compact thickness | 0.14 | 0.18 |
|  | Relative maximal compact thickness | 0.63 | 0.02 |
|  | PC1 | 0.47 | -0.04 |
|  | PC2 | 0.06 | 0.22 |
| Radius-ulna | Mass | **1.46e-8** | **0.95** |
|  | Bone compactness | 0.82 | <0.05 |
|  | Trabecular compactness | **0.02** | **0.41** |
|  | Relative fraction of trabecular bone | **<0.01** | **0.62** |
|  | Relative mean compact thickness | 0.1 | 0.25 |
|  | Relative maximal compact thickness | **0.02** | **0.41** |
|  | PC1 | **0.01** | **0.41** |
|  | PC2 | 0.19 | 0.08 |
| Femur | Mass | **5.16e-9** | **0.96** |
|  | Bone compactness | 0.59 | 0.03 |
|  | Trabecular compactness | **0.03** | **0.38** |
|  | Relative fraction of trabecular bone | **<0.05** | **0.32** |
|  | Relative mean compact thickness | 0.31 | 0.09 |
|  | Relative maximal compact thickness | 0.6 | 0.03 |
|  | PC1 | 0.78 | -0.08 |
|  | PC2 | **0.03** | **0.314** |
| Tibia | Mass | **1.15e-6** | **0.89** |
|  | Bone compactness | 0.99 | <0.01 |
|  | Trabecular compactness | **0.03** | **0.37** |
|  | Relative fraction of trabecular bone | **<0.01** | **0.77** |
|  | Relative mean compact thickness | 0.16 | 0.17 |
|  | Relative maximal compact thickness | 0.82 | 0.01 |
|  | PC1 | 0.77 | -0.08 |
|  | PC2 | **<0.001** | **0.8** |


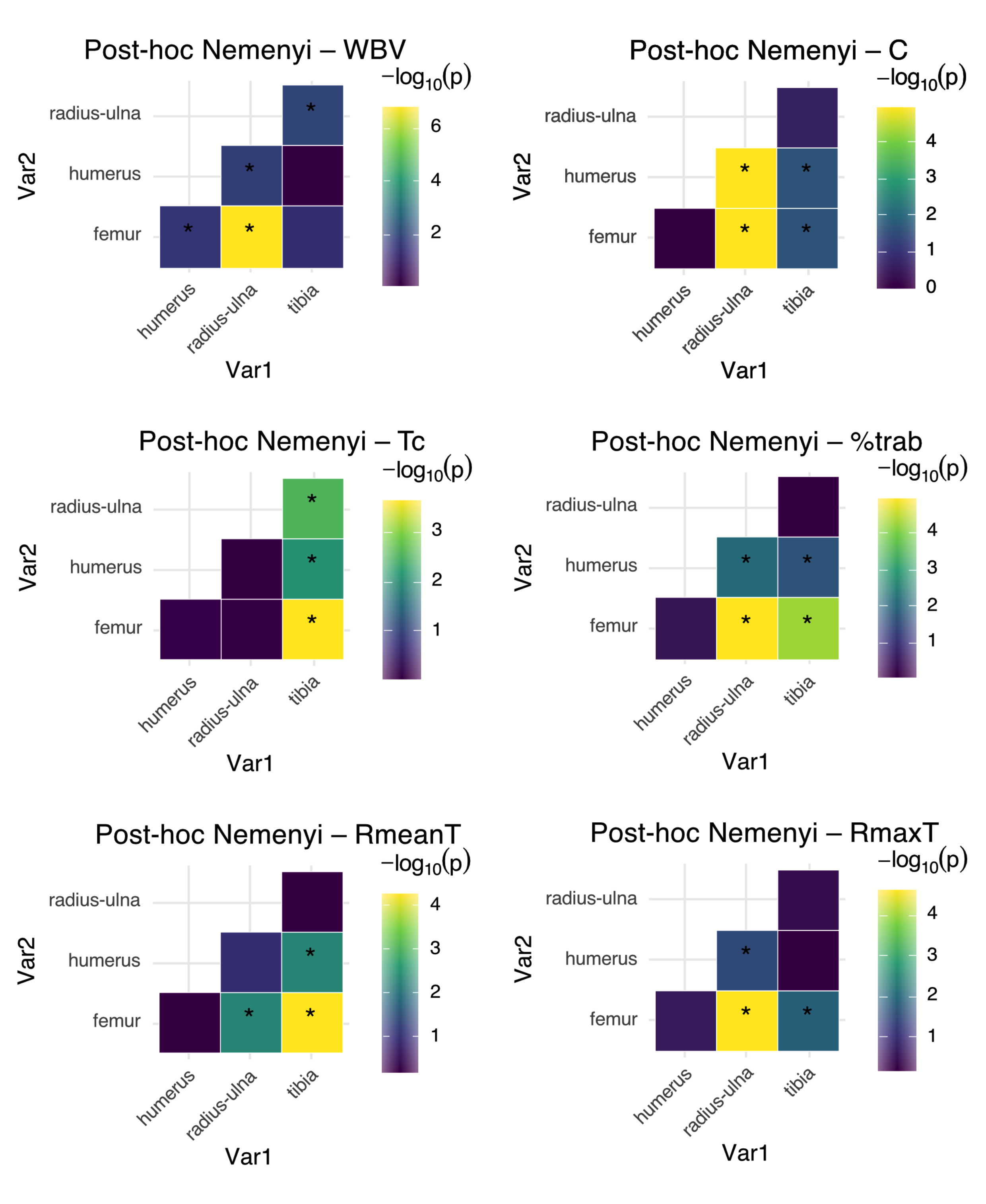


*Figure S2.2: Nemenyi’s post-hoc test following the Friedman test comparing the covariation between bones for WBV and each microanatomical parameter. The asterisk (*) mean a significant covariation.*
