# Supplementary material for: Microanatomical features of bovids long bones: What are the effects of mass and habitat?
Source: J Anat. 2026 Apr 23:10.1111/joa.70140. Online ahead of print. doi: 10.1111/joa.70140 (PMC13398651; doi:10.1111/joa.70140)
Supplement: Supplementary file 1 — Appendix S1. [file JOA-9999-0-s001.zip › S6_PCA.docx]

Principal component analyses descriptions

The first axis of the PCA explains between 50.5% (femur) and 66.3% (tibia) of total variance. while the second axis of the PCA explains between 29.4% (radius-ulna) and 39.0% (femur) of variance. They thus enable us to document much of the total variance for each bone. The contributions of the microanatomical parameters to PC1 and PC2, observed on the PCA correlation circle (variable loading plot), are rather similar between the bones. The first axis is essentially driven by the relative maximal and mean compact thicknesses (RmaxT and RmeanT). Trabecular compactness (Tc) and global compactness (C) seem to always positively covary and contribute equally to PC1 and PC2, except for the tibia for which they seem to slightly contribute more to the first axis. These two parameters (Tc and C) also covary negatively with the relative fraction of the trabecular bone (%trab) on the second axis. All the more, similarities between the specimen distribution in the morphospaces appear greater for stylopod bones (Figure S1.A; 9.C) with species living in an open environment being mostly on the negative side of the first axis, while species living in a hard cover or mountainous environment being mostly on the positive side of the first axis. Moreover, *C. gnou*, living in an open habit, always occurs on the positive side with species living in a hard cover or mountainous environment. The heaviest specimens in the sample are always sorted along on one side of the second axis, while the lightest are on the other side, although differences can be seen between each bone (see below) .

Humerus (Figure S1. A). The first axis represents half of the variance (51.6%), while the second axis represents 33.9%. Neither PC1 nor PC2 are correlated with WBV (Table S2.2).

Radius-Ulna (Figure S1. B). The first axis represents two-thirds of the variance (62.1 %) and is correlated with mass (Table S2.2). Heaviest species are on the negative side and thus display a higher relative fraction of trabecular bone. The second axis represents 29.4% of the variance and sets appart open-habitat species from the rest, albeit with a great overlap.

Femur (Figure S1. C). The first and second axes represents half of the variance (50.5 %), and 39.0%, respectively. The second axis is positively correlated with WBV (Table S2.2).

Tibia (Figure S1. D). The first axis represents two-thirds of the variance (66.3 %), and the second one 31.6%. The second axis is positively correlated with WBV (Table S2.2).


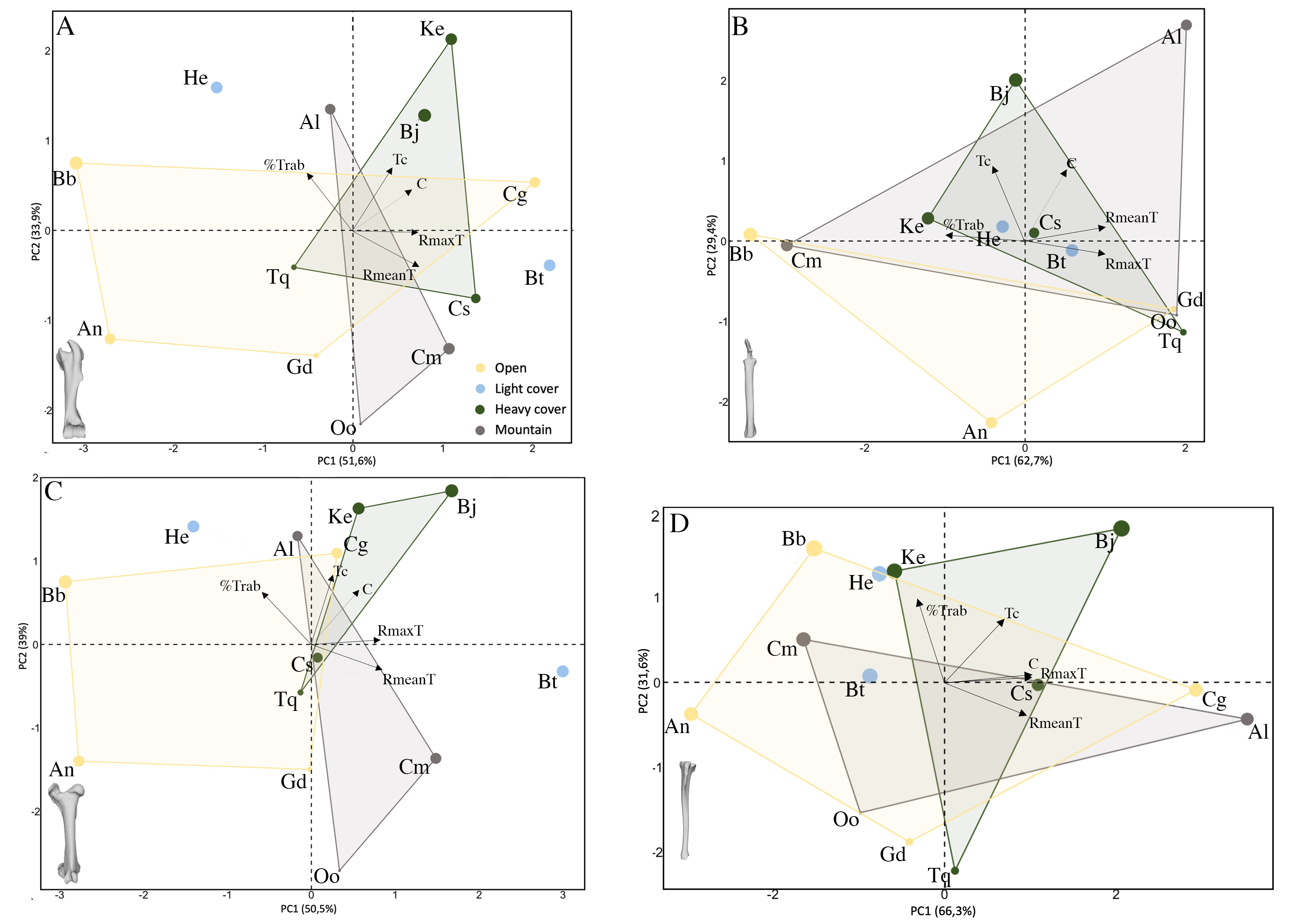


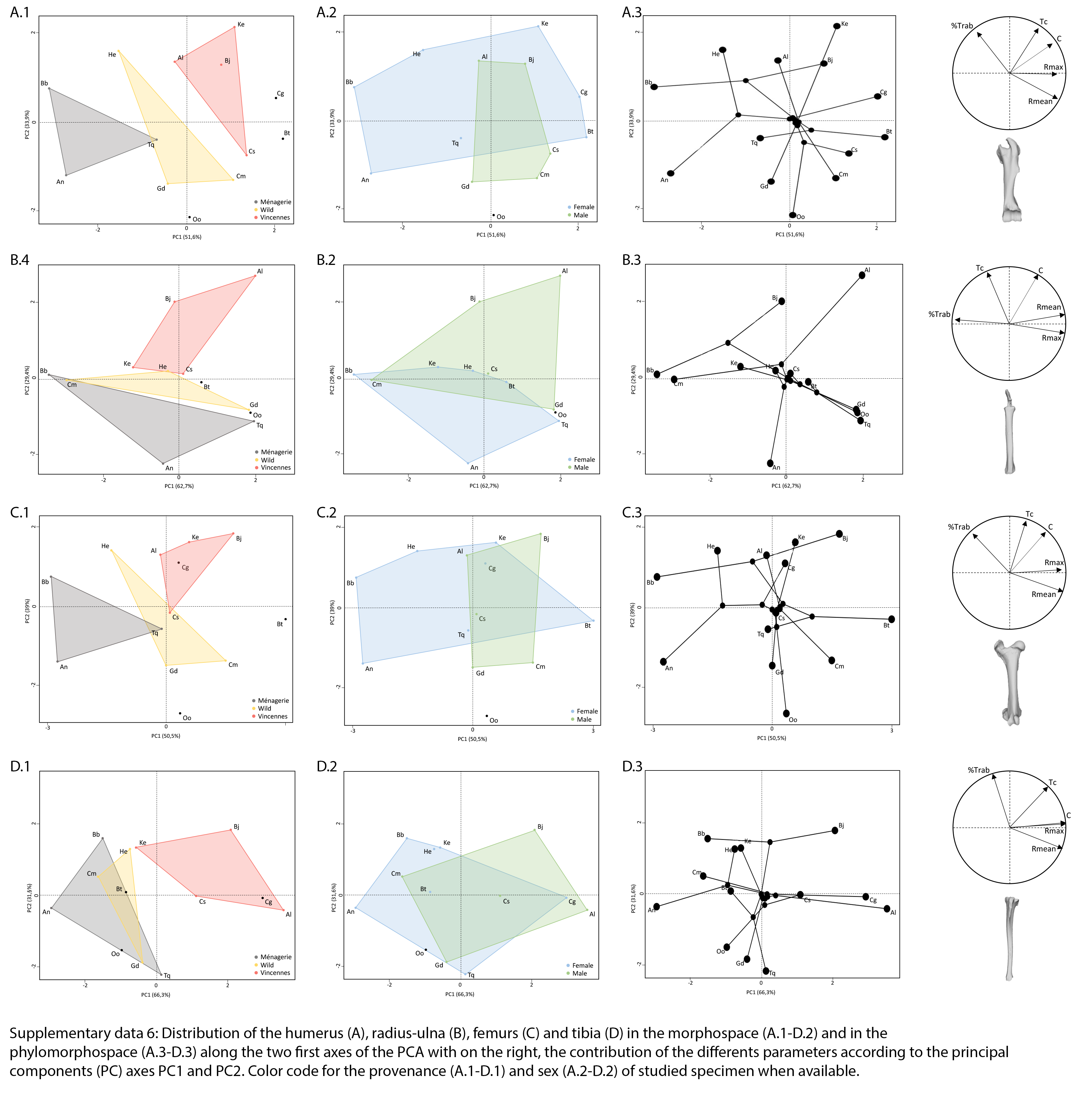


Figure S1. A: PCA results for the humerus (A), radius-ulna (B), femur (C), tibia (D), showing the first two components with the contribution of the different microanatomical parameters to the PCs. Point color is according to habitat; point size is proportional to the logarithm (log) of the whole bone volume. The correlation circle shows the contribution of the microanatomical parameters to PC1 and PC2. The underlying graph shows the first two components with point color according to captivity (X.1), sex (X.2), and the phylomorphospace (X.3) for each bone (A: humerus; B: radius-ulna; C: femur; D: tibia). For captivity, this sample encounters three main modes, 1) Menagerie, which is an historical zoo where the animal well-being was not the main concern (small space, not adapted food, mostly concrete as a substrate, and no predatorial pressures), 2) Wild, correspond to specimens in our sample living their entire life free of zoo constraints (living in their natural environment with the food within, but with predatorial pressure), and 3) Vincennes, correspond to a more modern zoo in which the animal well-being is considered (more place which mimics a natural environment to roam, adapted food, but no predatorial pressure).
