# Supplementary material for: Microanatomical features of bovids long bones: What are the effects of mass and habitat?
Source: J Anat. 2026 Apr 23:10.1111/joa.70140. Online ahead of print. doi: 10.1111/joa.70140 (PMC13398651; doi:10.1111/joa.70140)
Supplement: Supplementary file 1 — Appendix S1. [file JOA-9999-0-s001.zip › S5_thickness_maps.zip!/Supdata5_thickness_maps/Supdata5_1_Humerus.pdf]

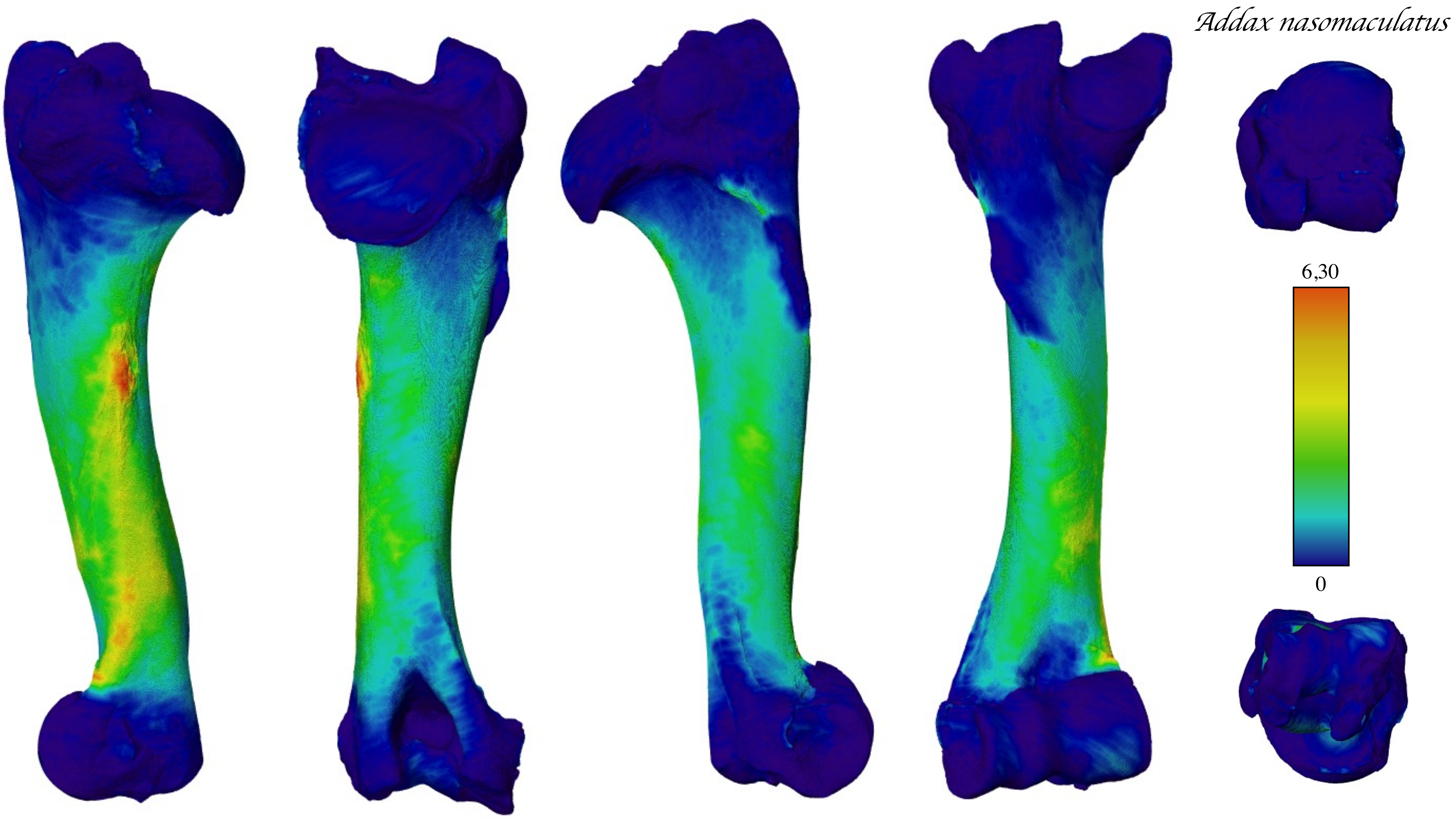

Addax nasomaculatus
6,30
0

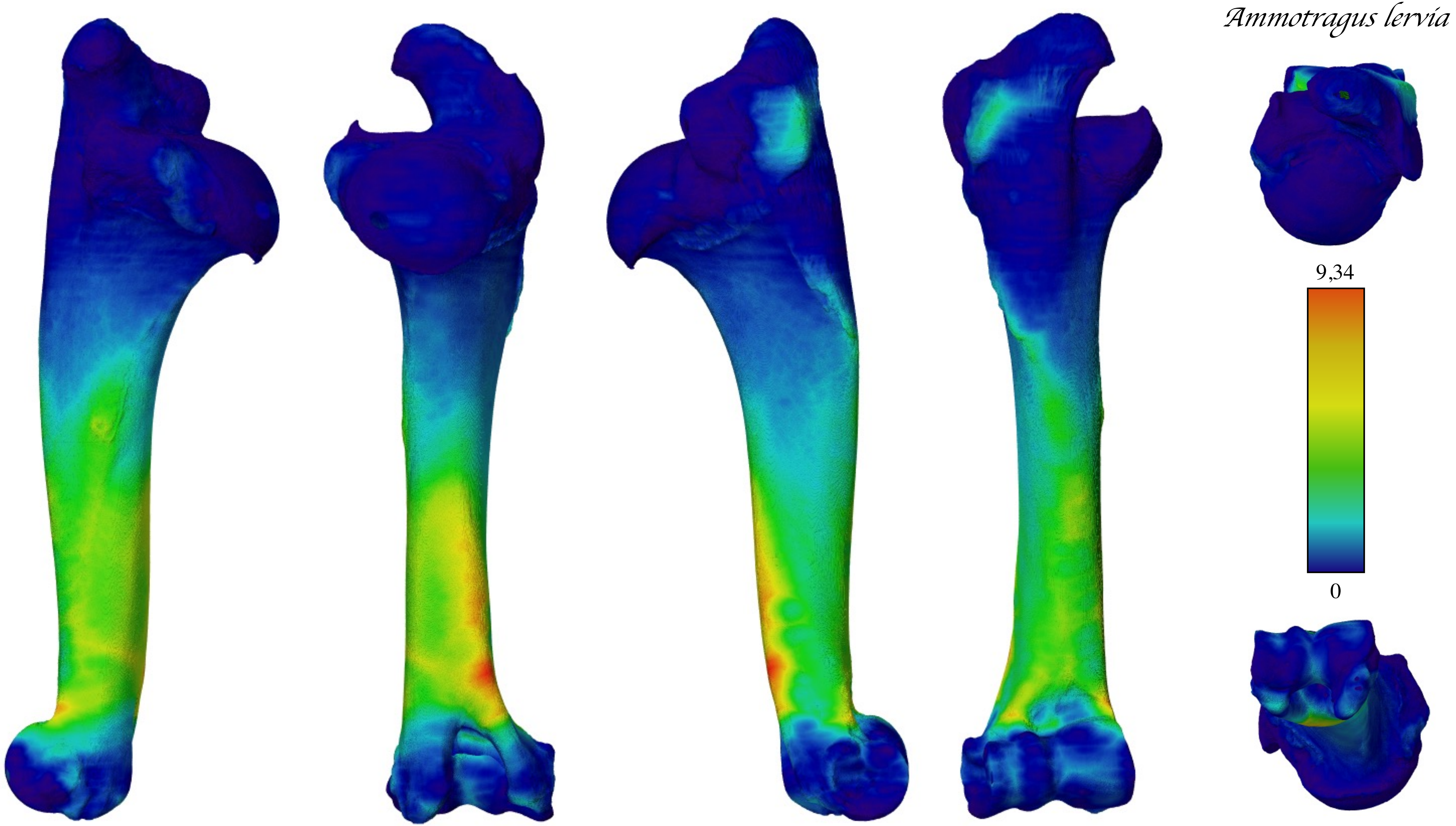

Ammotragus lervia
9,34
0

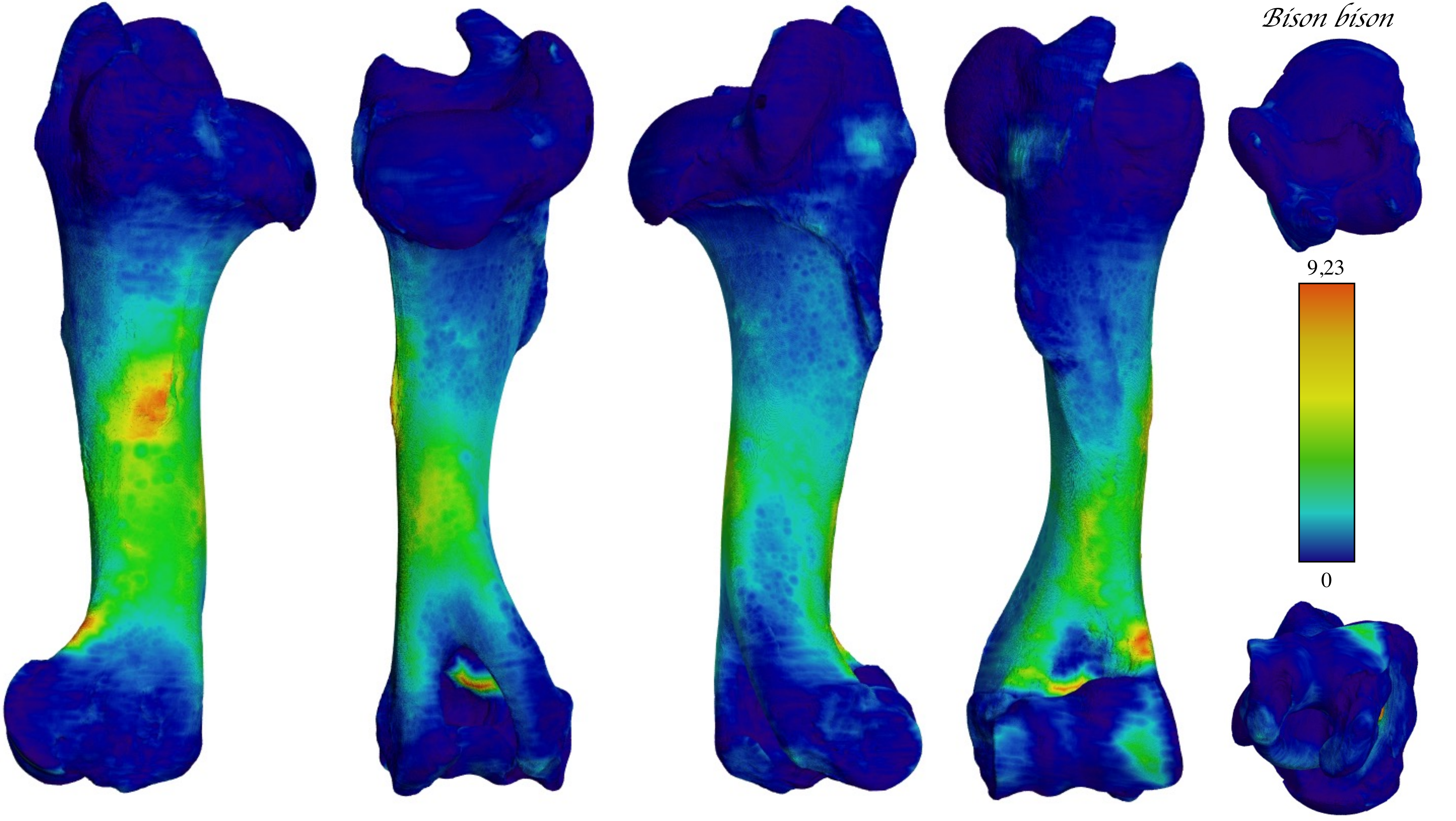
Bison bison
9,23
0

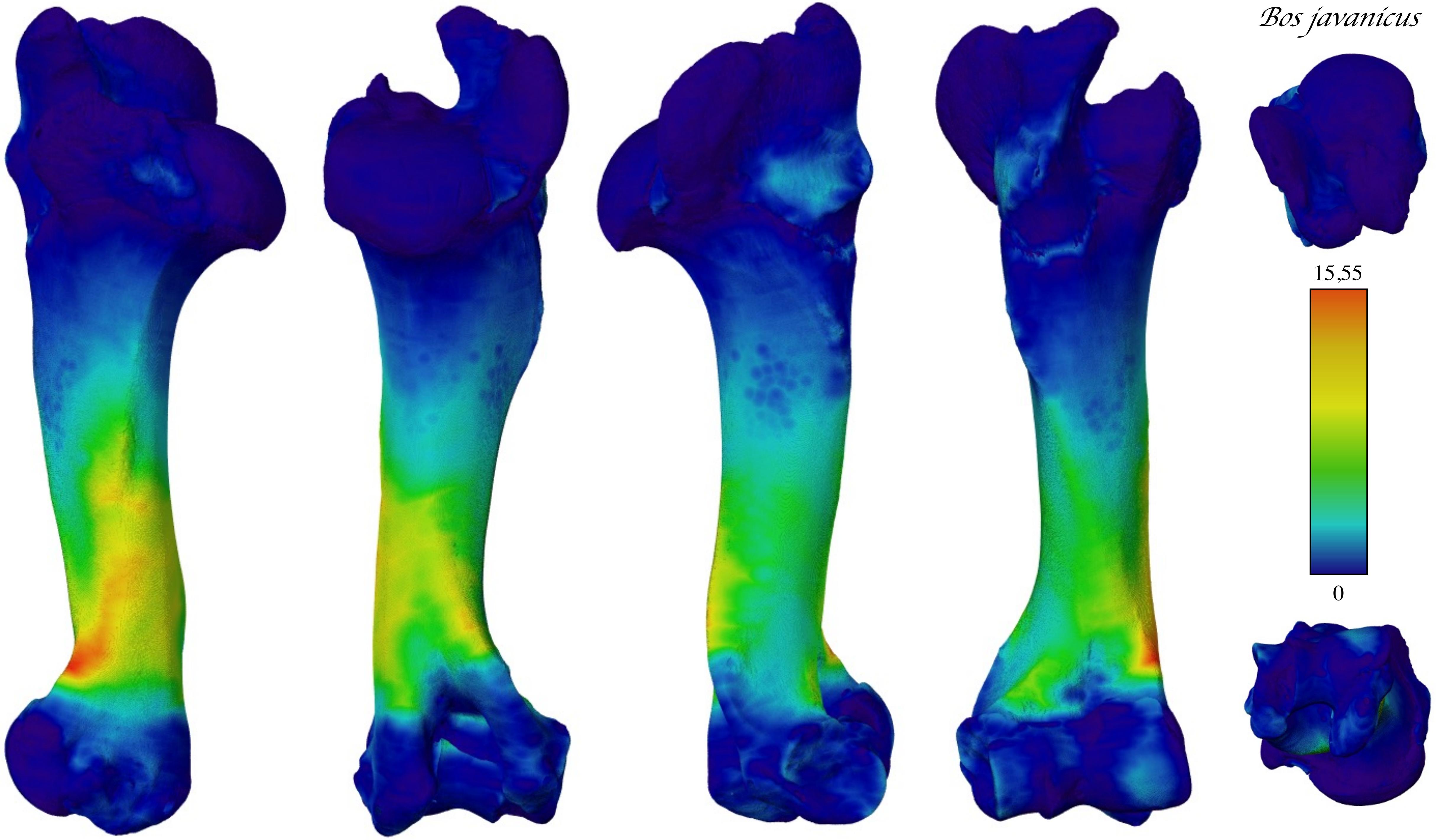

Bos javanicus
15,55
0

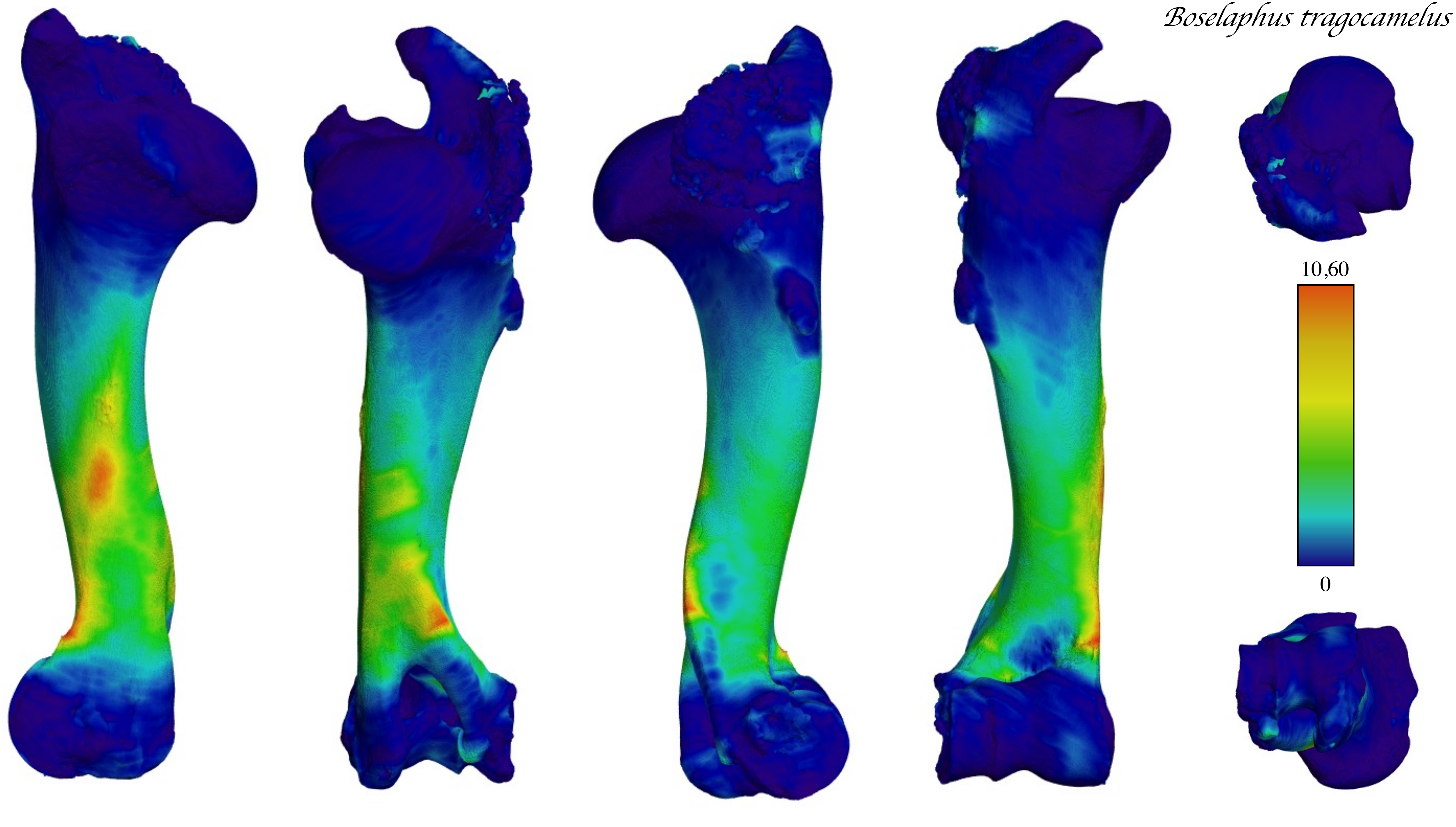
Boselaphus tragocamelus
10,60
0

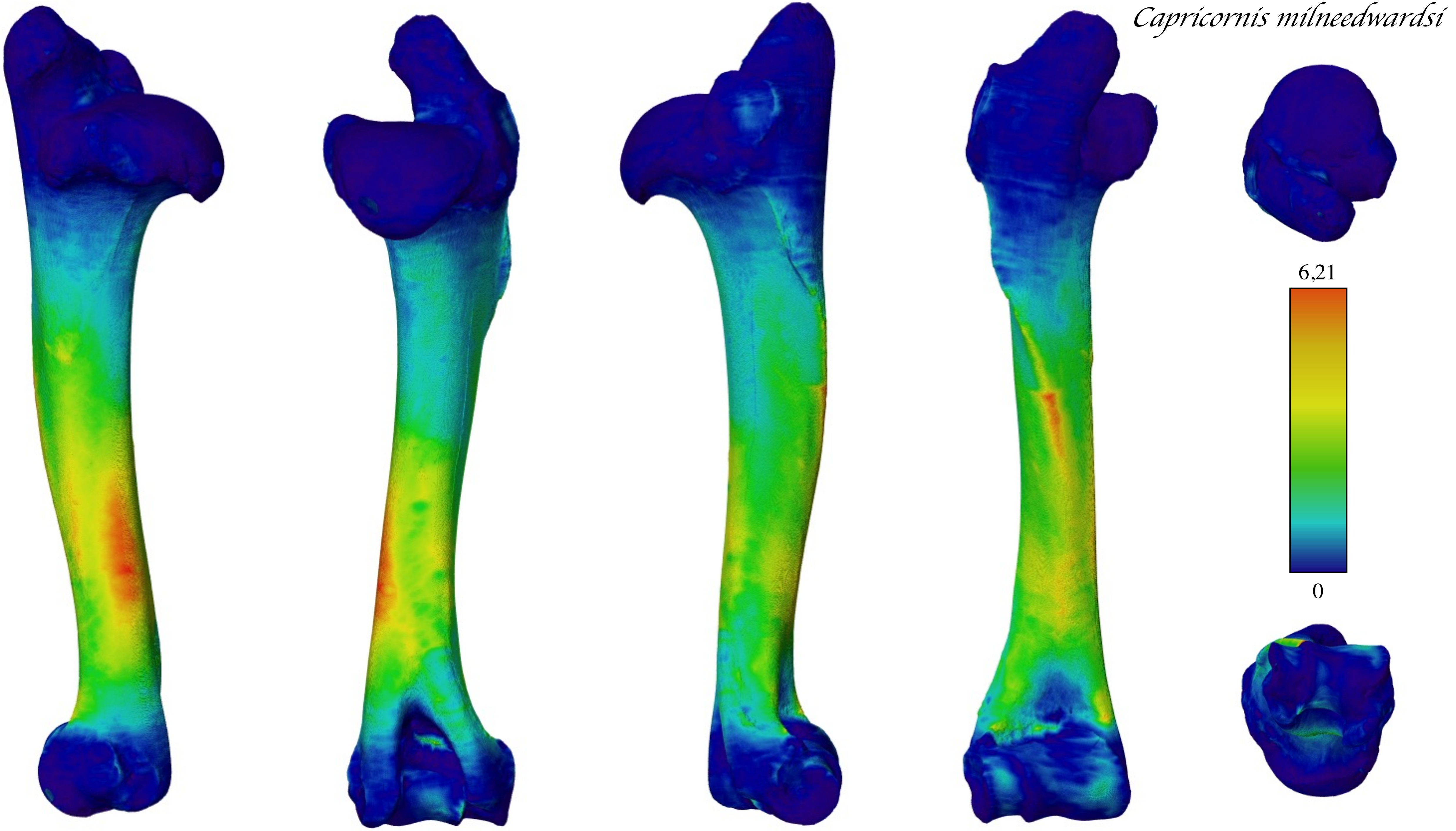
Capricornis milneedwardsi
6,21
0

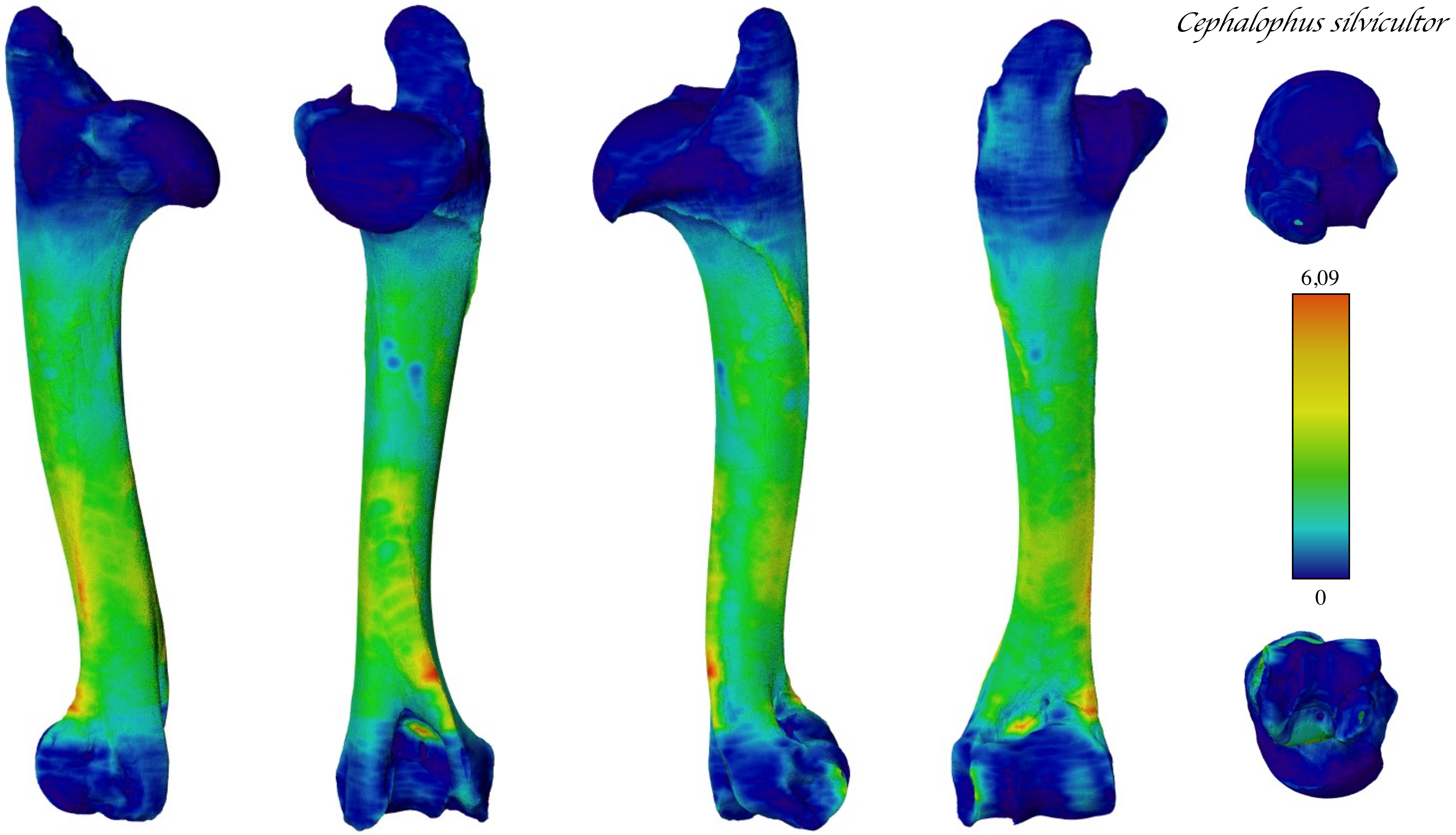
Cephalophus silvicultor
6,09
0

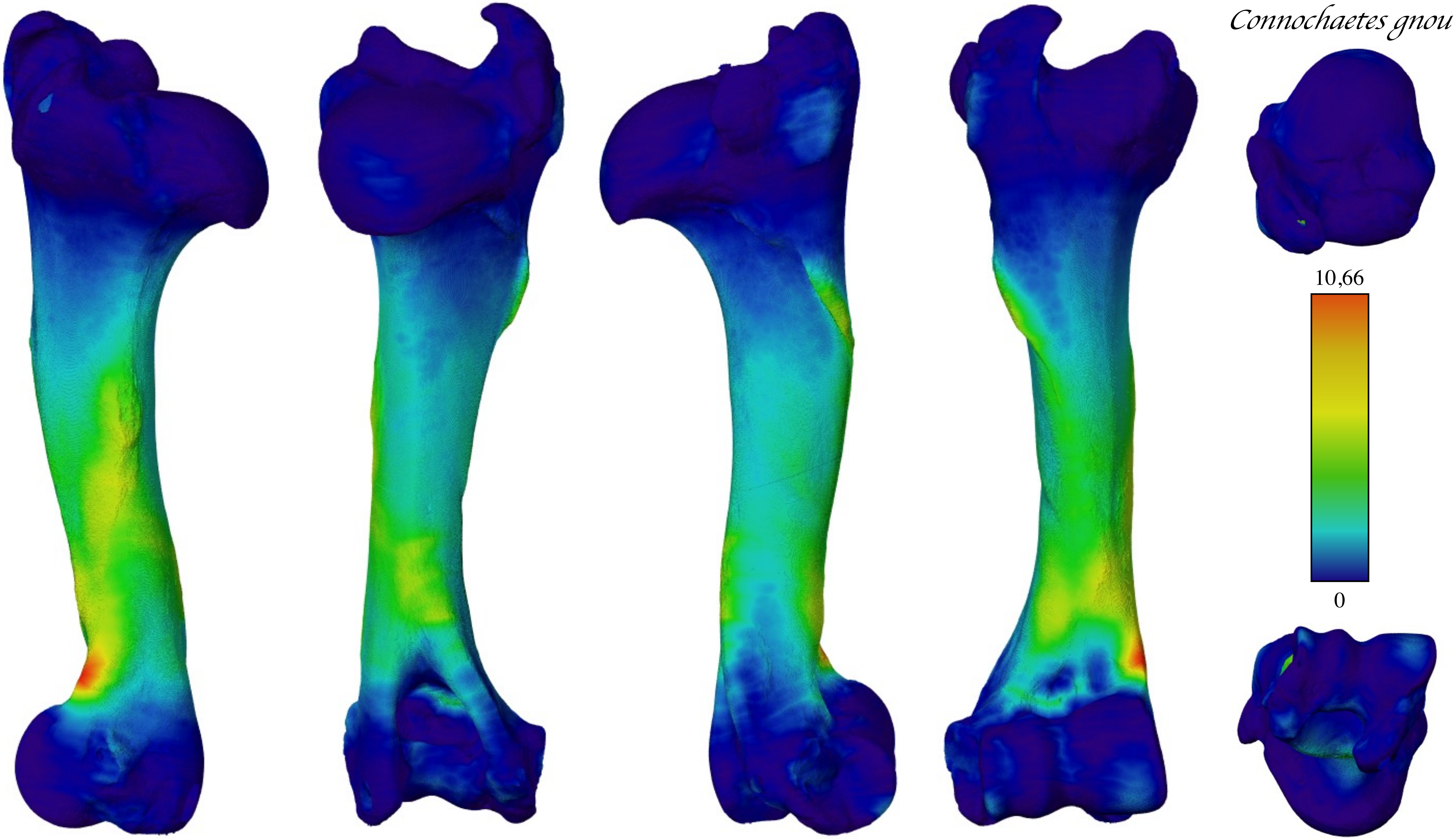
Connochaetes gnou
10,66
0

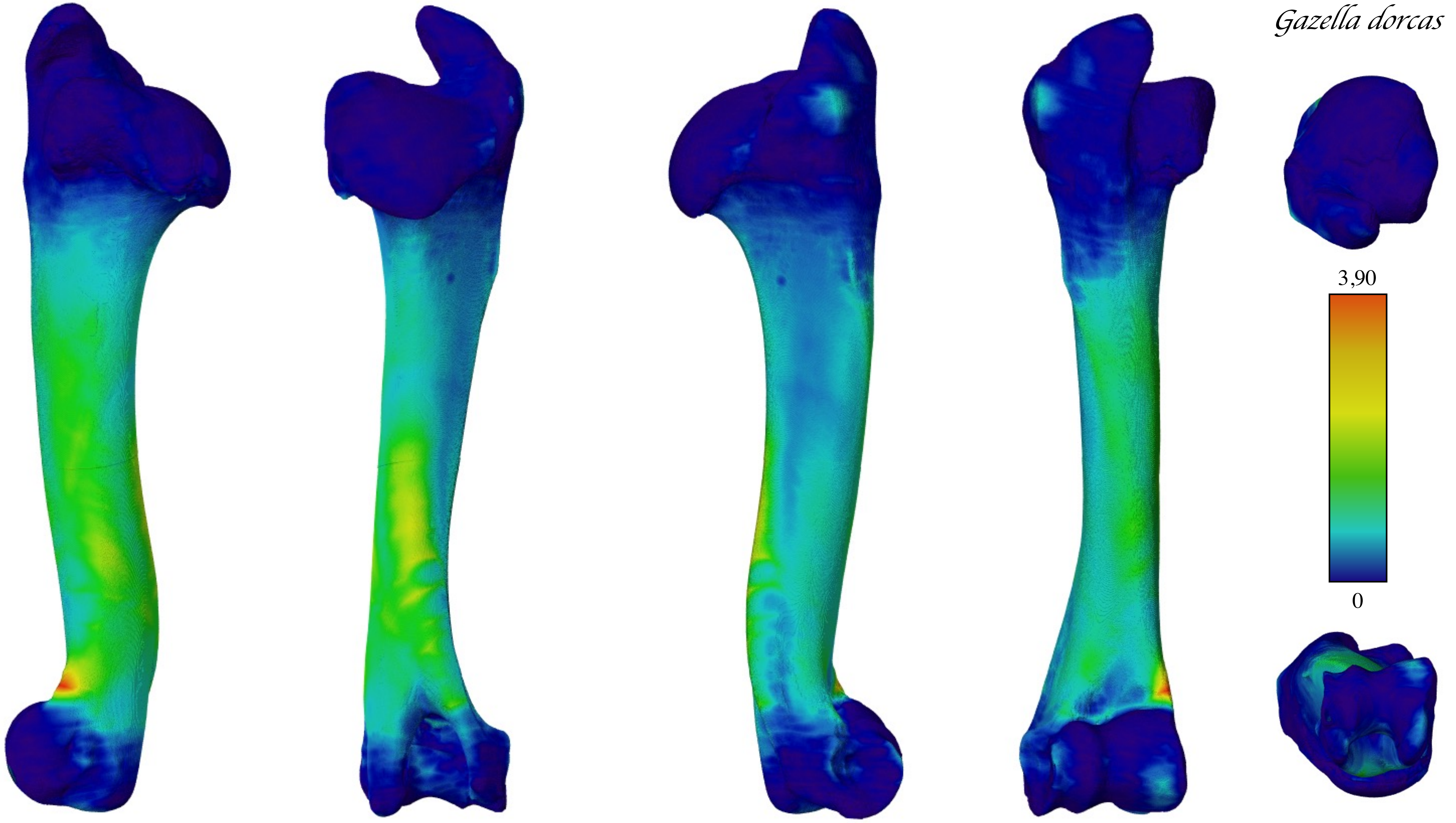

Gazella dorcas
3,90
0

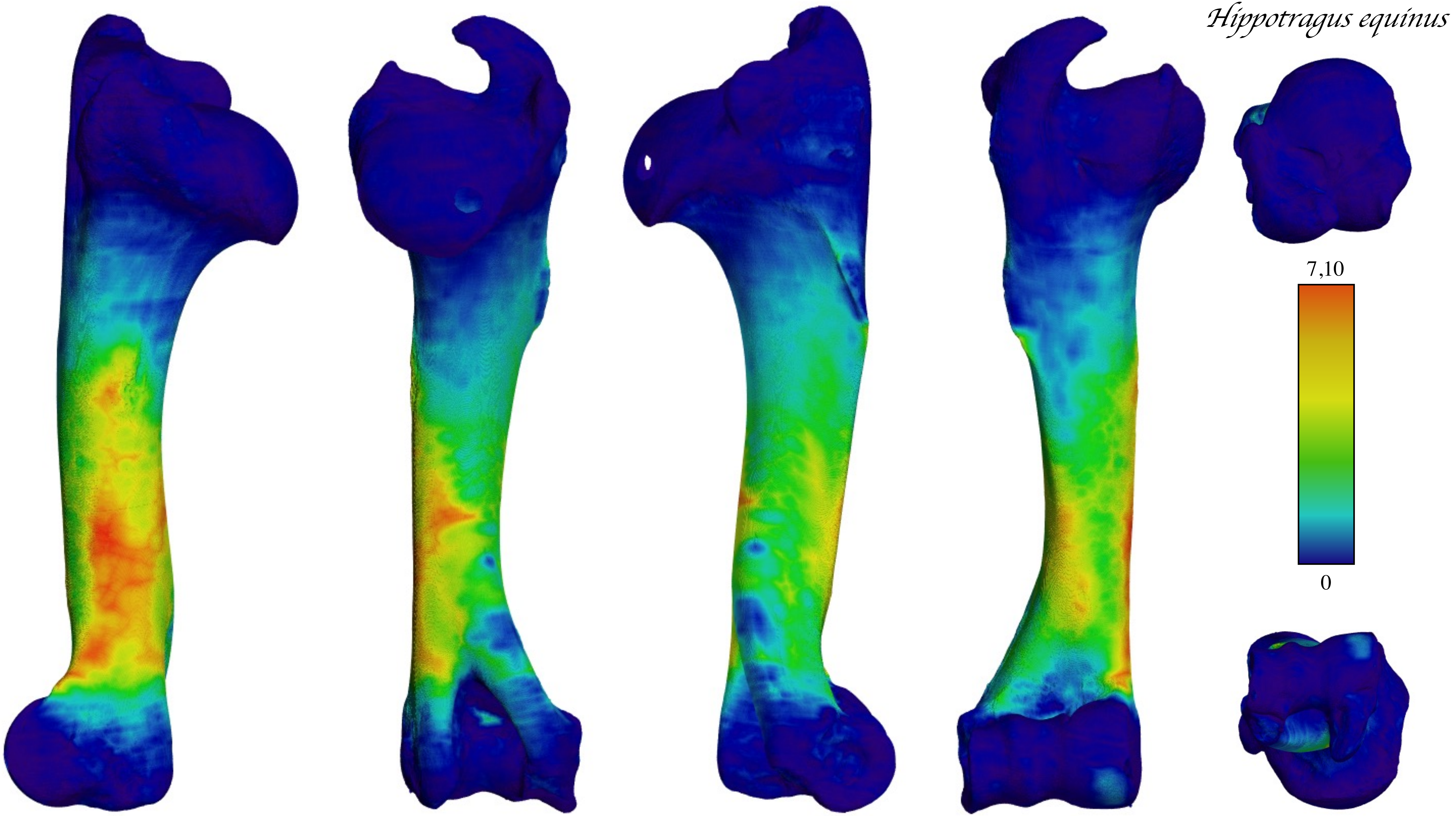

Hippotragus equinus
7,10
0

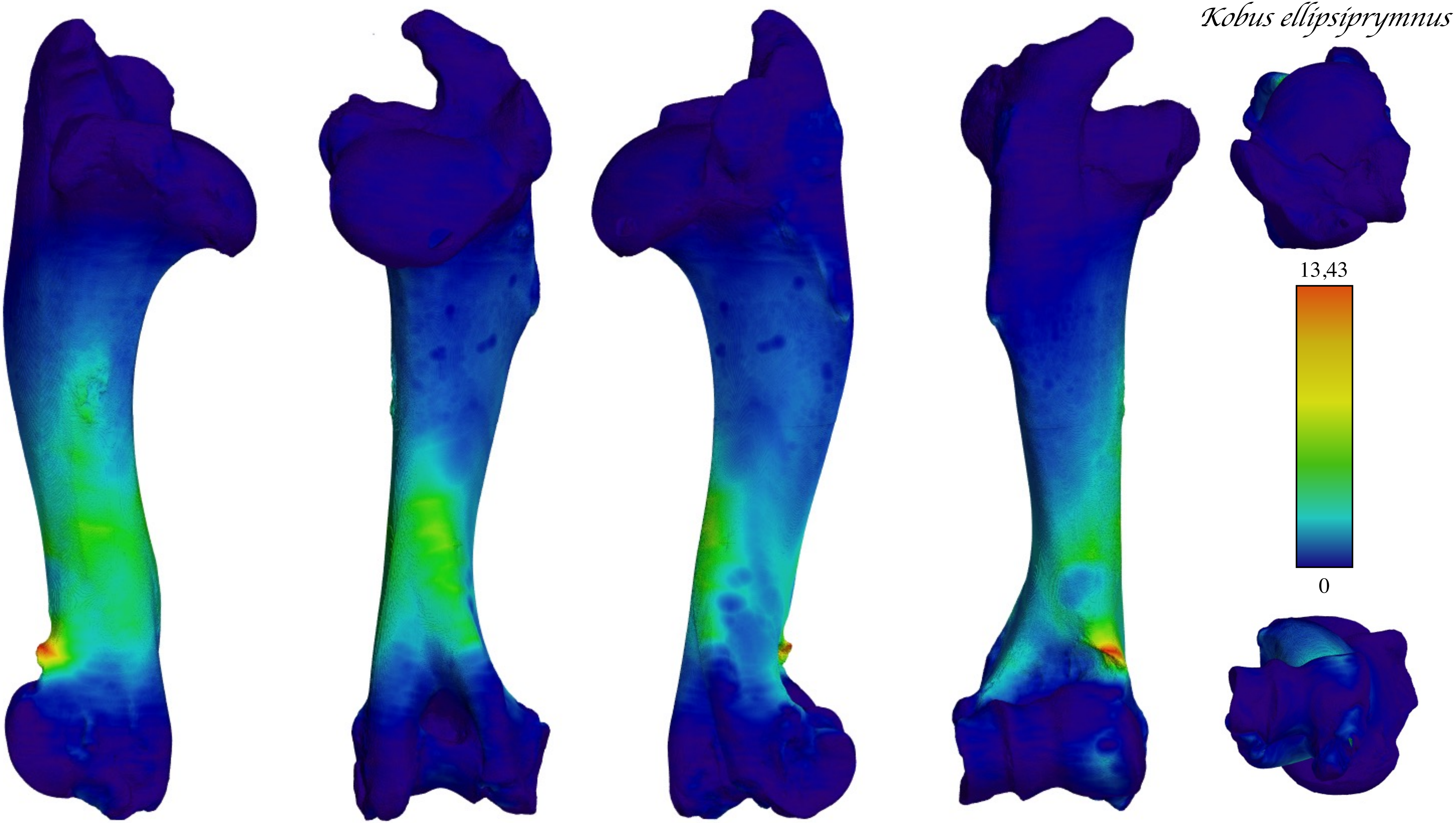
Kobus ellipsiprymnus
13,43
0

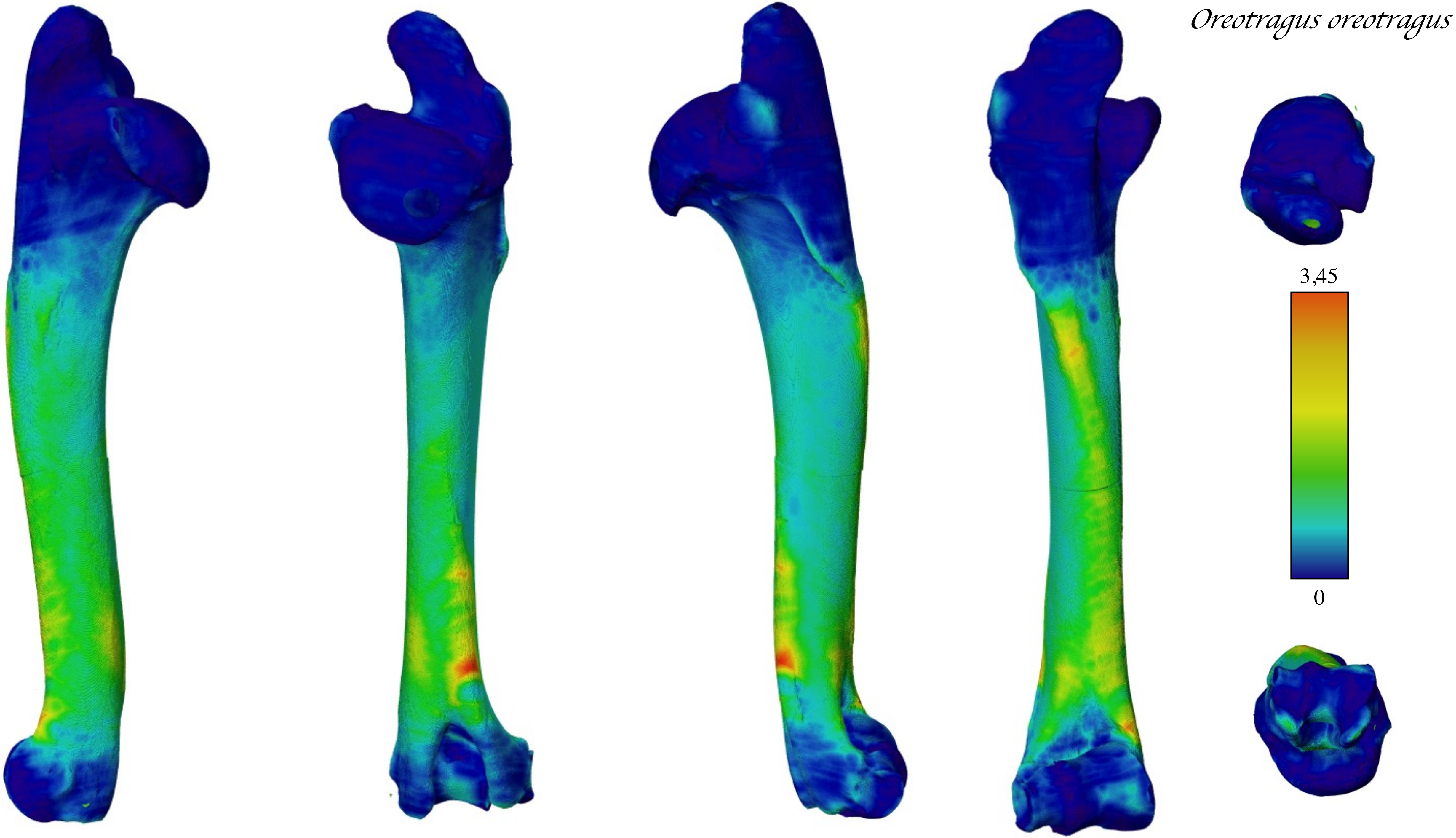
Oreotragus oreotragus
3,45
0

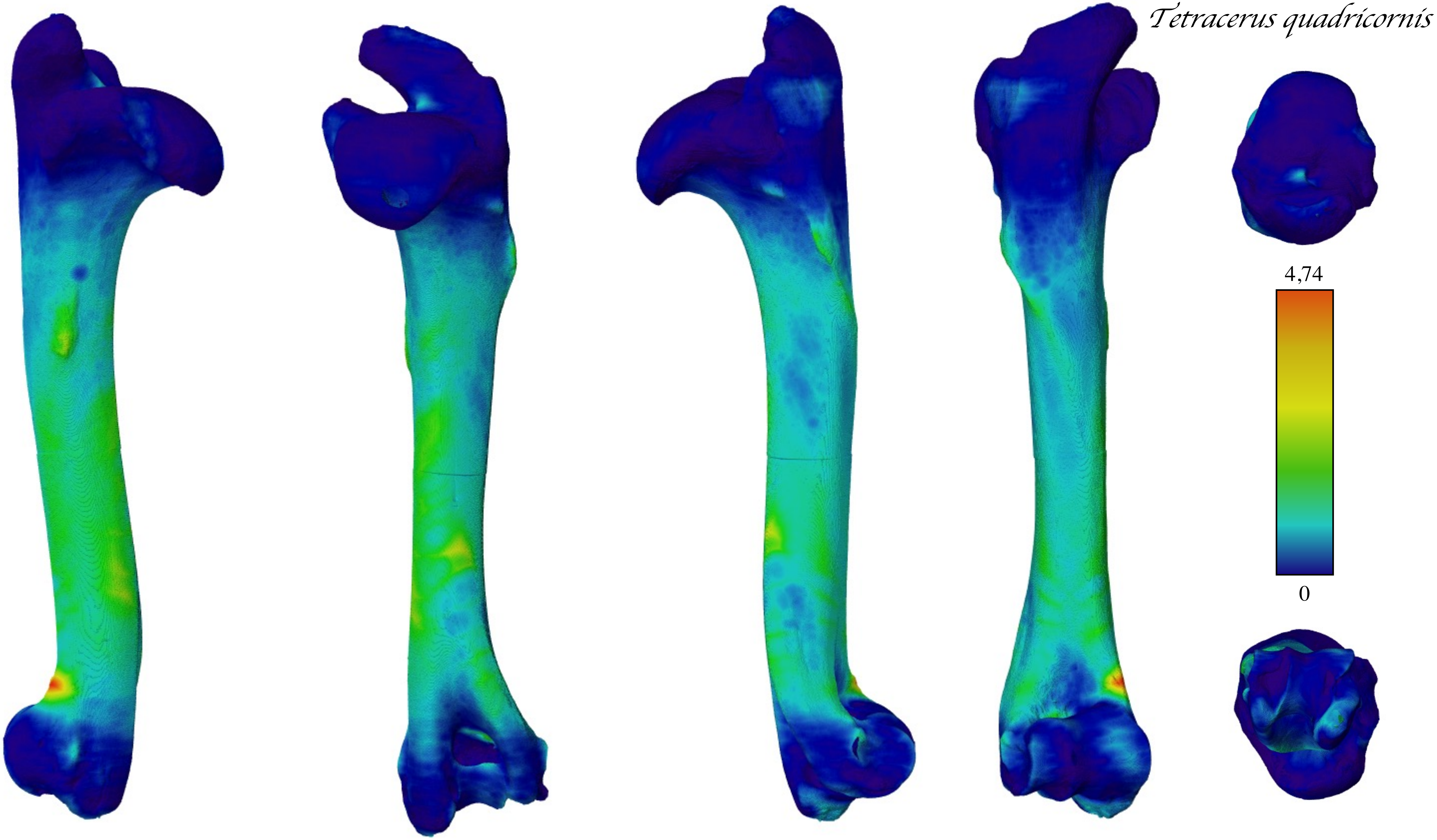

Tetracerus quadricornis
4,74
0
