# Supplementary material for: Microanatomical features of bovids long bones: What are the effects of mass and habitat?
Source: J Anat. 2026 Apr 23:10.1111/joa.70140. Online ahead of print. doi: 10.1111/joa.70140 (PMC13398651; doi:10.1111/joa.70140)
Supplement: Supplementary file 1 — Appendix S1. [file JOA-9999-0-s001.zip › S5_thickness_maps.zip!/Supdata5_thickness_maps/Supdata5_2_radius_ulna.pdf]

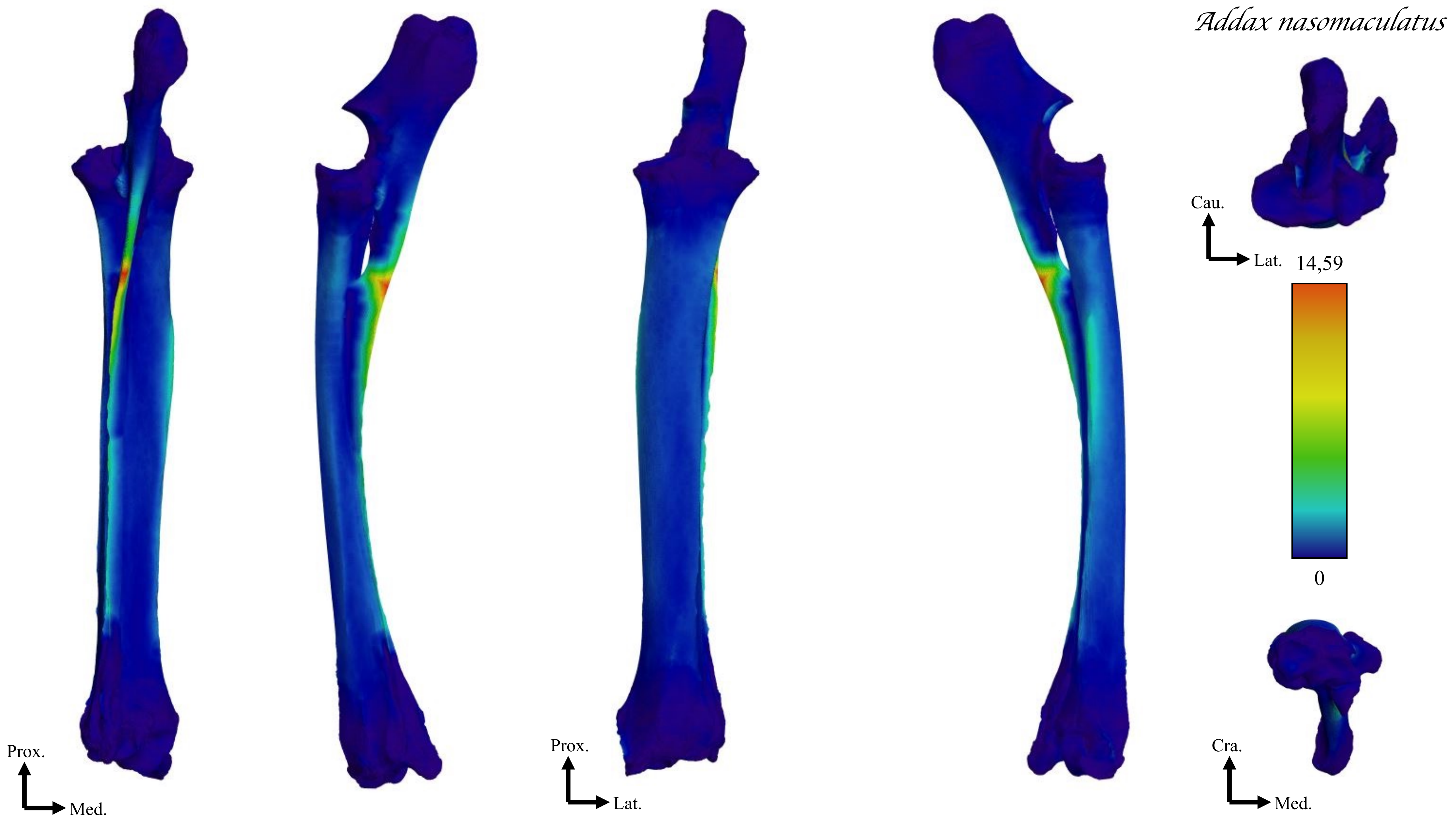
Addax nasomaculatus
Cau.
Lat.
14,59
0
Prox.
Med.
Prox.
Lat.
Cra.
Med.

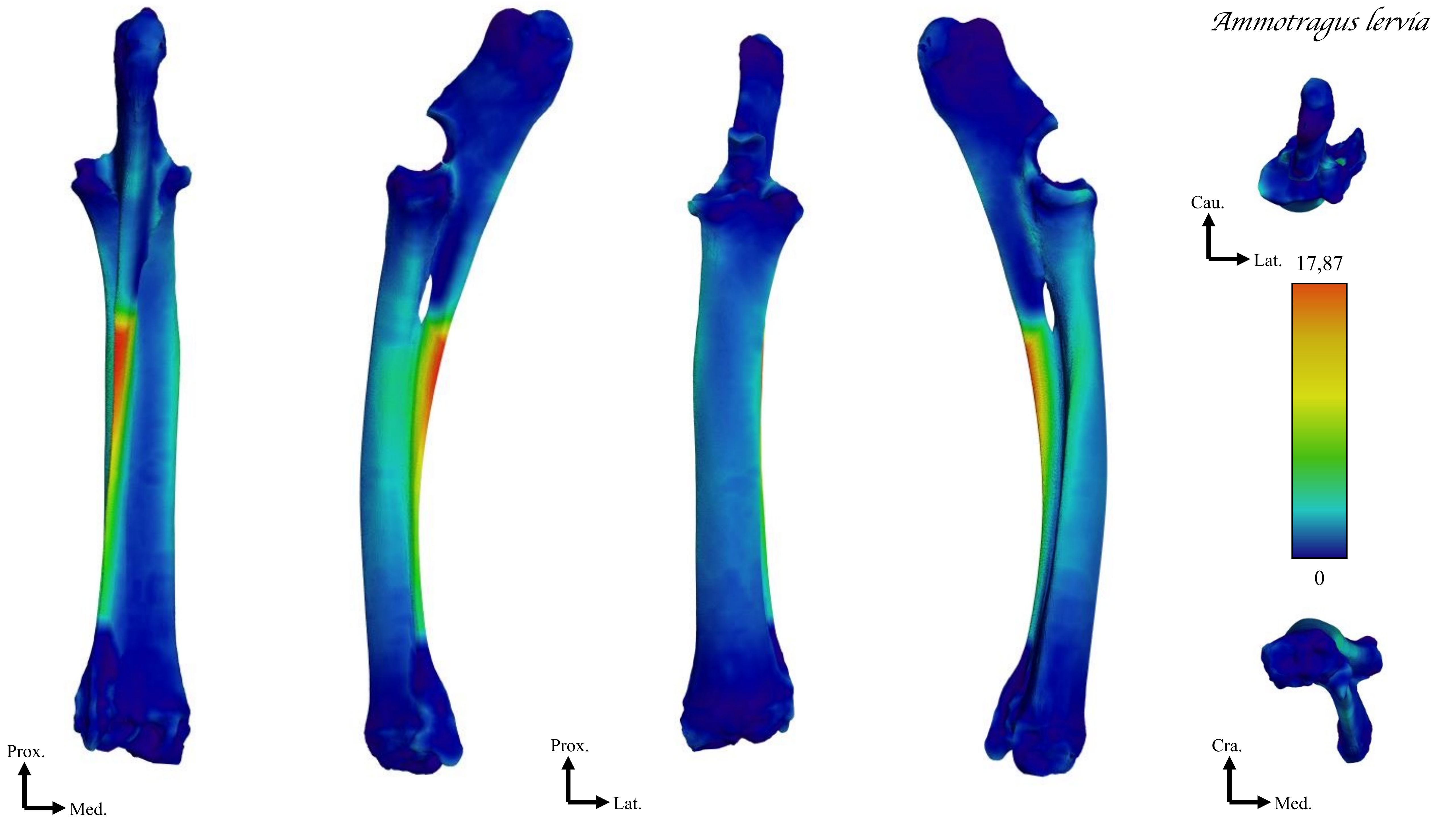
Ammotragus lervia
Cau.
Lat.
17,87
0
Prox.
Med.
Prox.
Lat.
Cra.
Med.

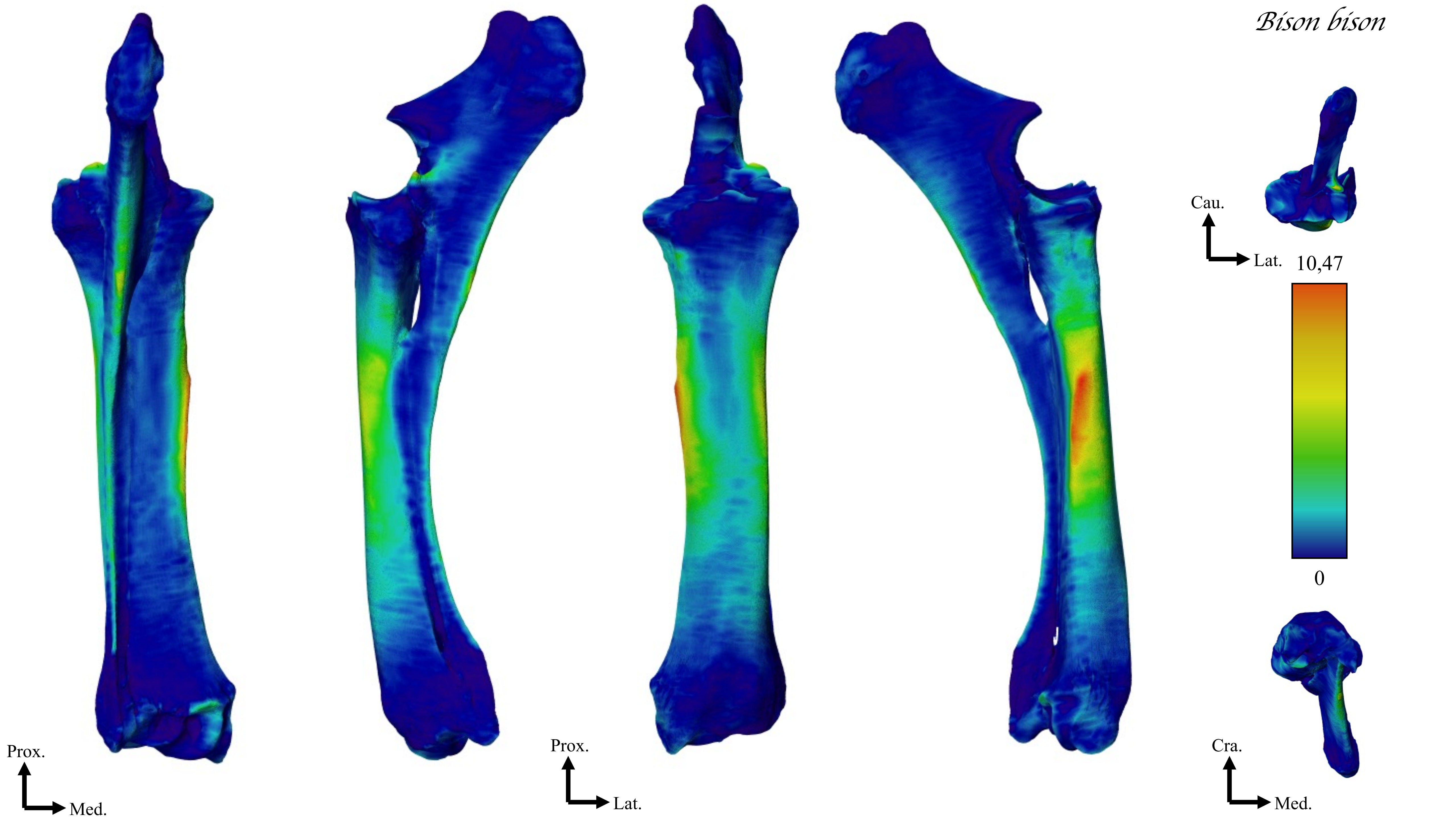

Bison bison
Cau.
Lat.
10,47
0
Prox.
Med.
Prox.
Lat.
Cra.
Med.

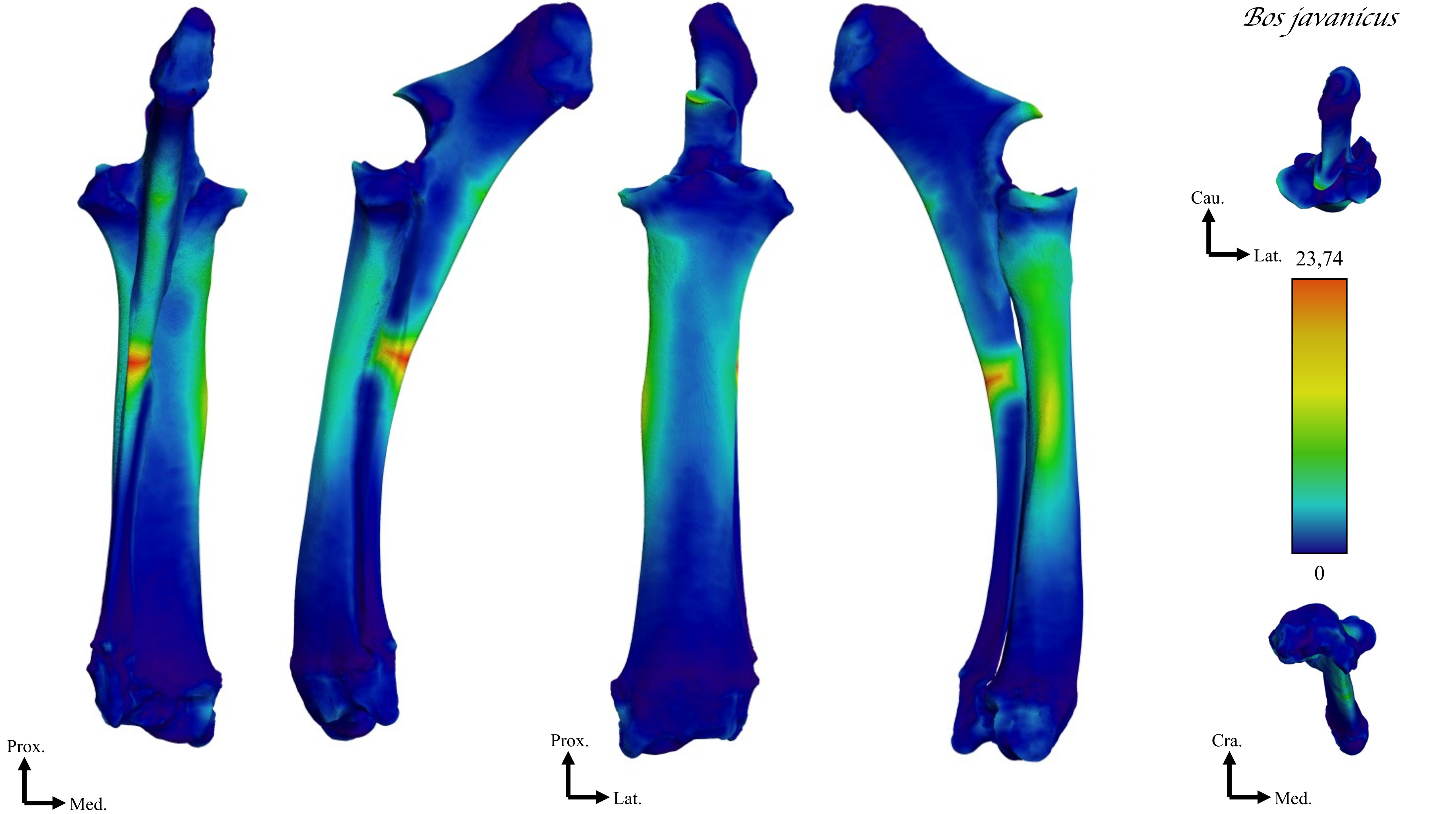

Bos javanicus
Cau.
Lat.
23,74
0
Prox.
Med.
Prox.
Lat.
Cra.
Med.

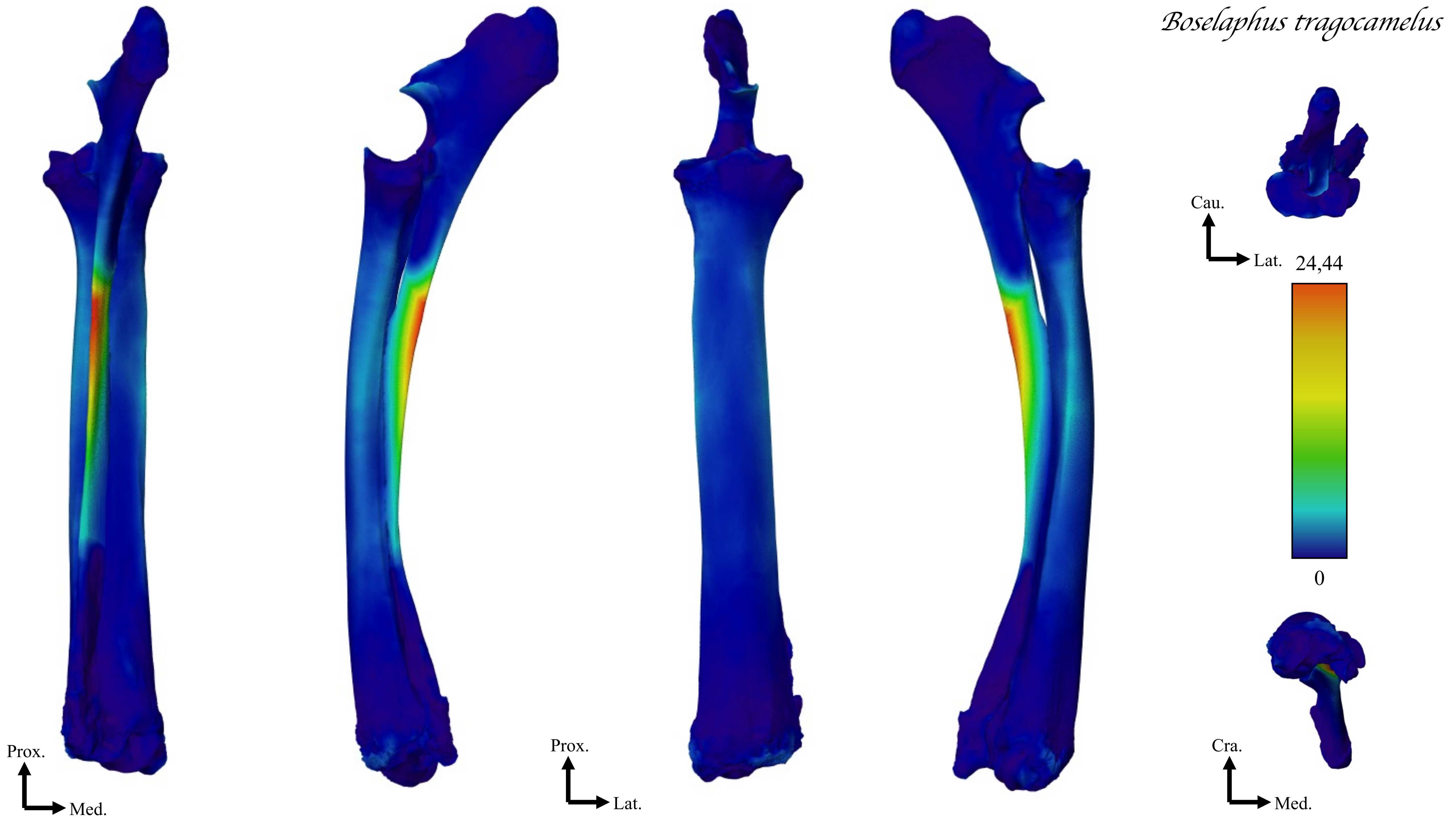

Boselaphus tragocamelus
Cau.
Lat.
24,44
0
Prox.
Med.
Prox.
Lat.
Cra.
Med.

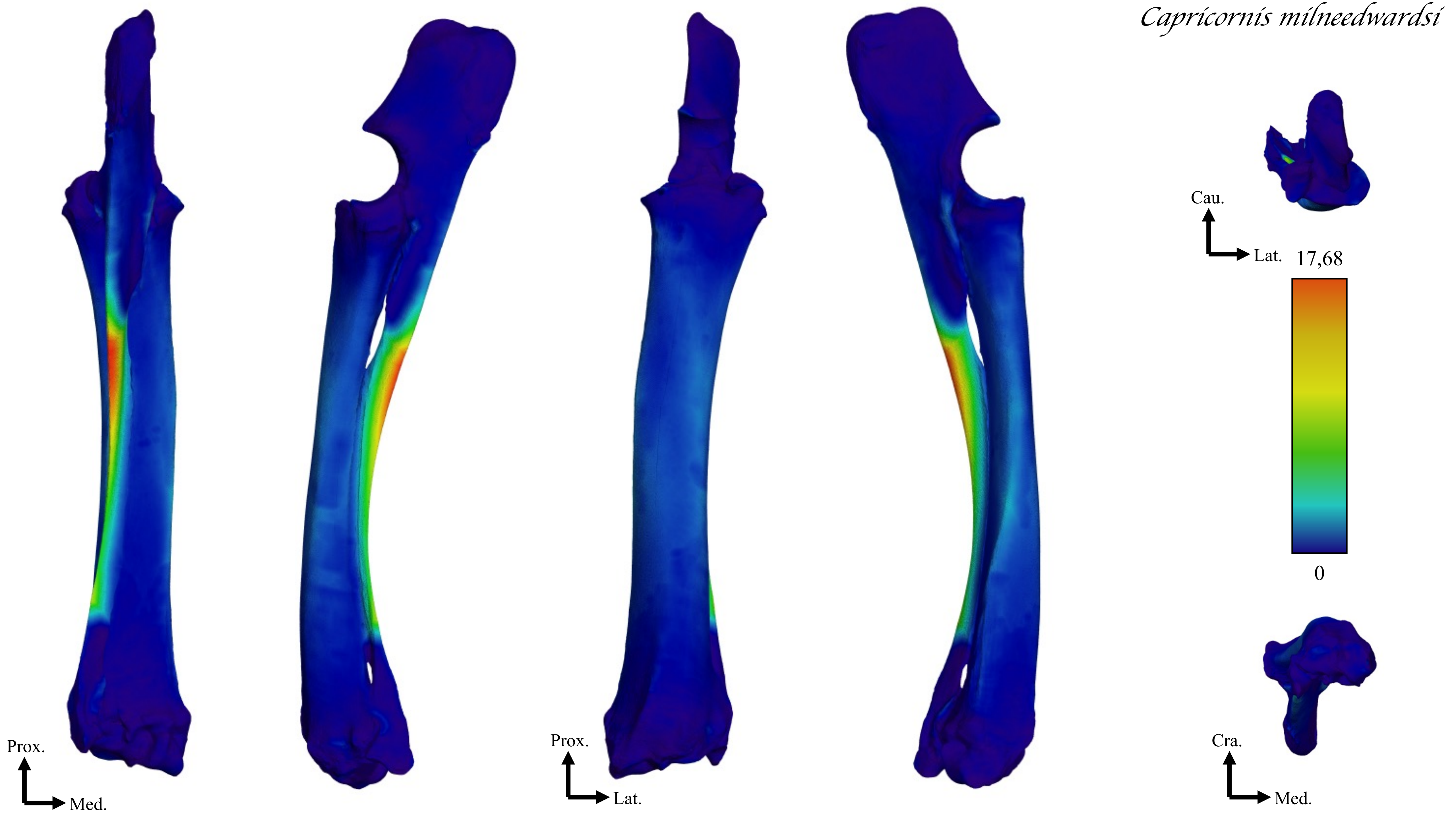

Capricornis milneedwardsi
Cau.
Lat.
17,68
0
Prox.
Med.
Prox.
Lat.
Cra.
Med.

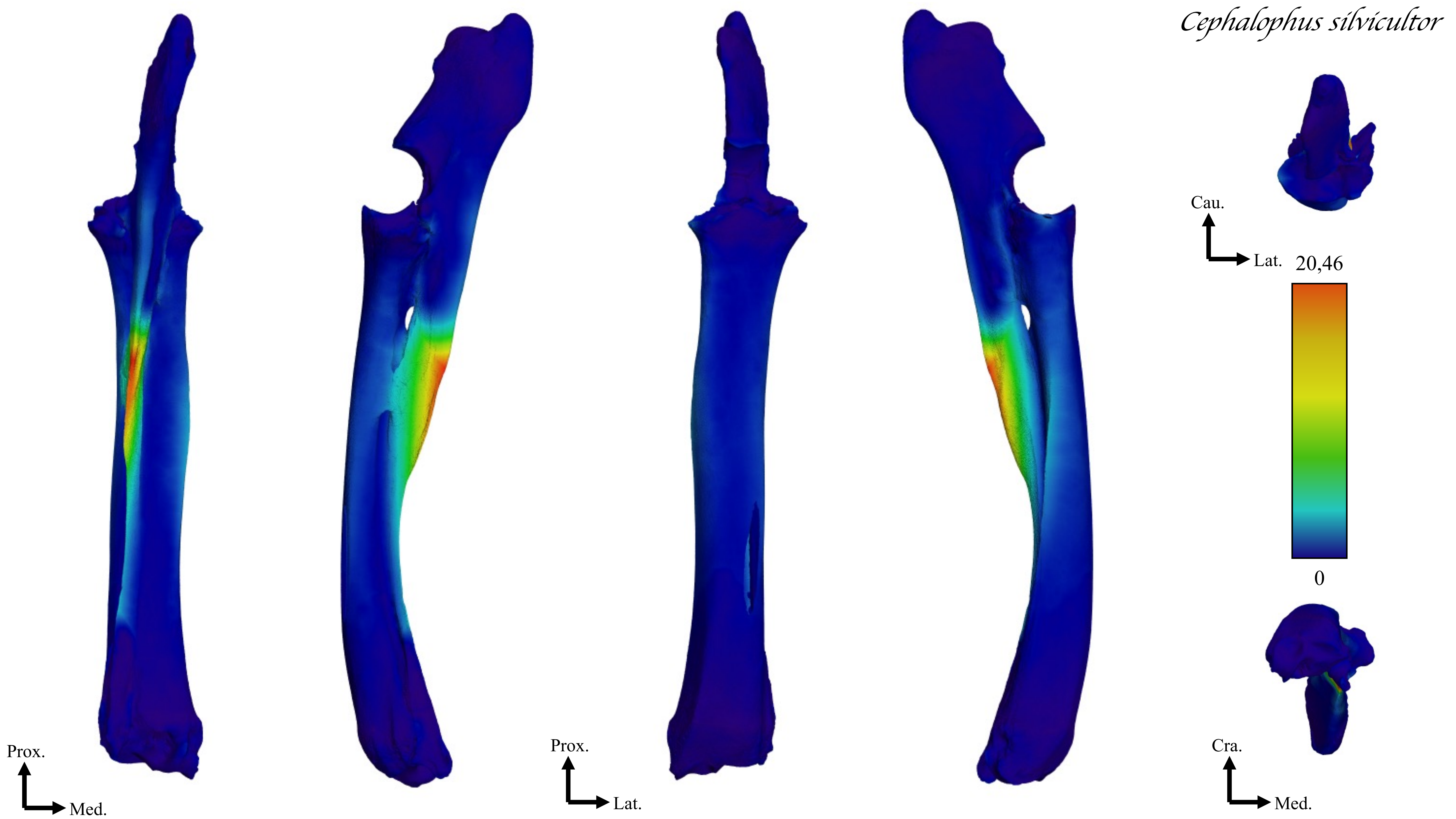

Cephalophus silvicultor
Cau.
Lat.
20,46
0
Prox.
Med.
Prox.
Lat.
Cra.
Med.

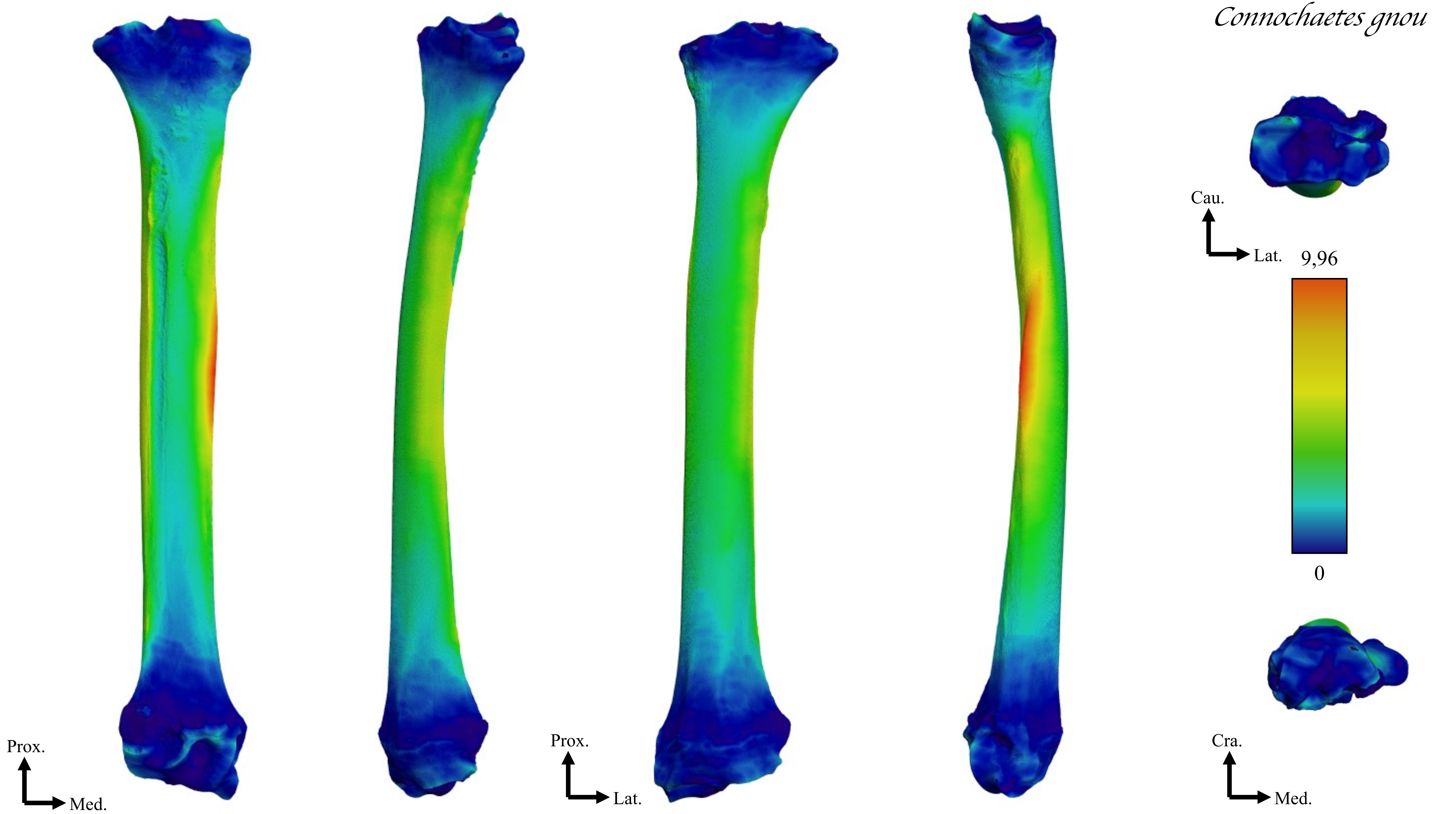
Connochaetes gnou
Cau.
Lat.
9,96
0
Prox.
Med.
Prox.
Lat.
Cra.
Med.

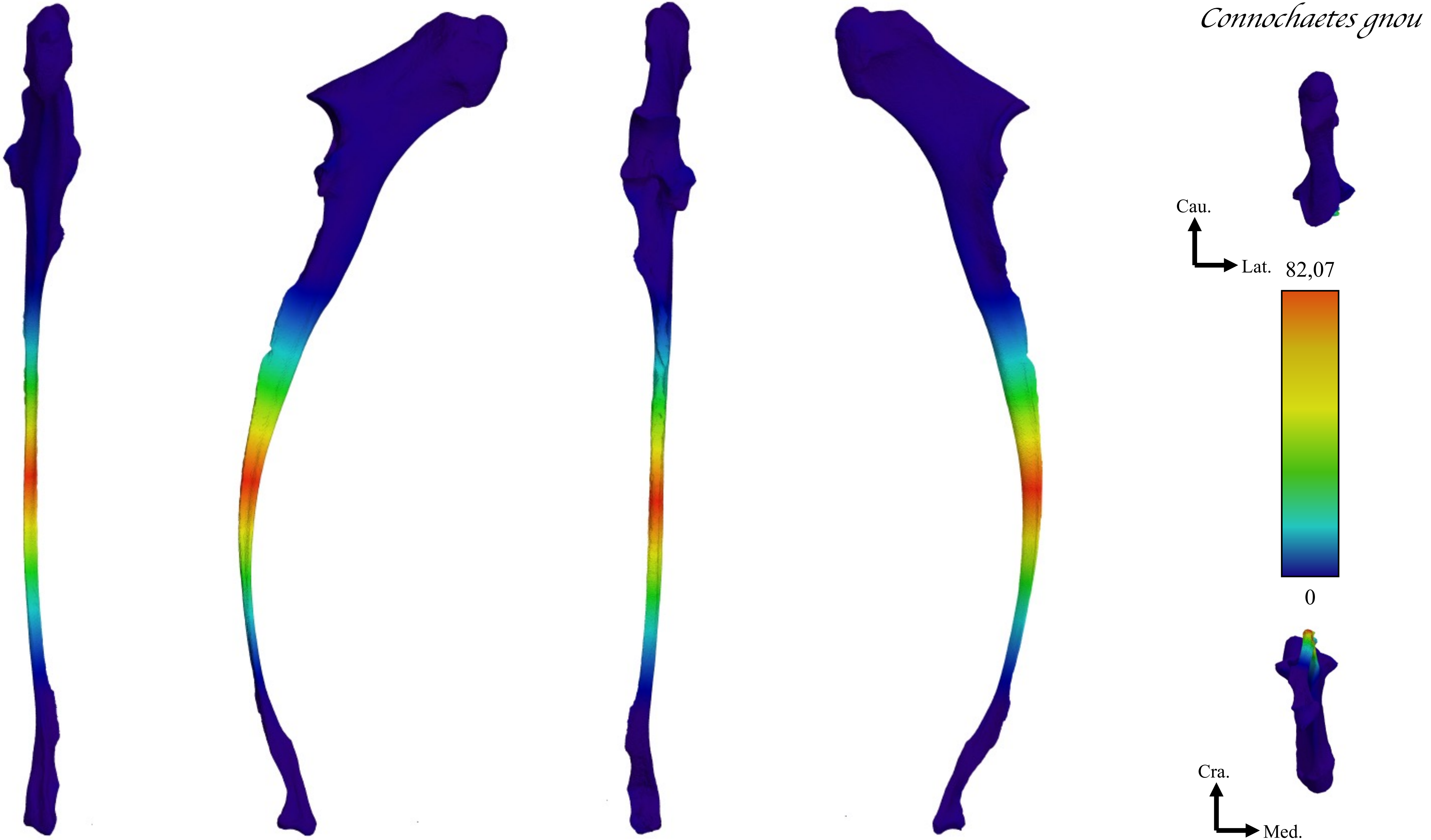

Connochaetes gnou
Cau.
Lat.
82,07
0
Cra.
Med.

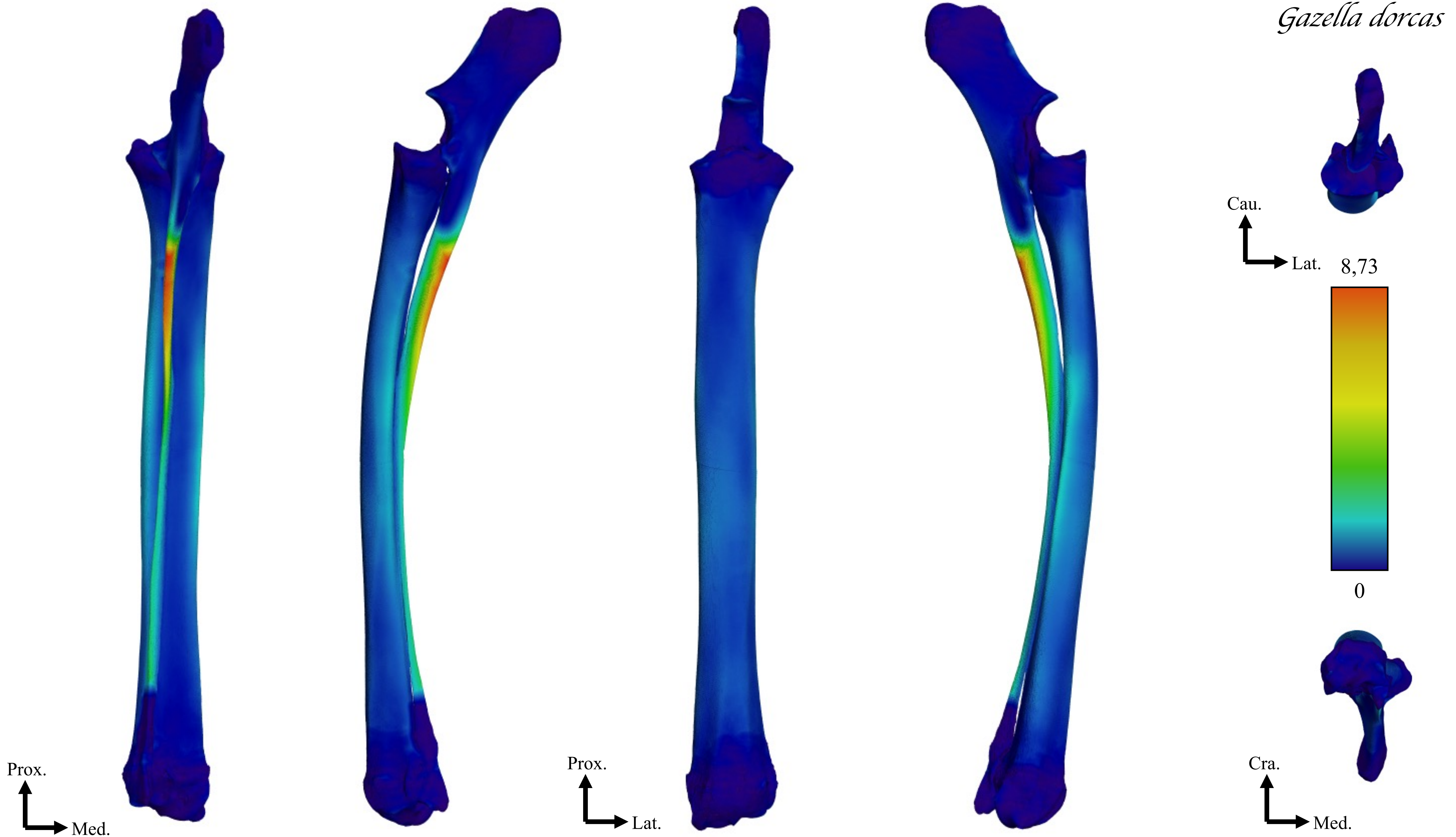

Gazella dorcas
Cau.
Lat.
8,73
0
Cra.
Med.
Prox.
Med.
Prox.
Lat.

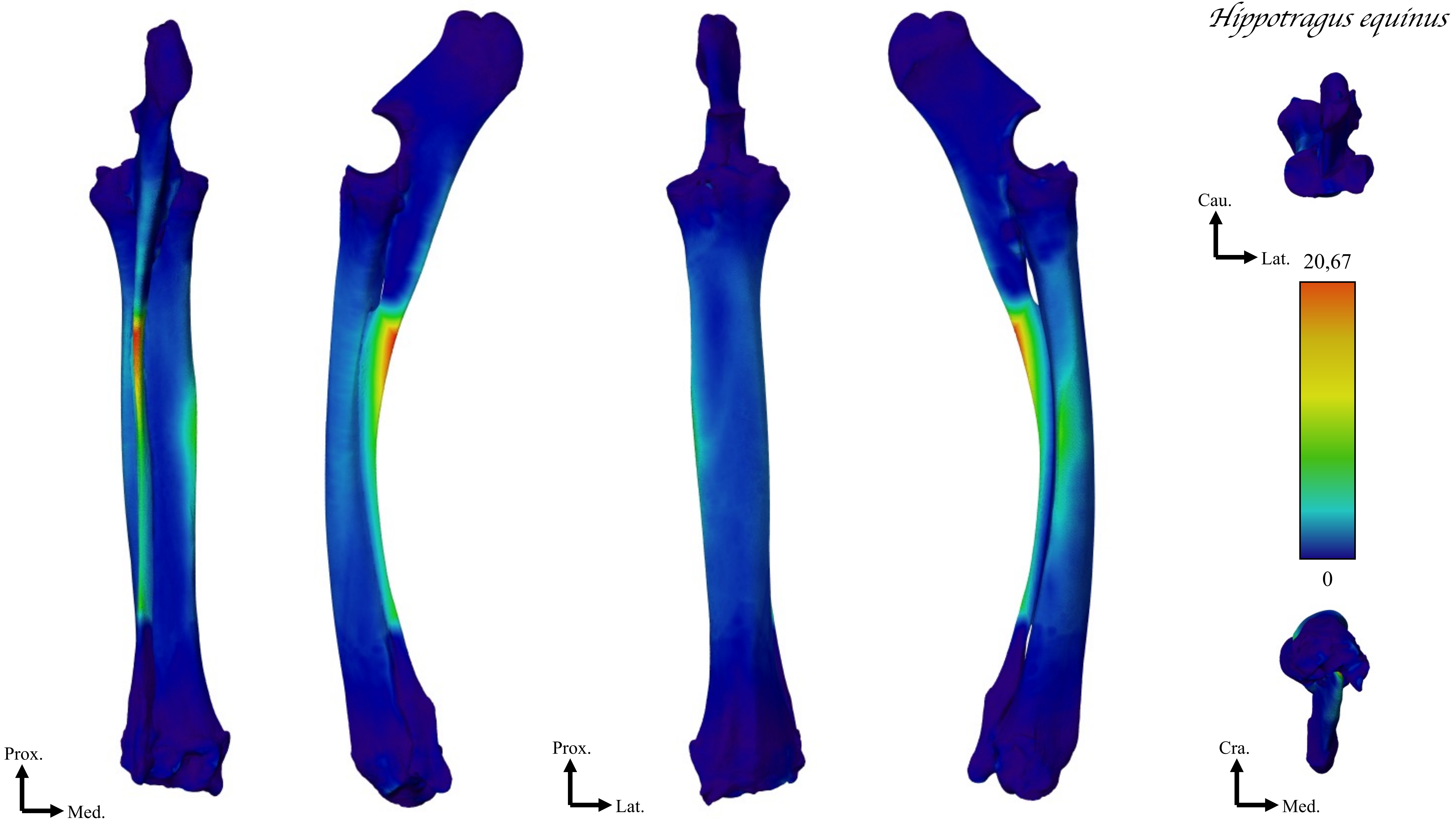

Hippotragus equinus
Cau.
Lat.
20,67
0
Cra.
Med.
Prox.
Med.
Prox.
Lat.

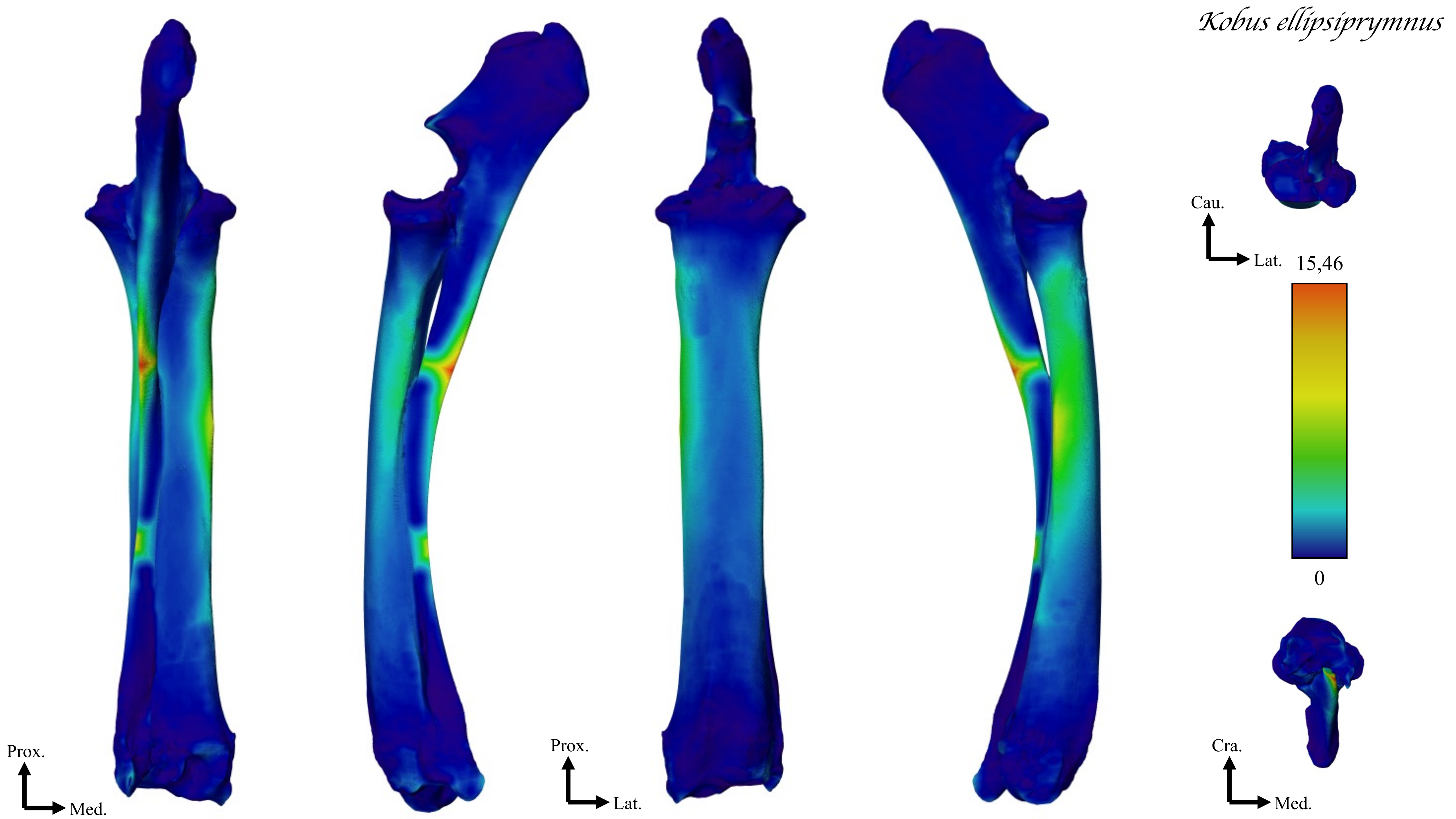
Kobus ellipsiprymnus
Cau.
Lat.
15,46
0
Cra.
Med.
Prox.
Med.
Prox.
Lat.

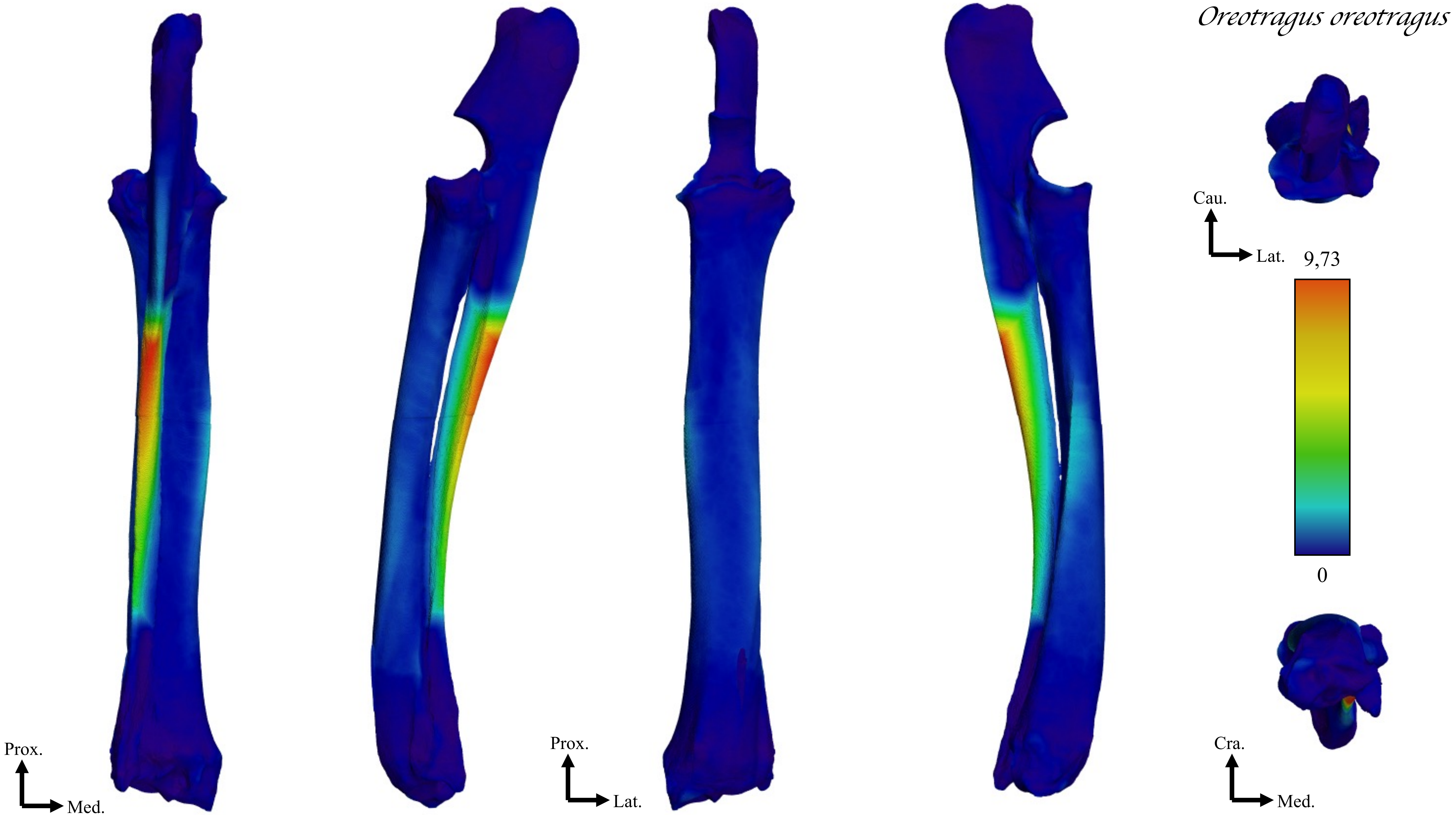

Oreotragus oreotragus
Cau.
Lat.
9,73
0
Cra.
Med.
Prox.
Med.
Prox.
Lat.

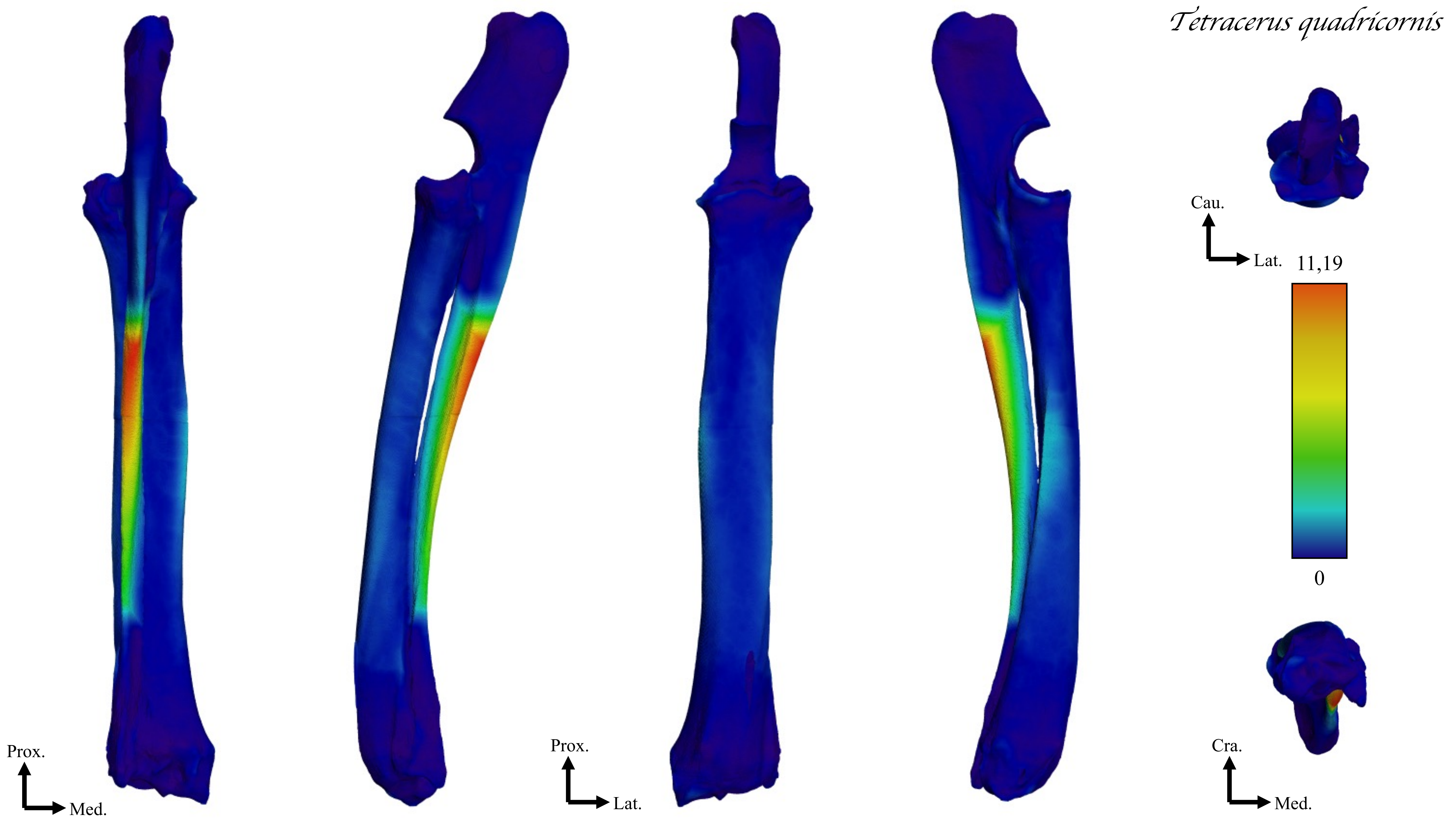

Tetracerus quadricornis
Cau.
Lat.
11,19
0
Cra.
Med.
Prox.
Med.
Prox.
Lat.
