# Supplementary material for: Microanatomical features of bovids long bones: What are the effects of mass and habitat?
Source: J Anat. 2026 Apr 23:10.1111/joa.70140. Online ahead of print. doi: 10.1111/joa.70140 (PMC13398651; doi:10.1111/joa.70140)
Supplement: Supplementary file 1 — Appendix S1. [file JOA-9999-0-s001.zip › S5_thickness_maps.zip!/Supdata5_thickness_maps/Supdata5_3_Femur.pdf]

Addax nasomaculatus

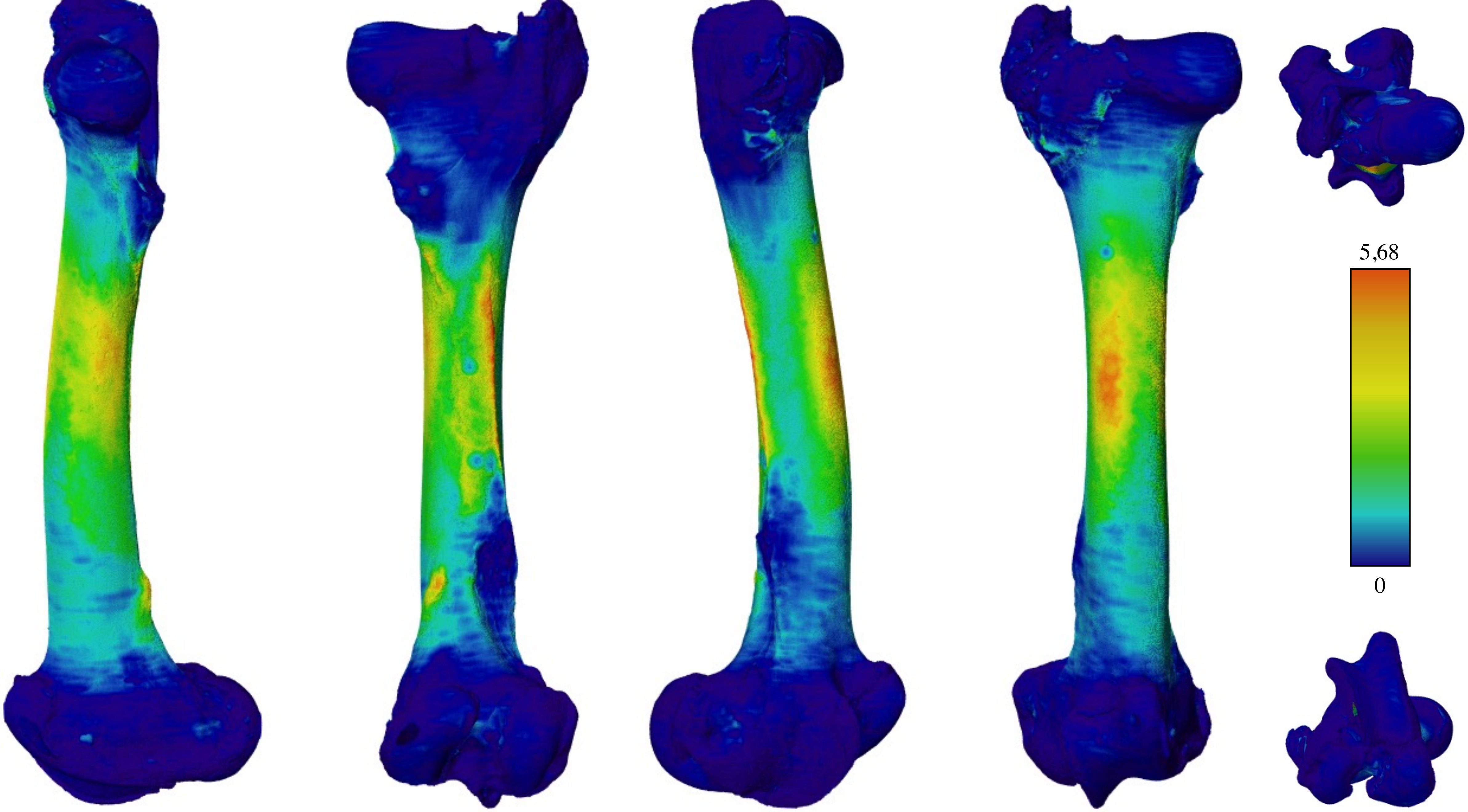

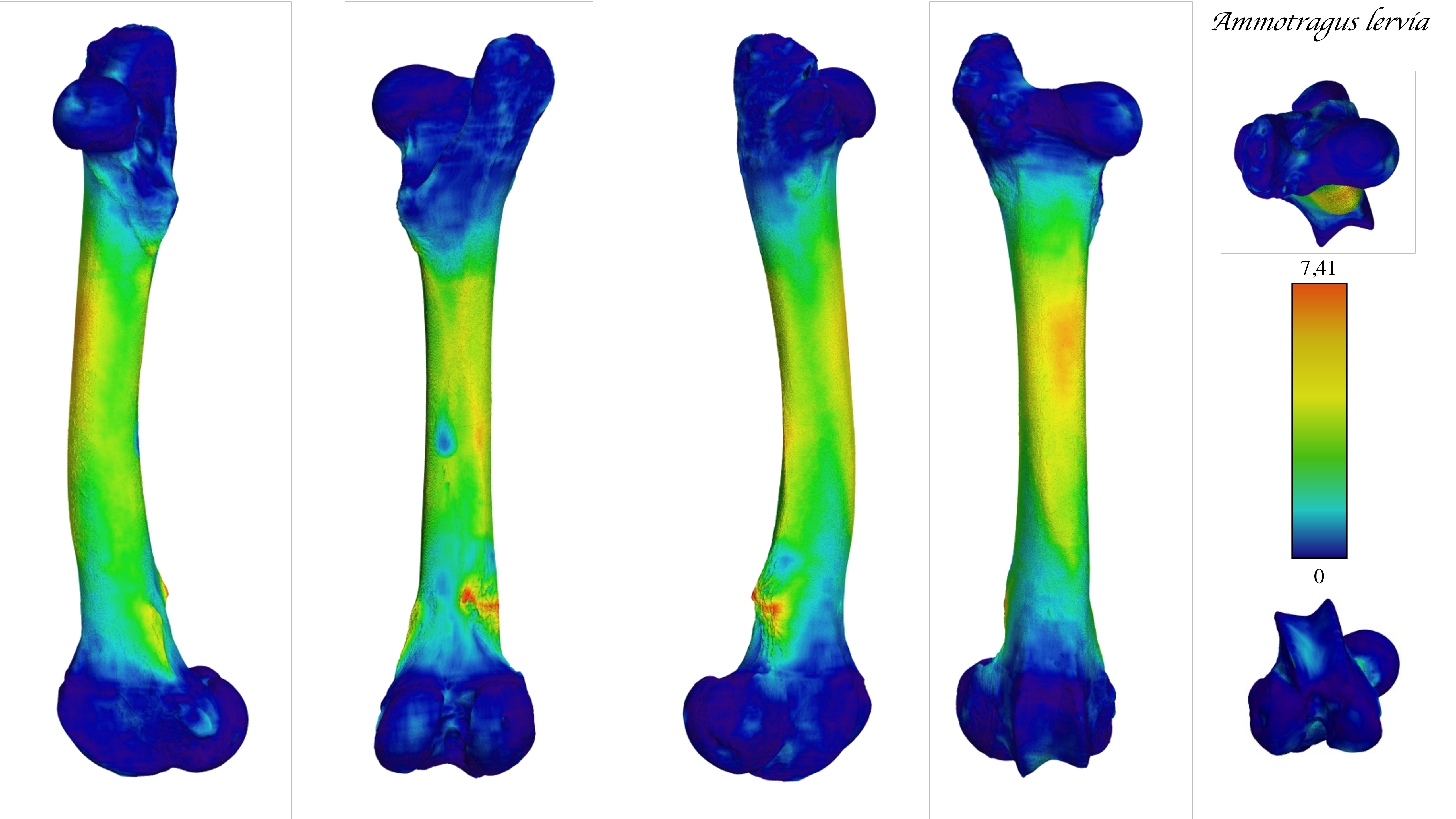

Ammotragus lervia
7,41
0

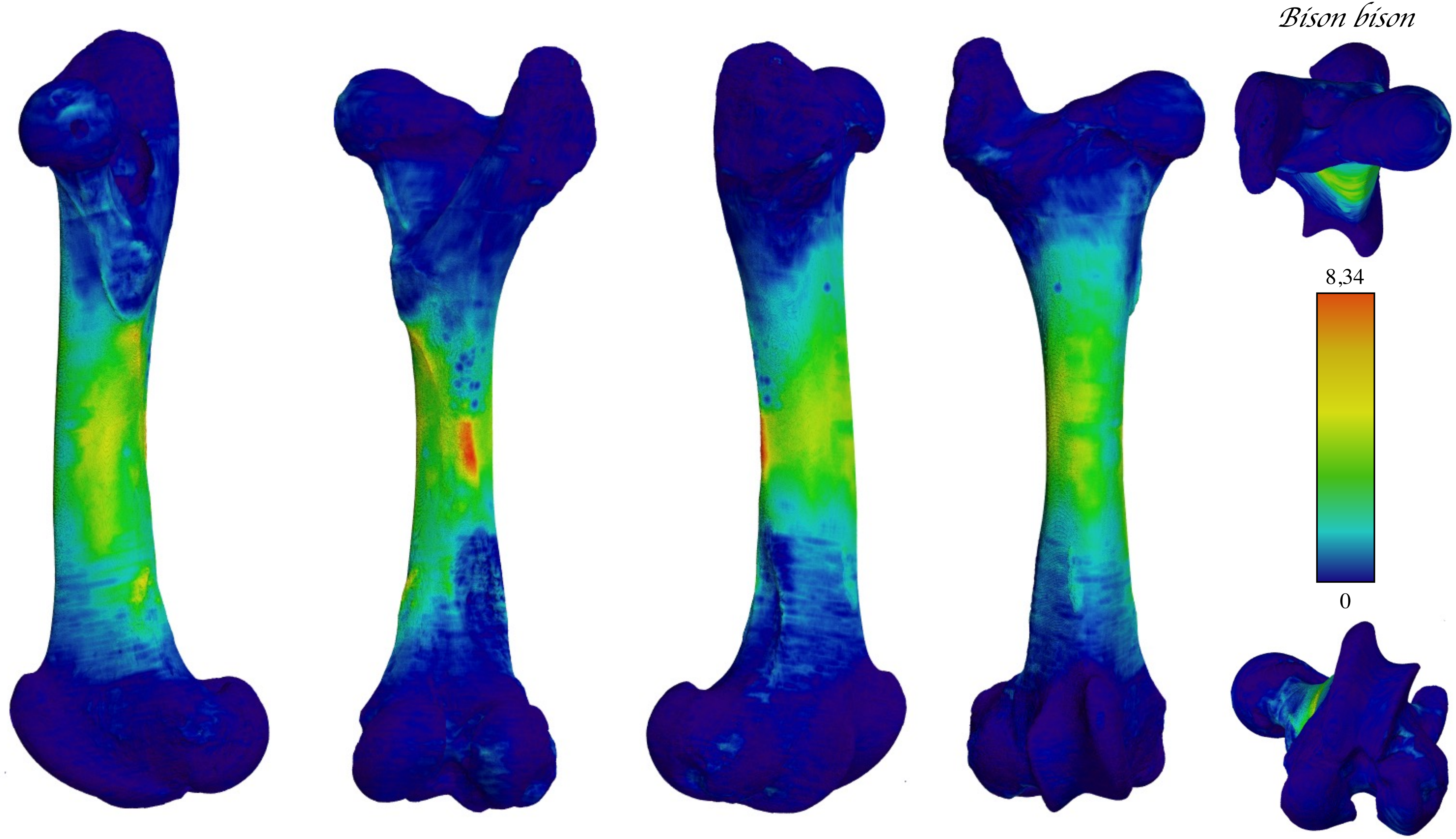
Bison bison
8,34
0

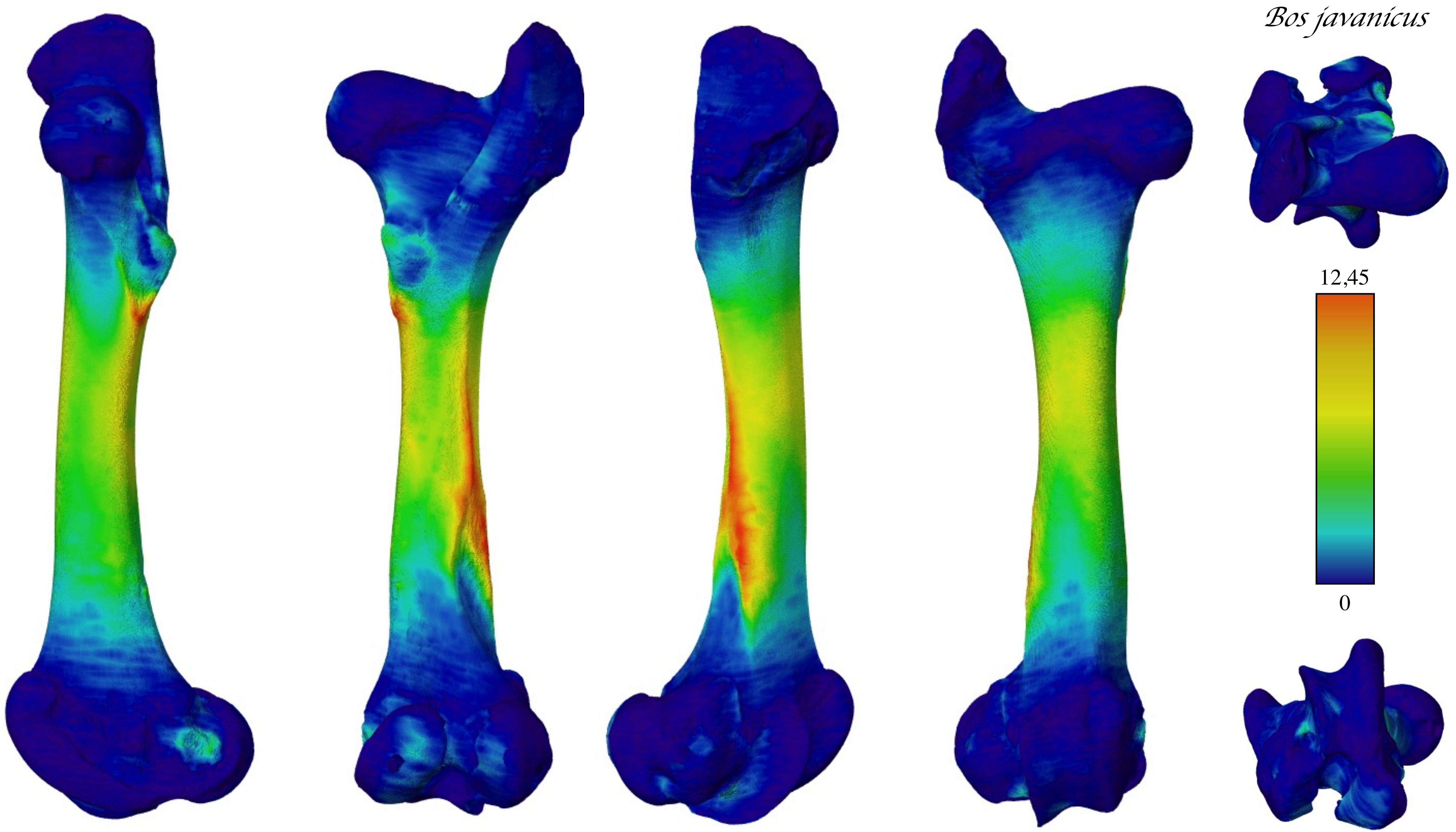
Bos javanicus
12,45
0

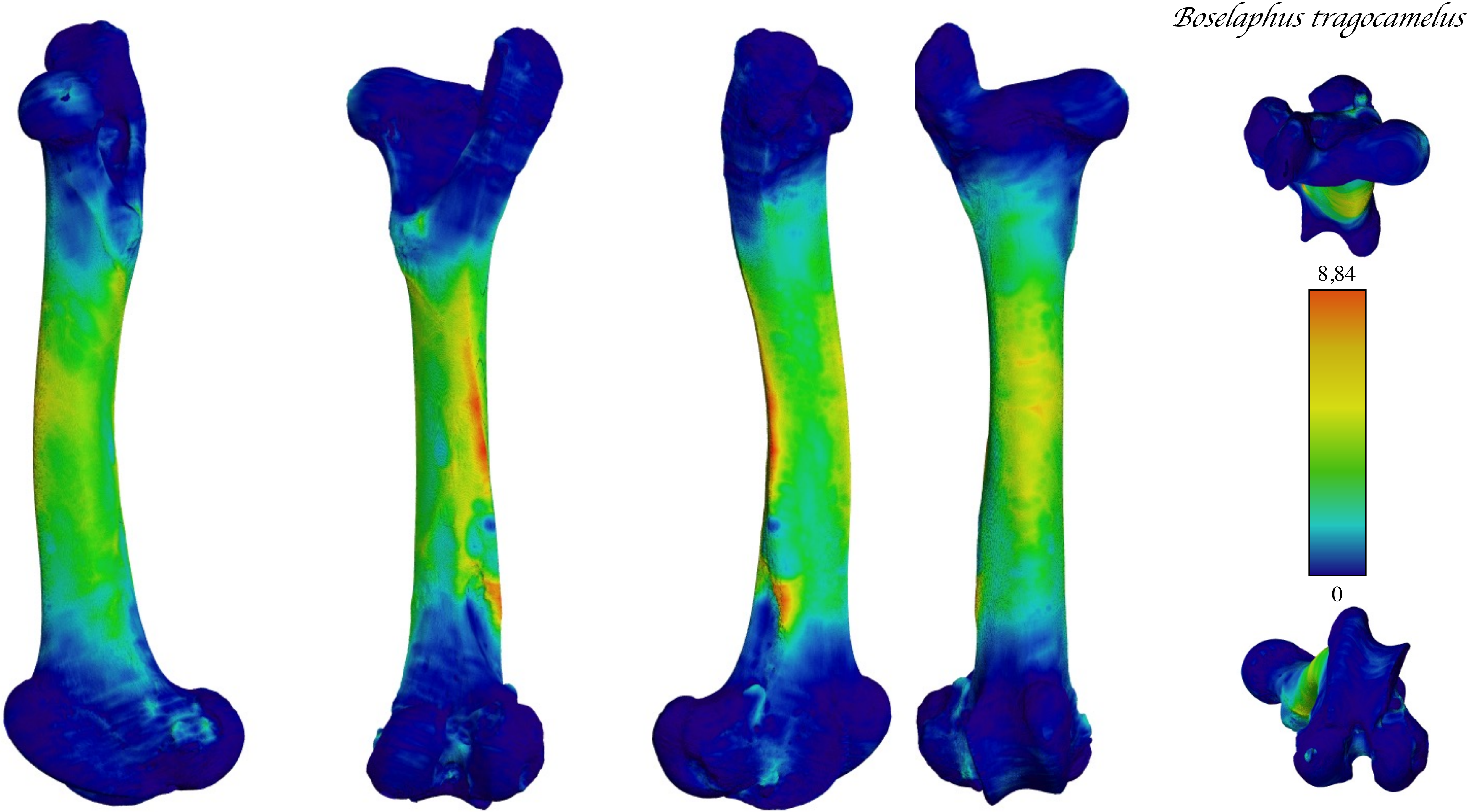
Boselaphus tragocamelus
8,84
0

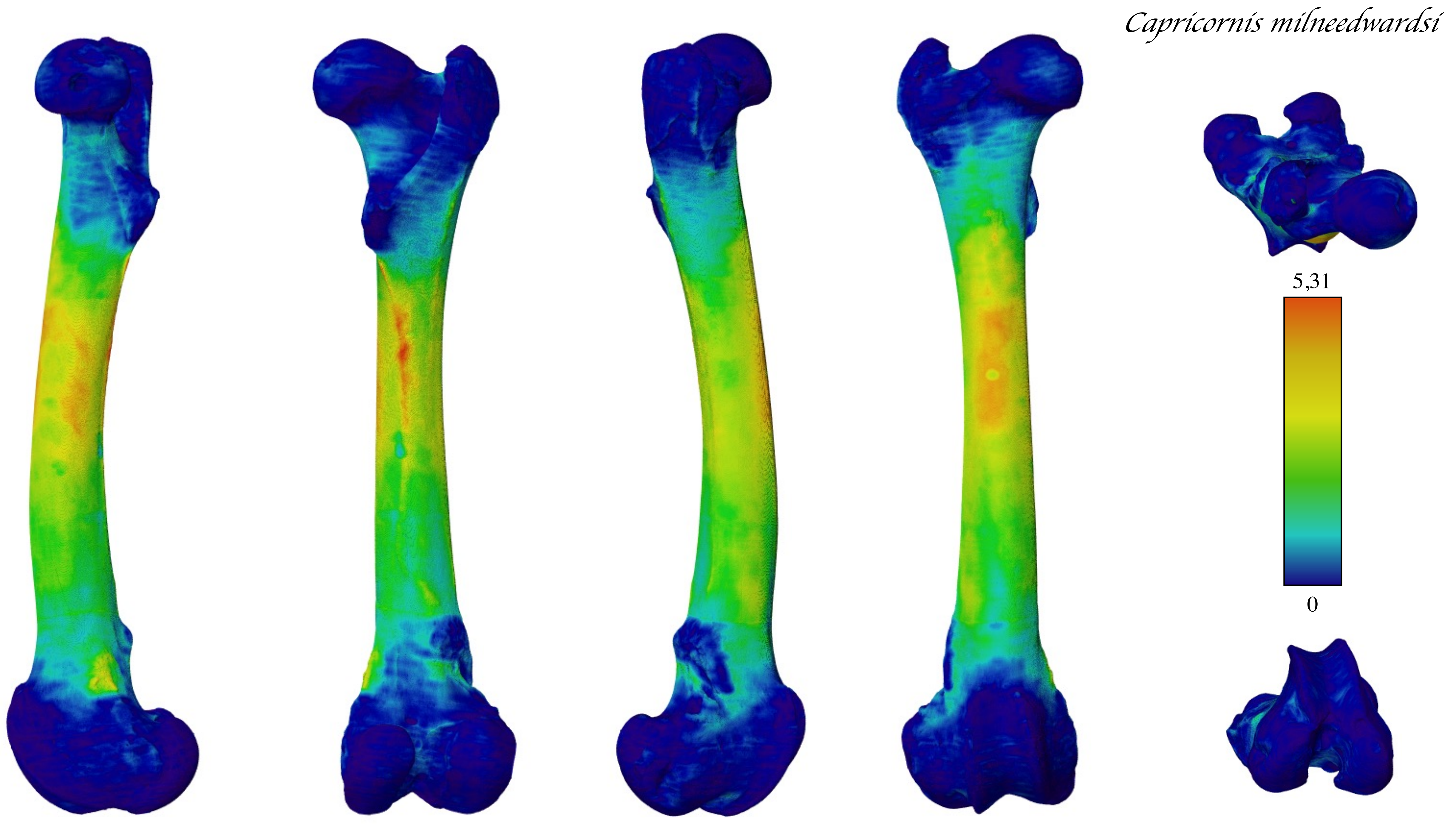
Capricornis milneedwardsi
5,31
0

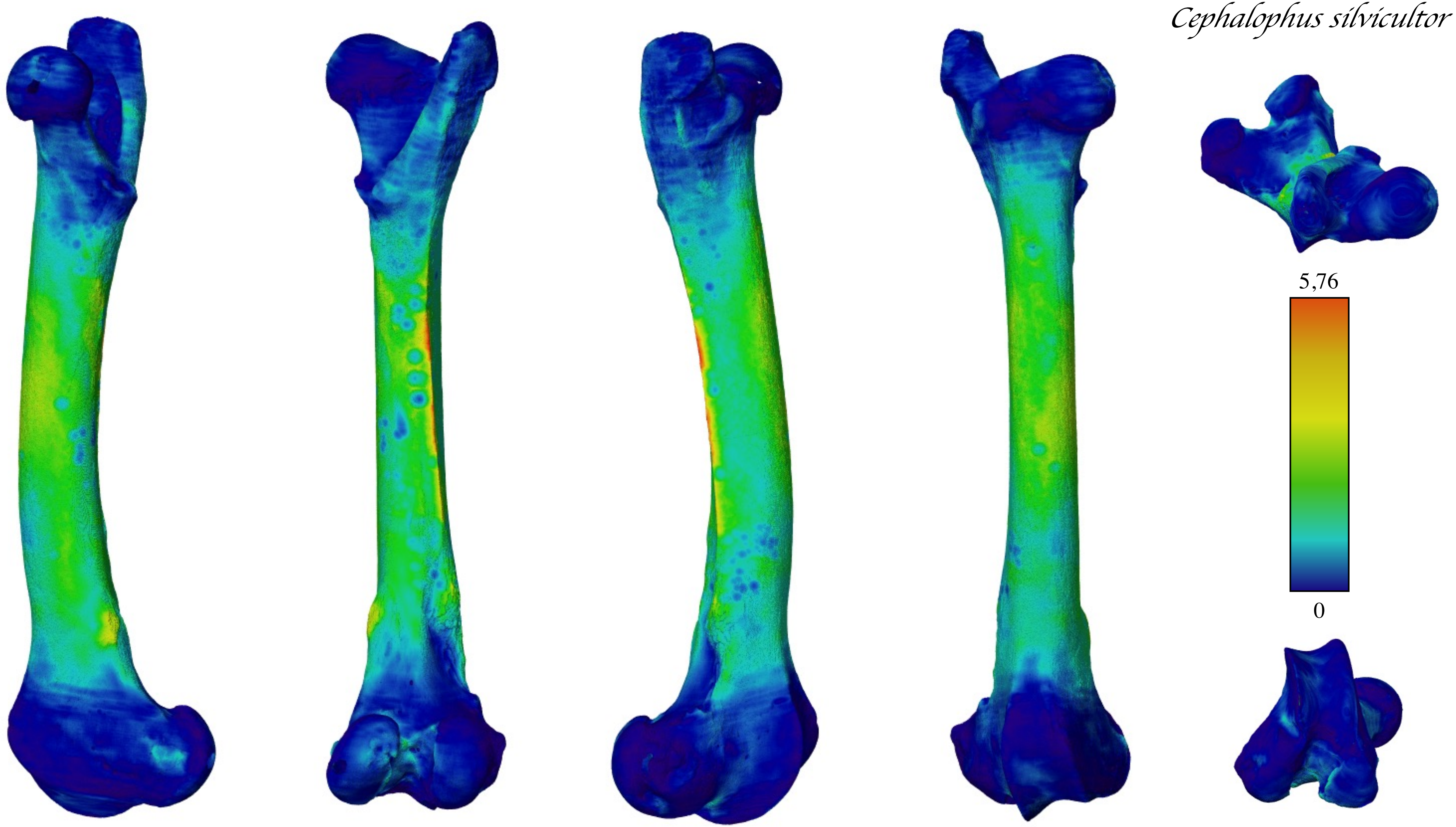
Cephalophus silvicultor
5,76
0

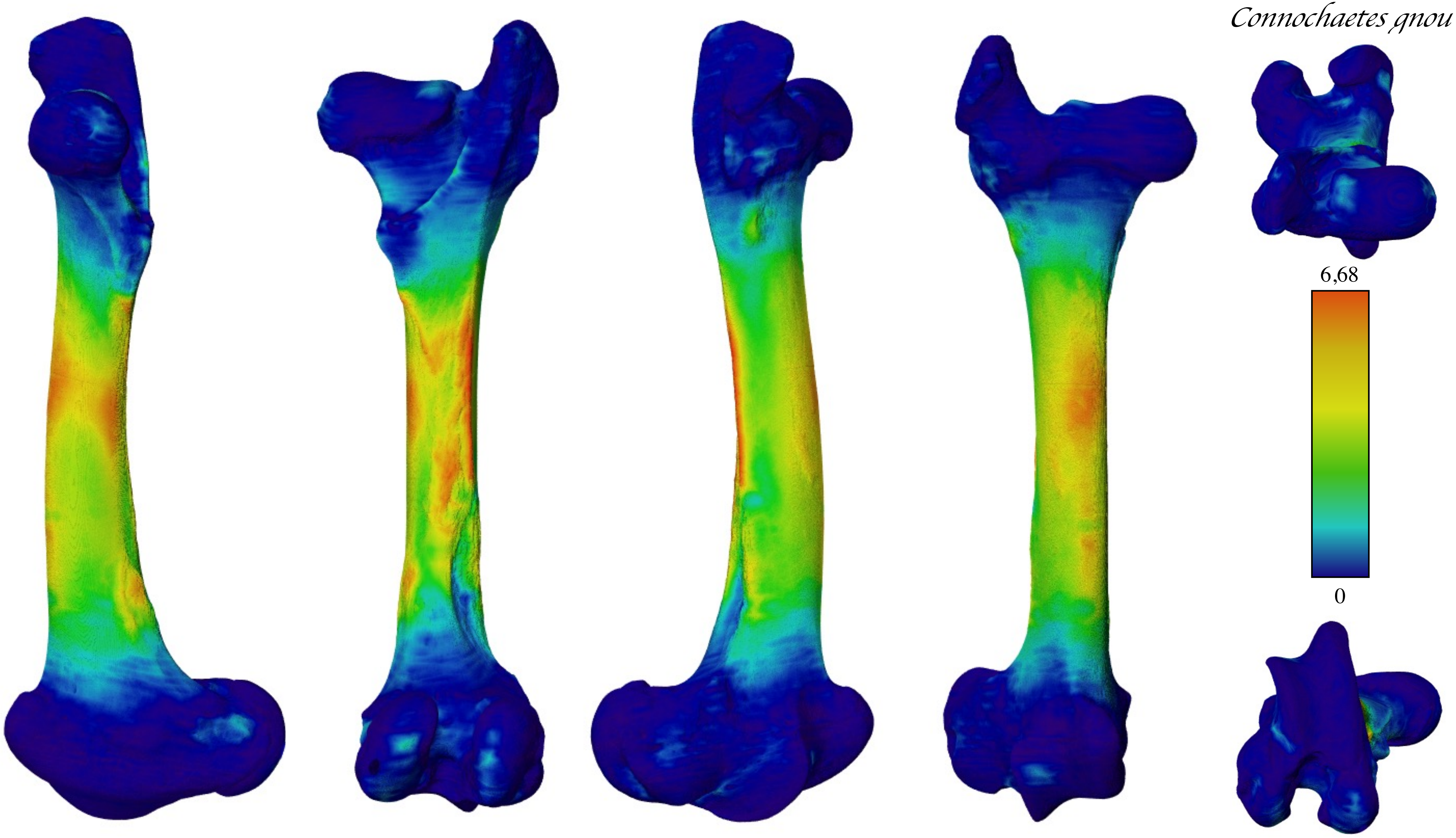

Connochaetes gnou
6,68
0

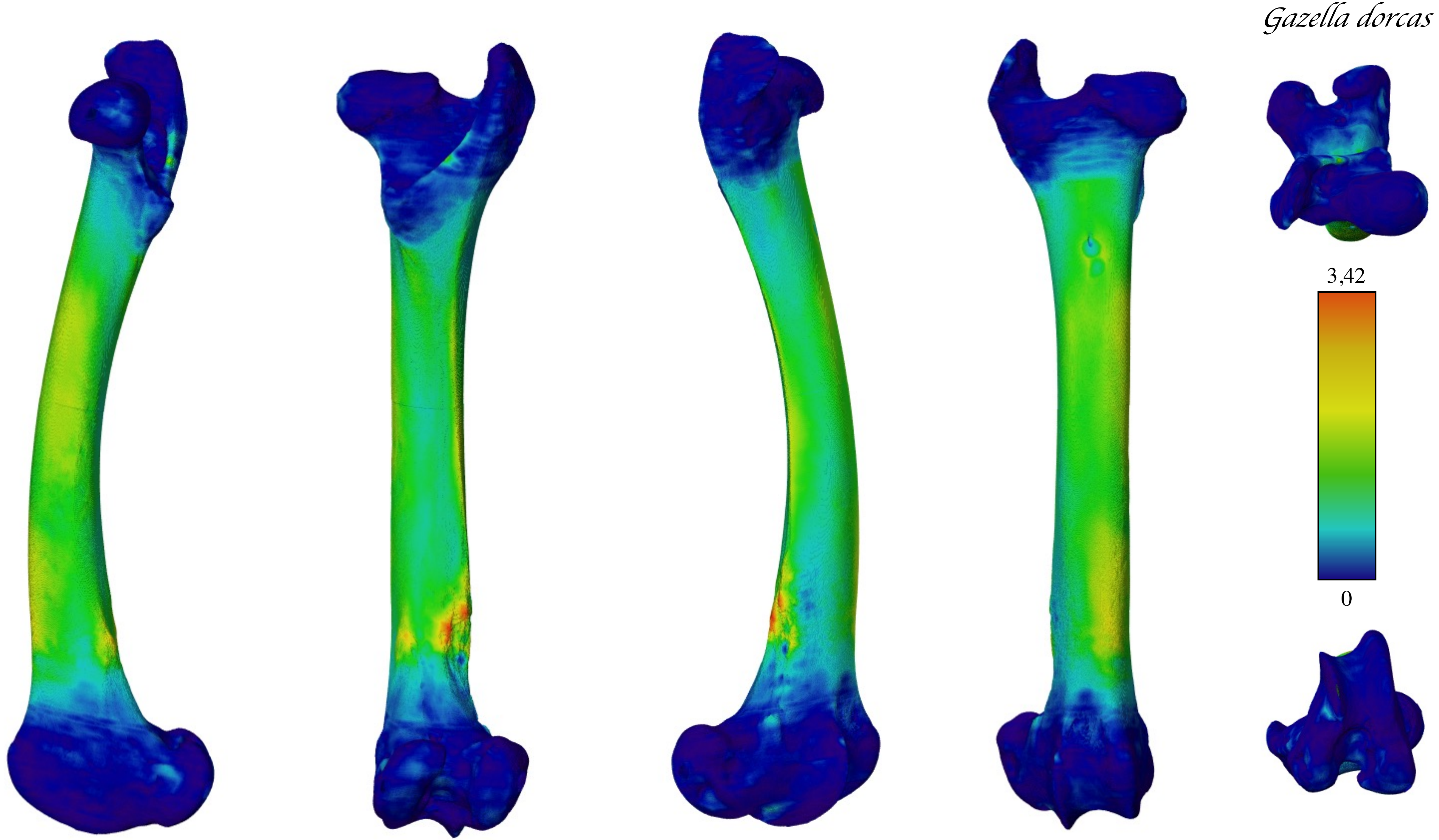
Gazella dorcas
3,42
0

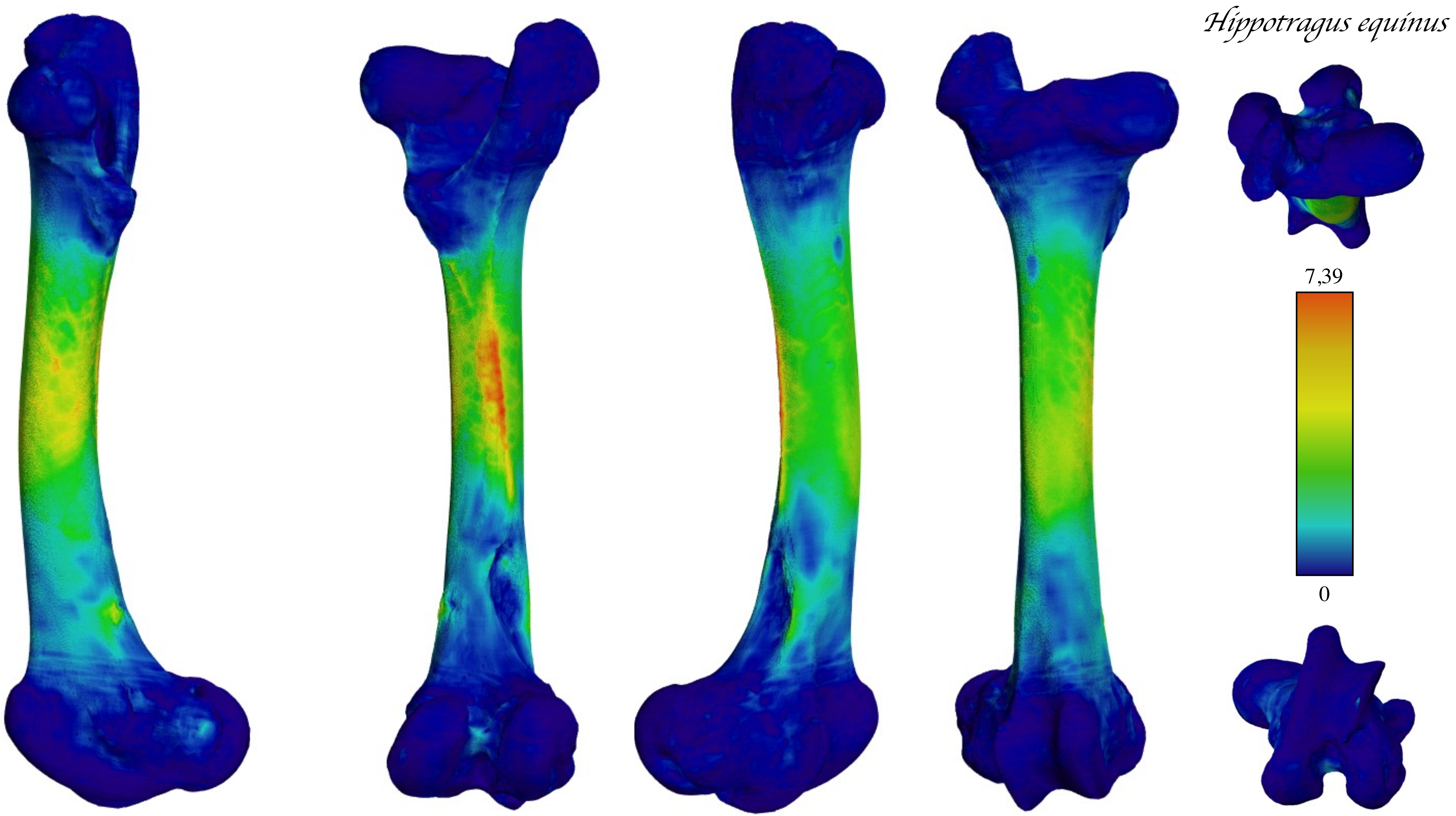

Hippotragus equinus
7,39
0

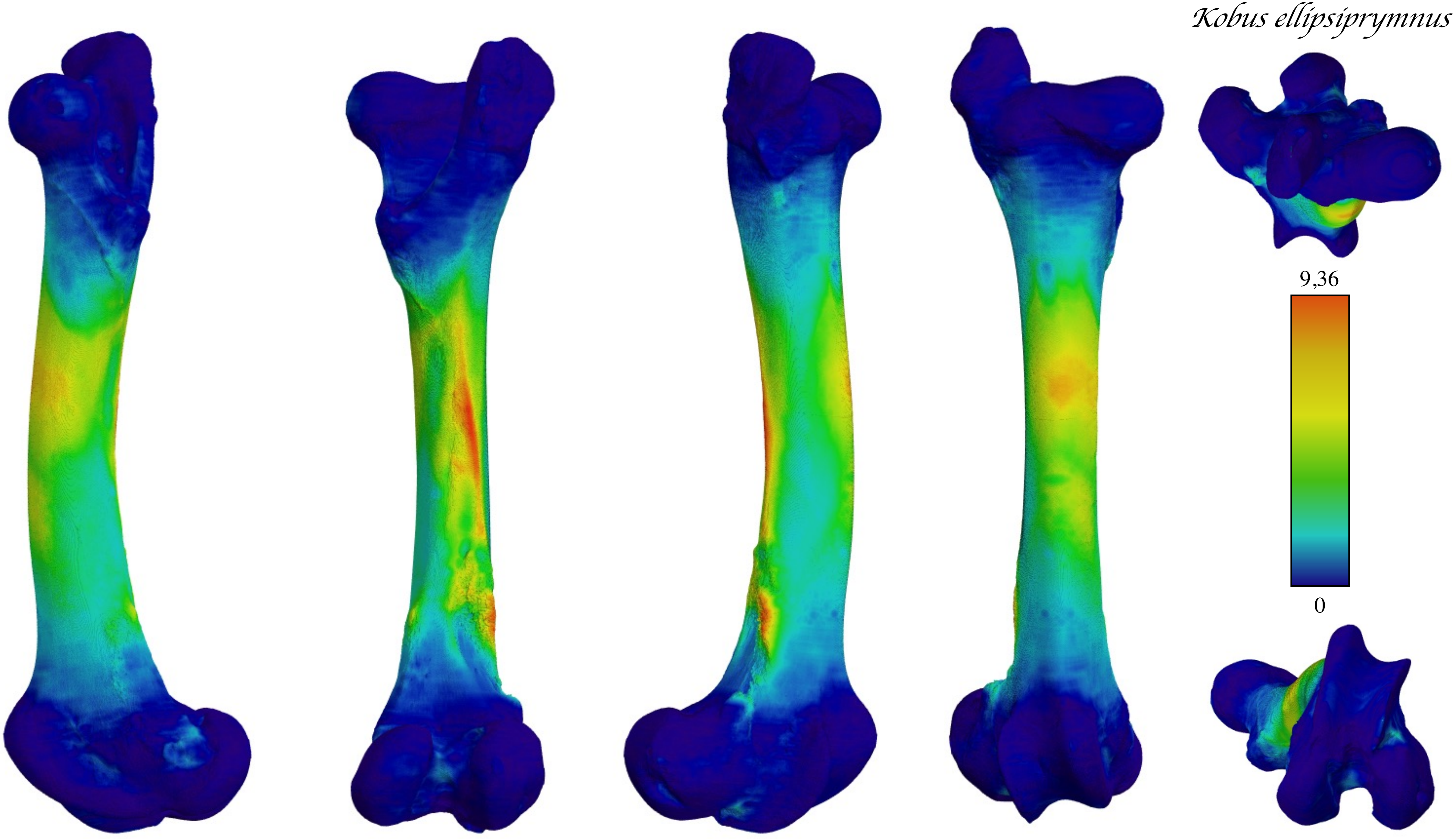
Kobus ellipsiprymnus
9,36
0

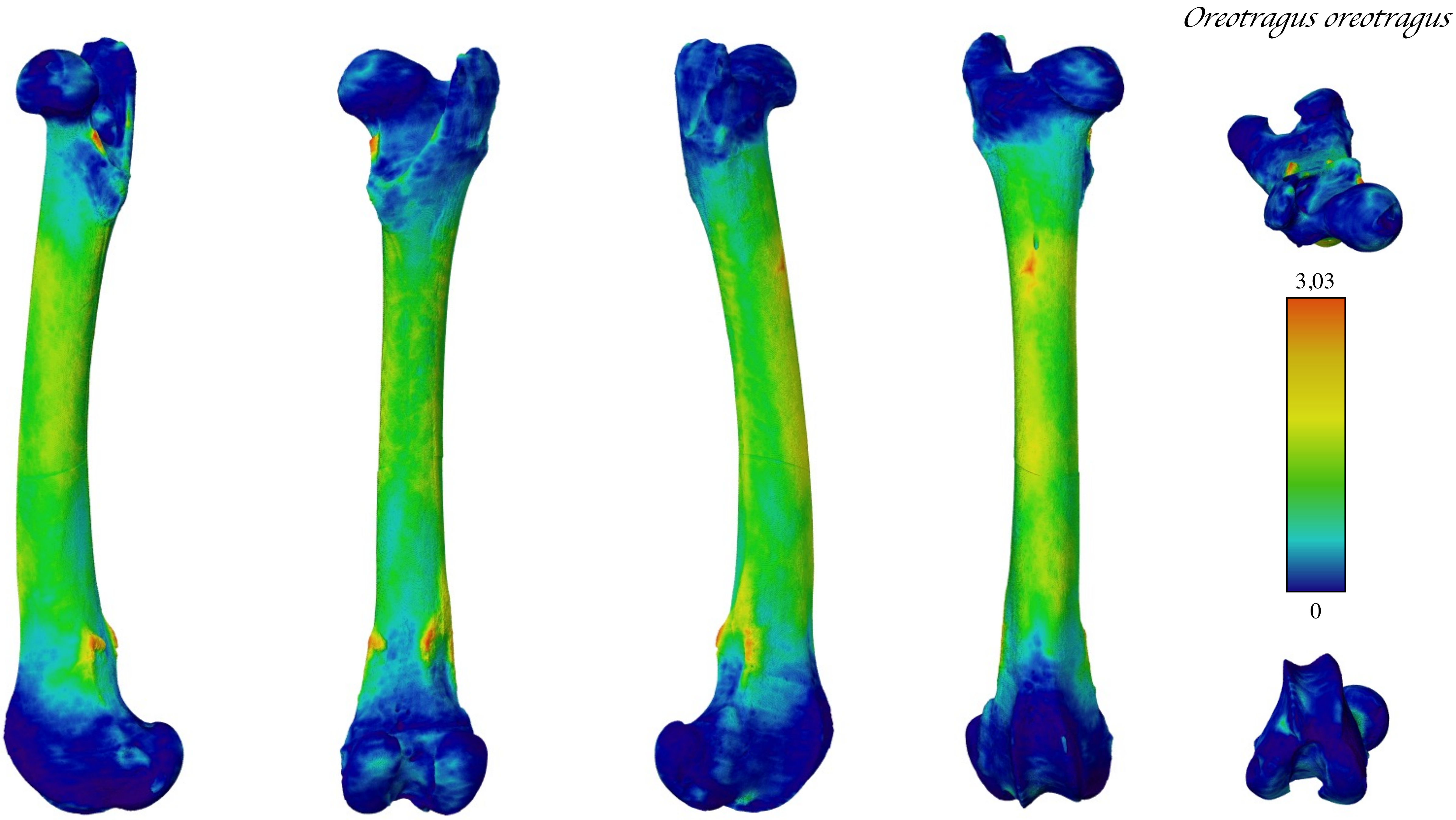
Oreotragus oreotragus
3,03
0

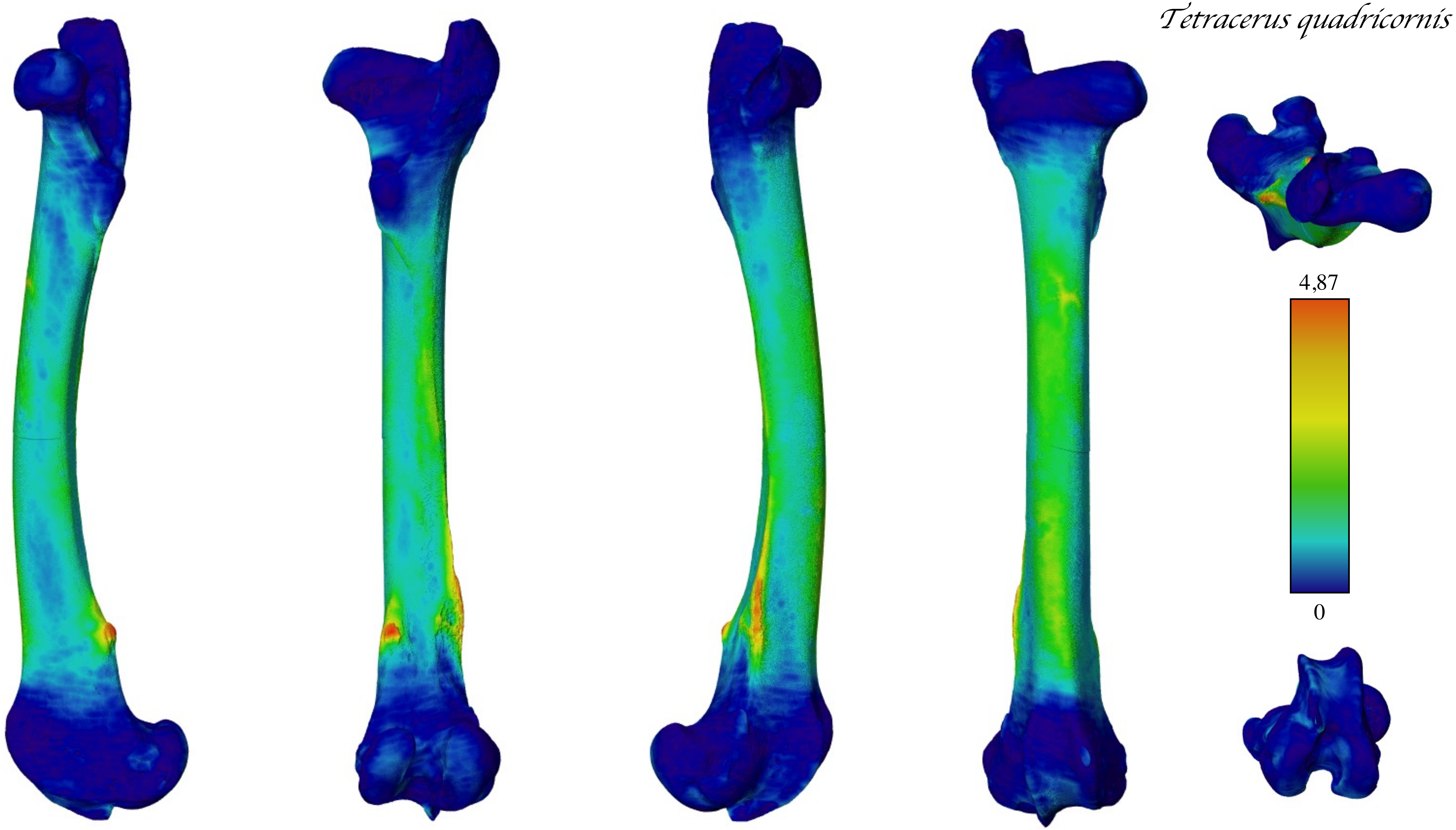

Tetracerus quadricornis
4,87
0
