# Supplementary material for: Microanatomical features of bovids long bones: What are the effects of mass and habitat?
Source: J Anat. 2026 Apr 23:10.1111/joa.70140. Online ahead of print. doi: 10.1111/joa.70140 (PMC13398651; doi:10.1111/joa.70140)
Supplement: Supplementary file 1 — Appendix S1. [file JOA-9999-0-s001.zip › S5_thickness_maps.zip!/Supdata5_thickness_maps/Supddata5_4_tibia.pdf]

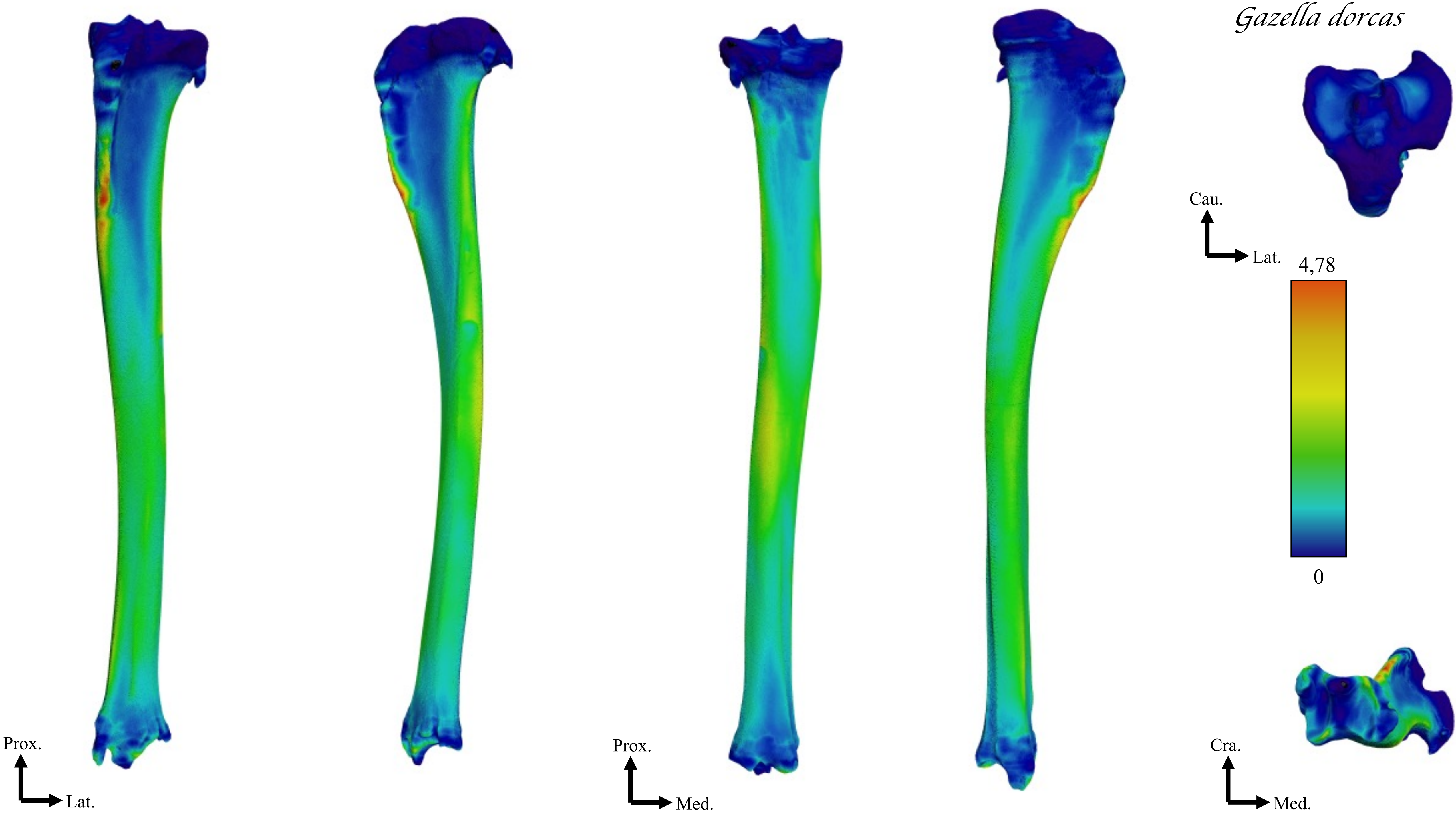

Gazella dorcas
Cau.
Lat.
4,78
0
Prox.
Lat.
Prox.
Med.
Cra.
Med.

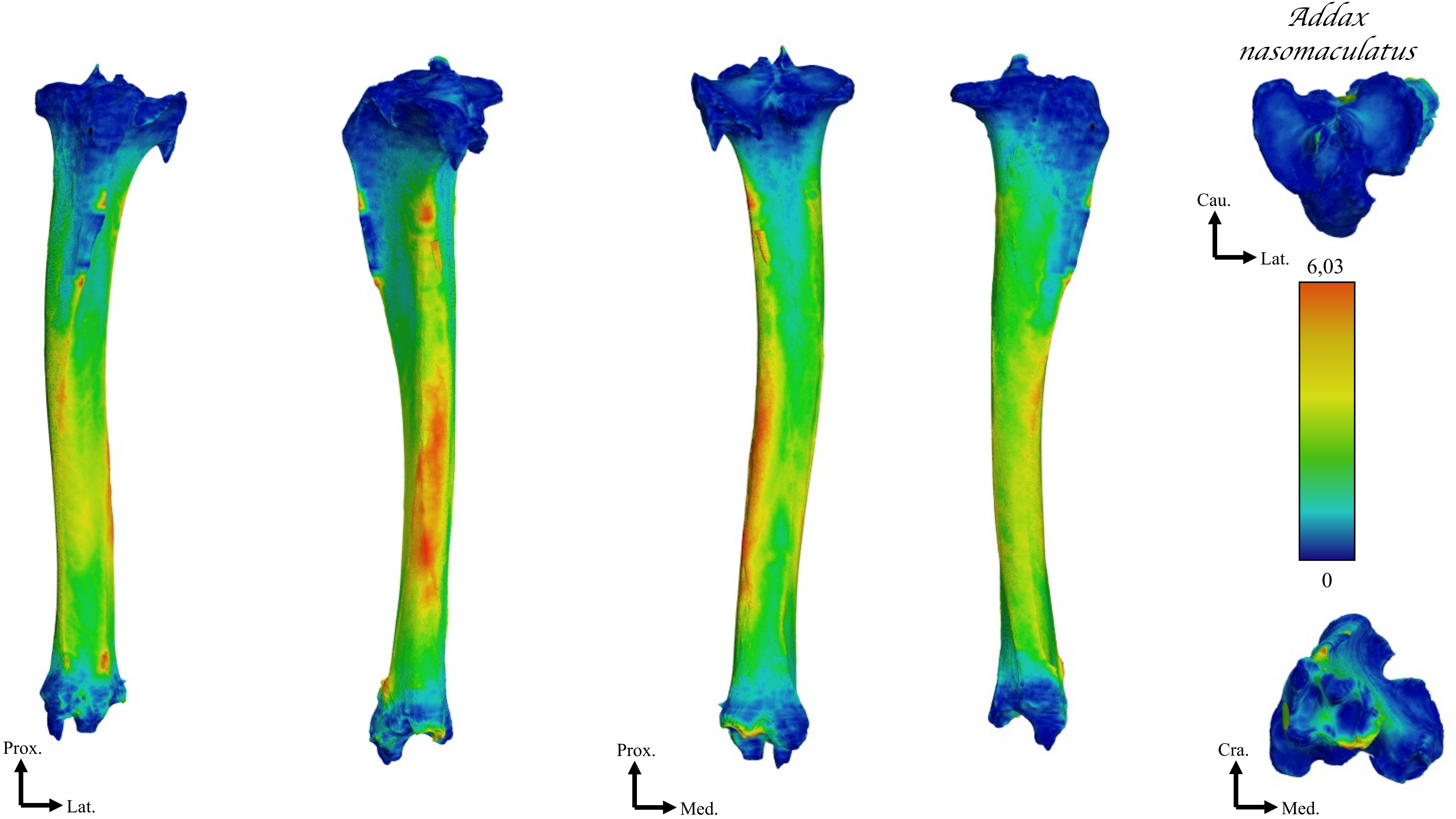
Addax nasomaculatus
Cau.
Lat.
6,03
0
Prox.
Lat.
Prox.
Med.
Cra.
Med.

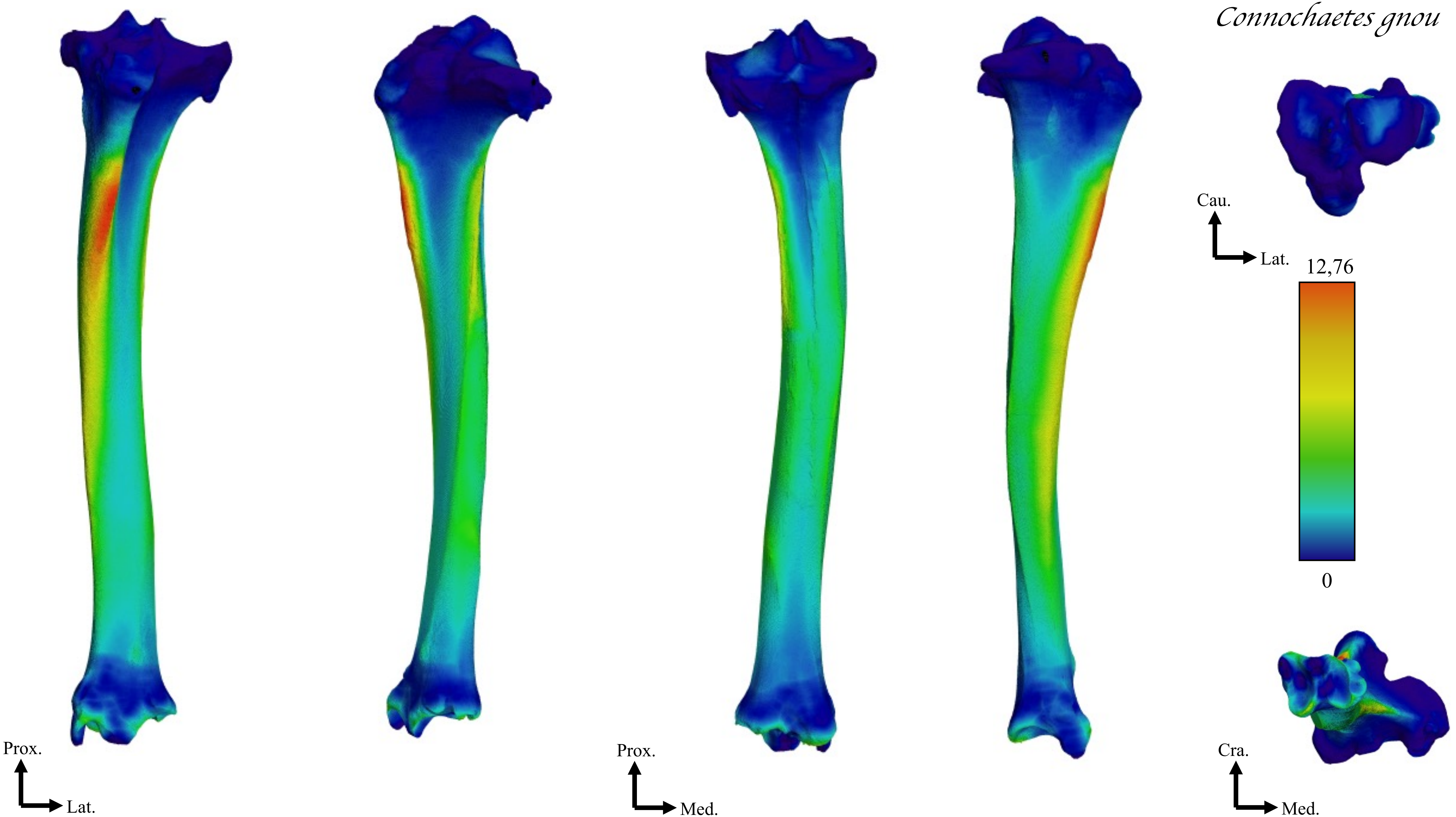

Connochaetes gnou
Cau.
Lat.
12,76
0
Prox.
Lat.
Prox.
Med.
Cra.
Med.

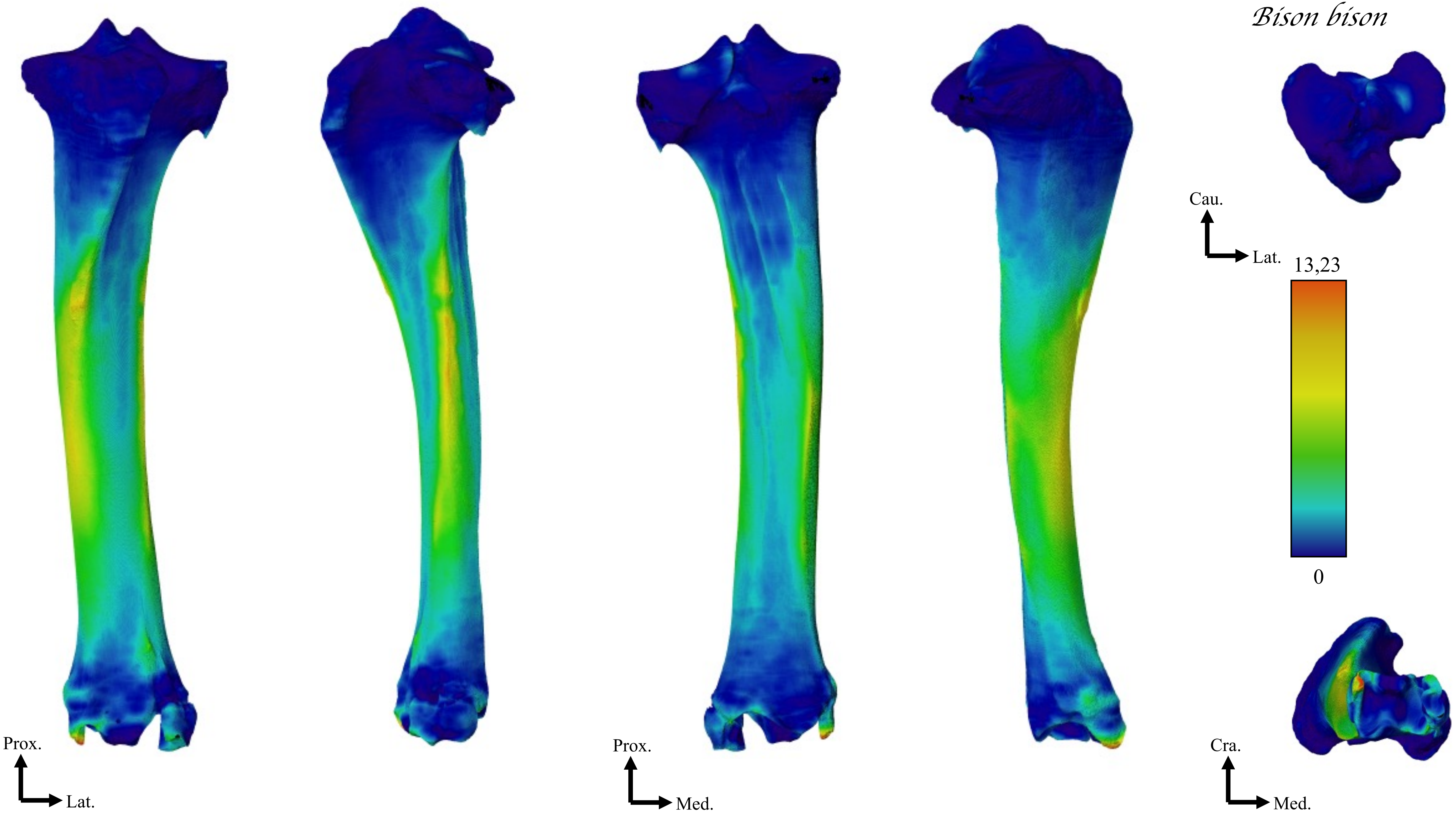
Bison bison
Cau.
Lat.
13,23
0
Prox.
Lat.
Prox.
Med.
Cra.
Med.

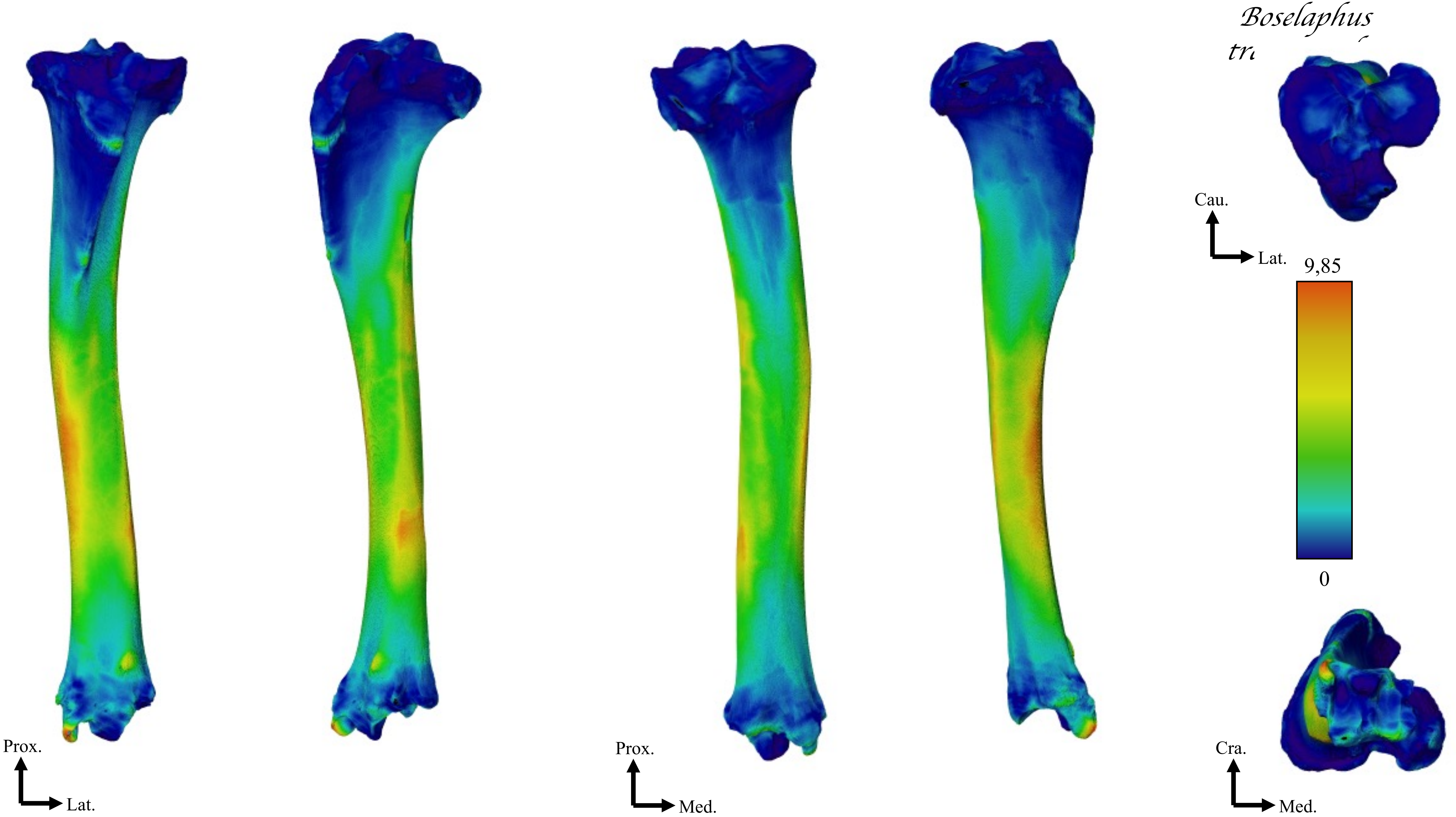
Boselaphus
Cau.
Lat.
9,85
0
Prox.
Lat.
Prox.
Med.
Cra.
Med.

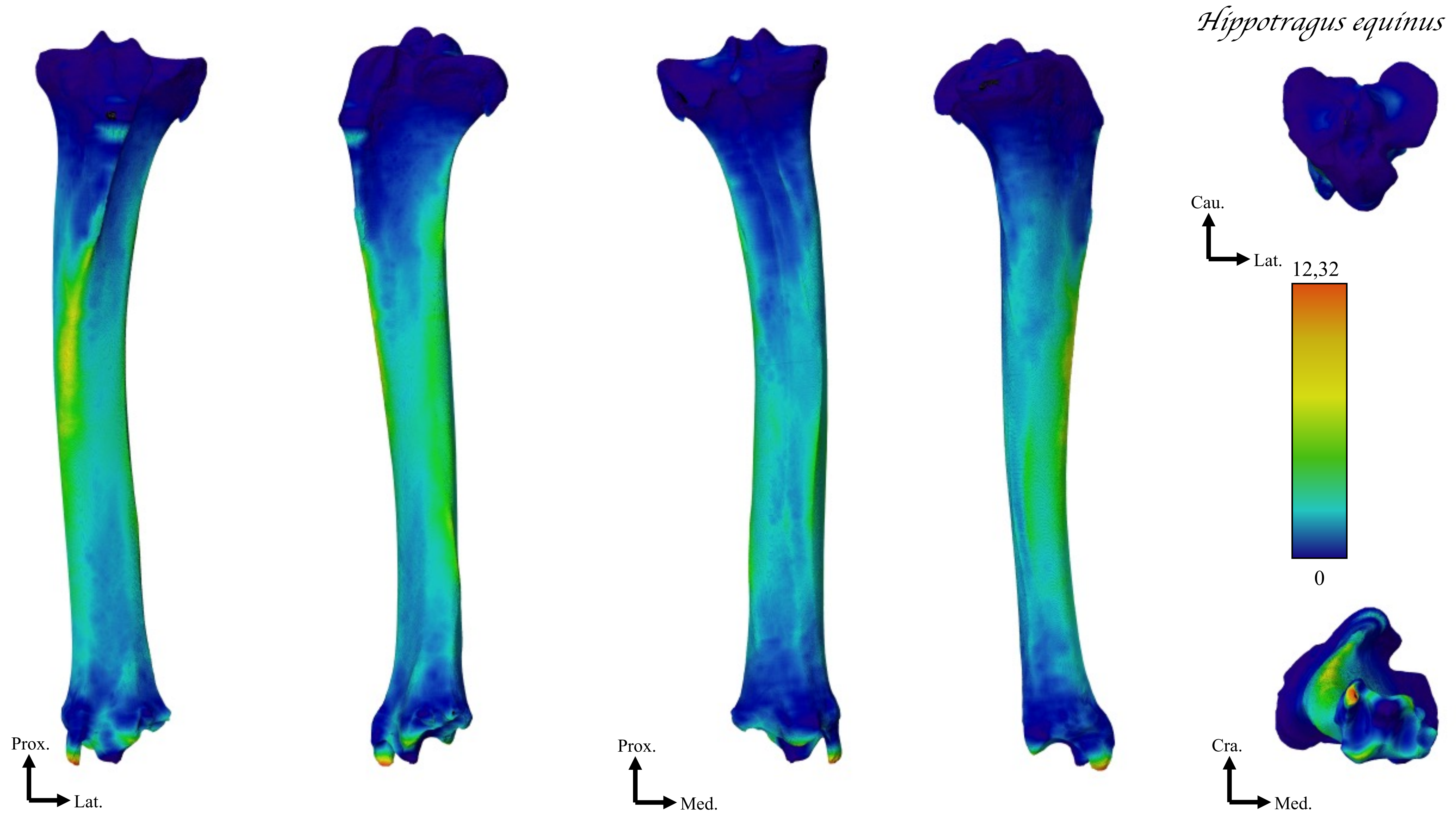
Hippotragus equinus
Cau.
Lat.
12,32
0
Prox.
Lat.
Prox.
Med.
Cra.
Med.

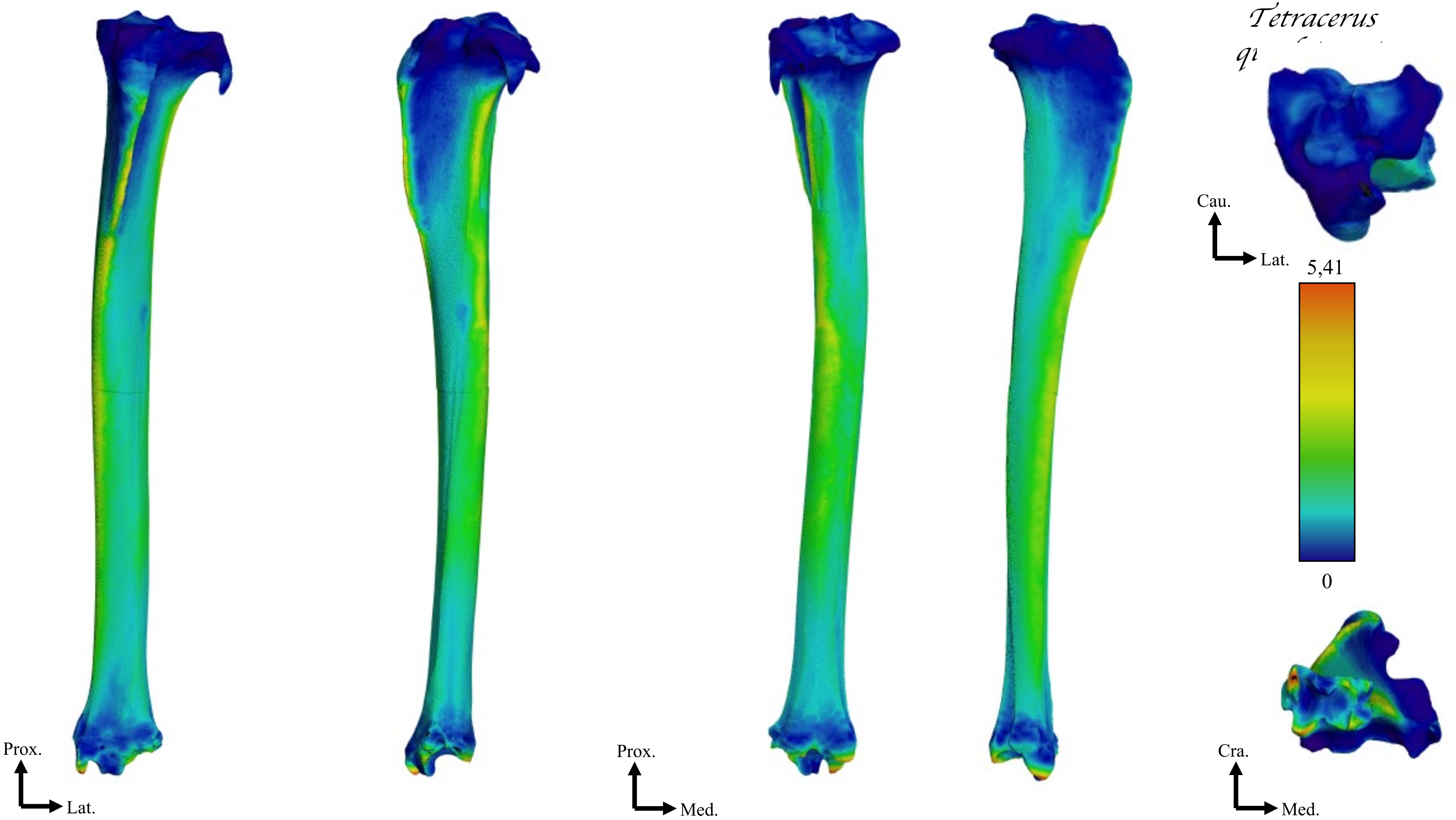
Tetracerus
Cau.
Lat.
5,41
0
Prox.
Lat.
Prox.
Med.
Cra.
Med.

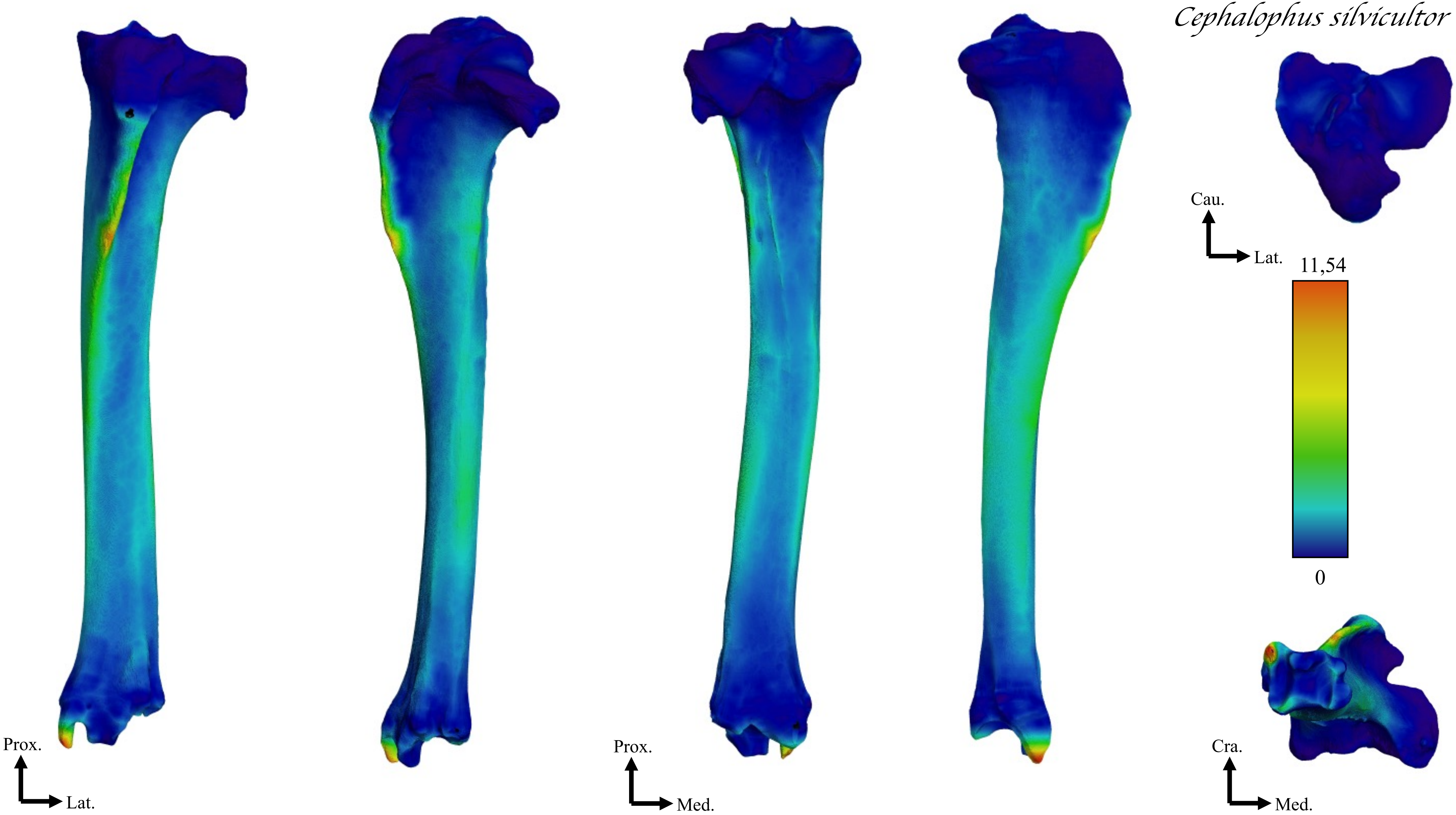

Cephalophus silvicultor
Cau.
Lat.
11,54
0
Prox.
Lat.
Prox.
Med.
Cra.
Med.

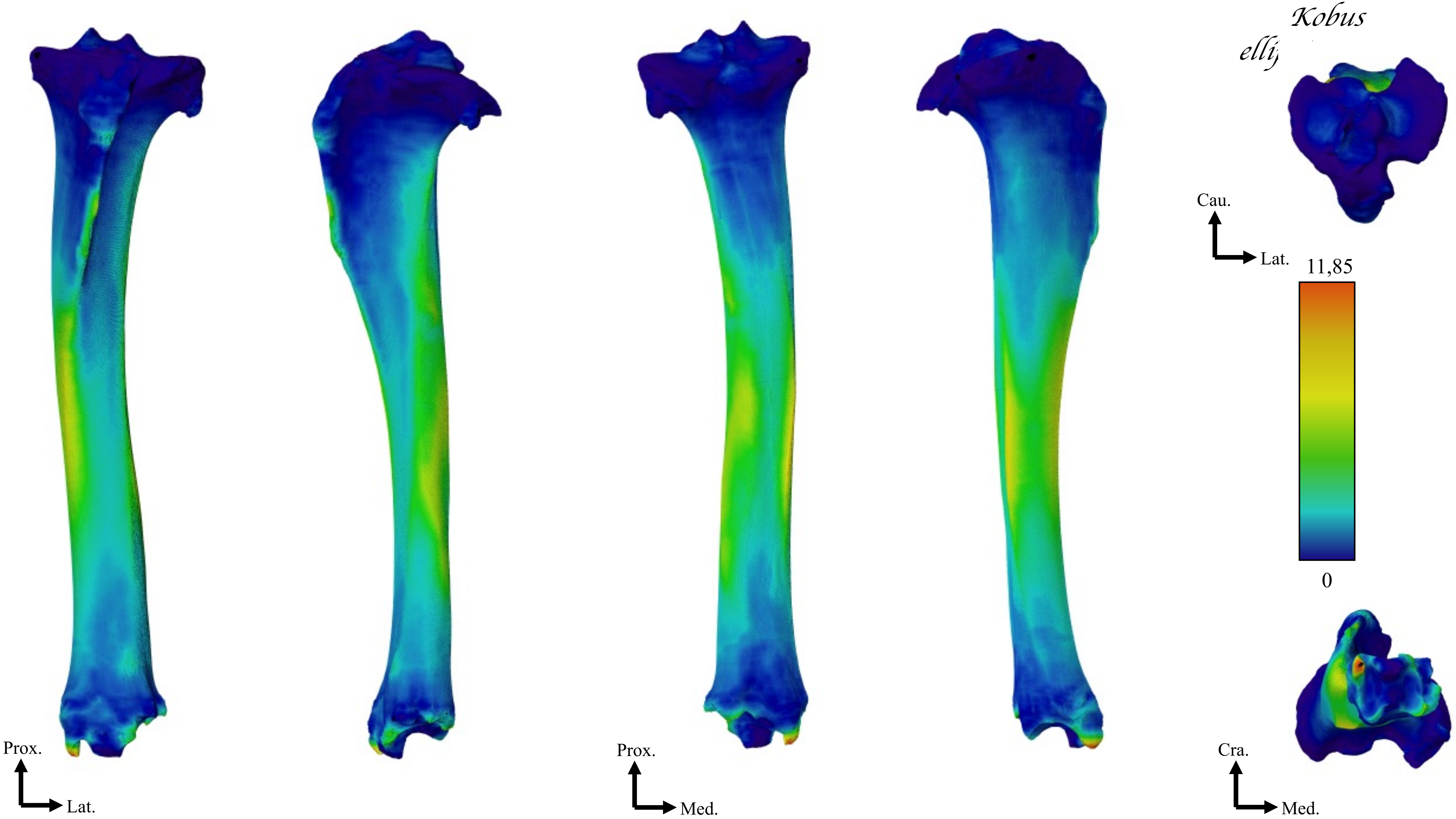

Kobus
ellij
Cau.
Lat.
11,85
0
Prox.
Lat.
Prox.
Med.
Cra.
Med.

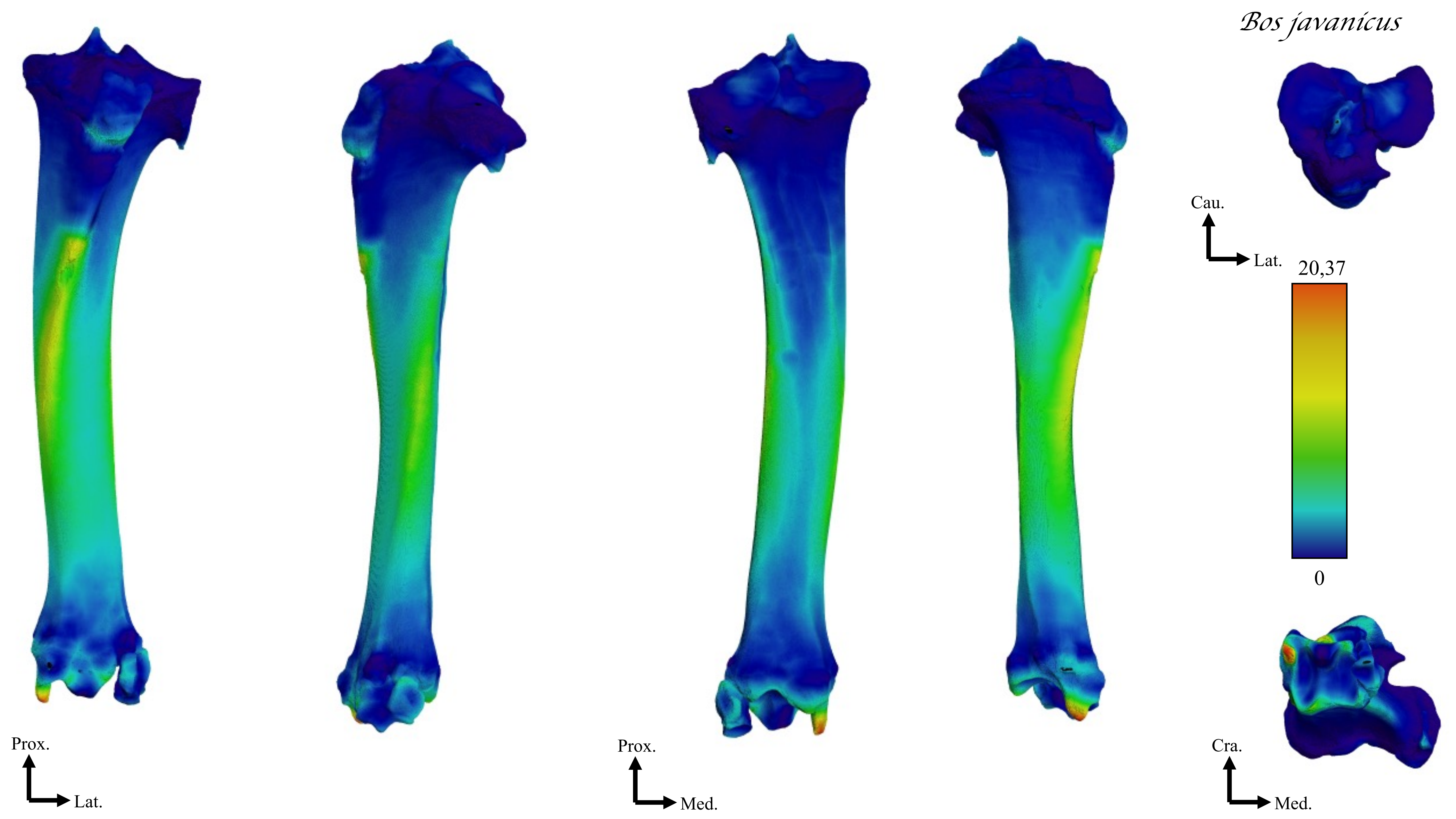

Bos javanicus
Cau.
Lat.
20,37
0
Prox.
Lat.
Prox.
Med.
Cra.
Med.

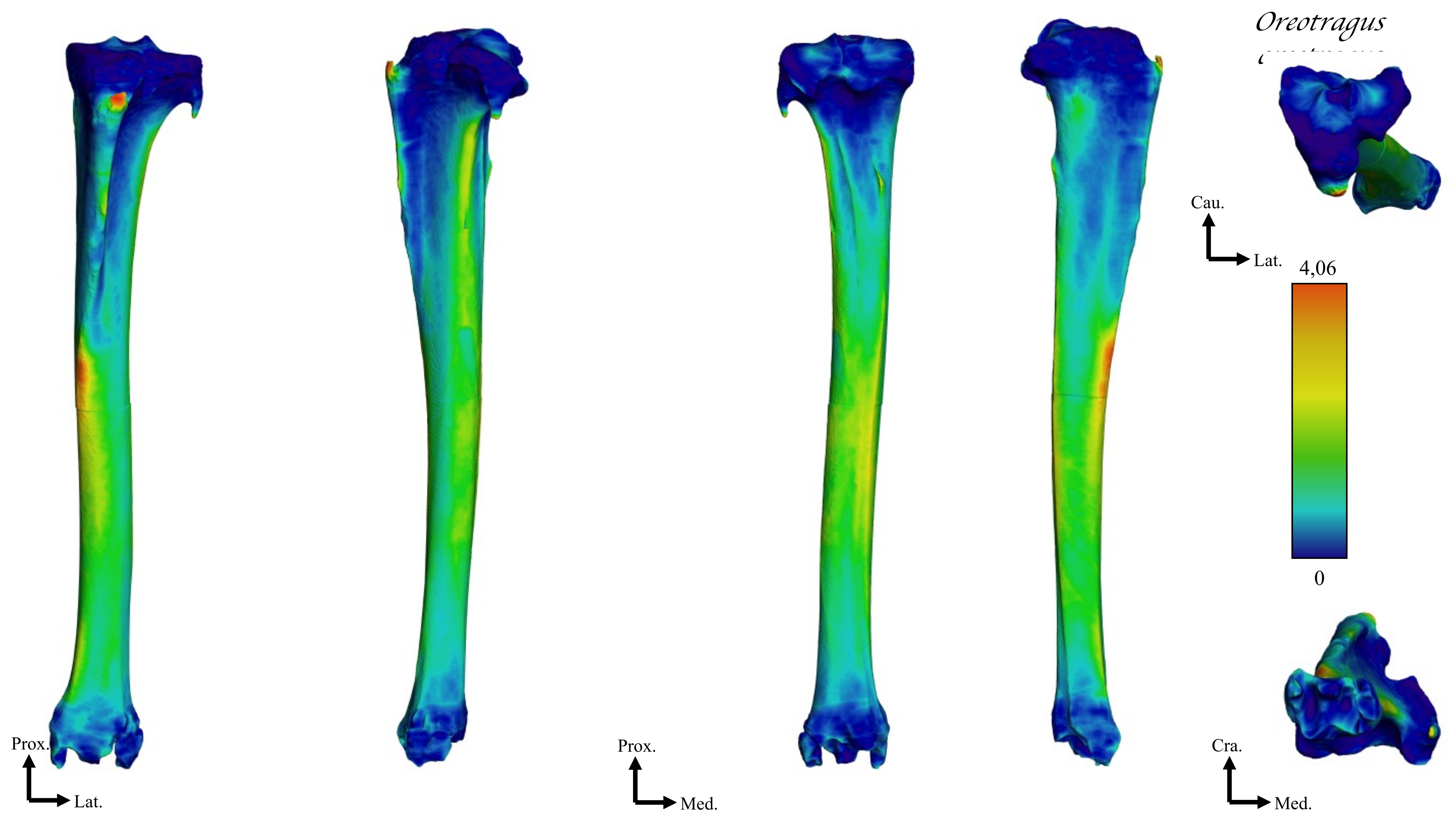

Oreotragus
Cau.
Lat.
4,06
0
Prox.
Lat.
Prox.
Med.
Cra.
Med.

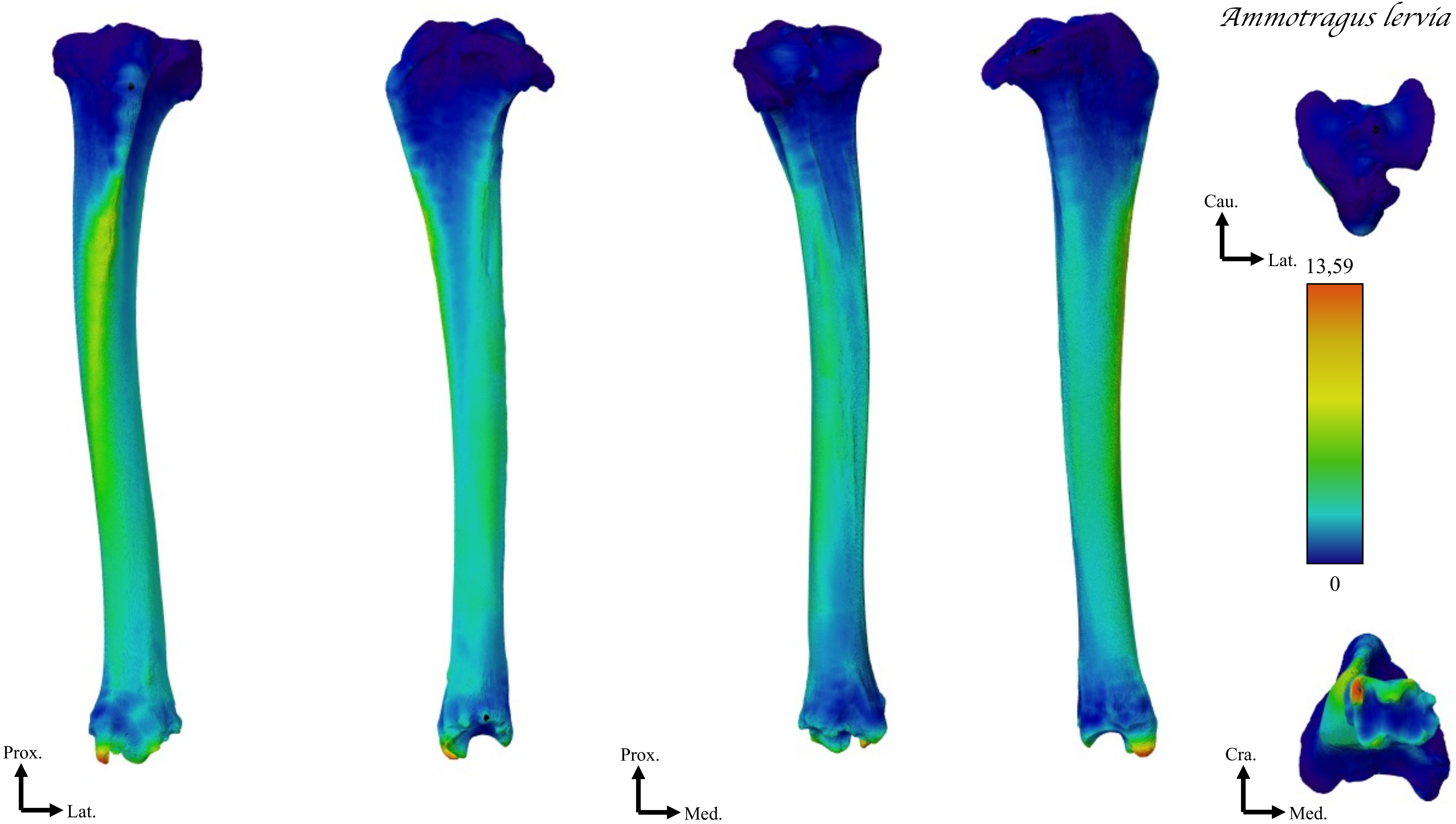

Ammotragus lervia
Cau.
Lat.
13,59
0
Prox.
Lat.
Prox.
Med.
Cra.
Med.

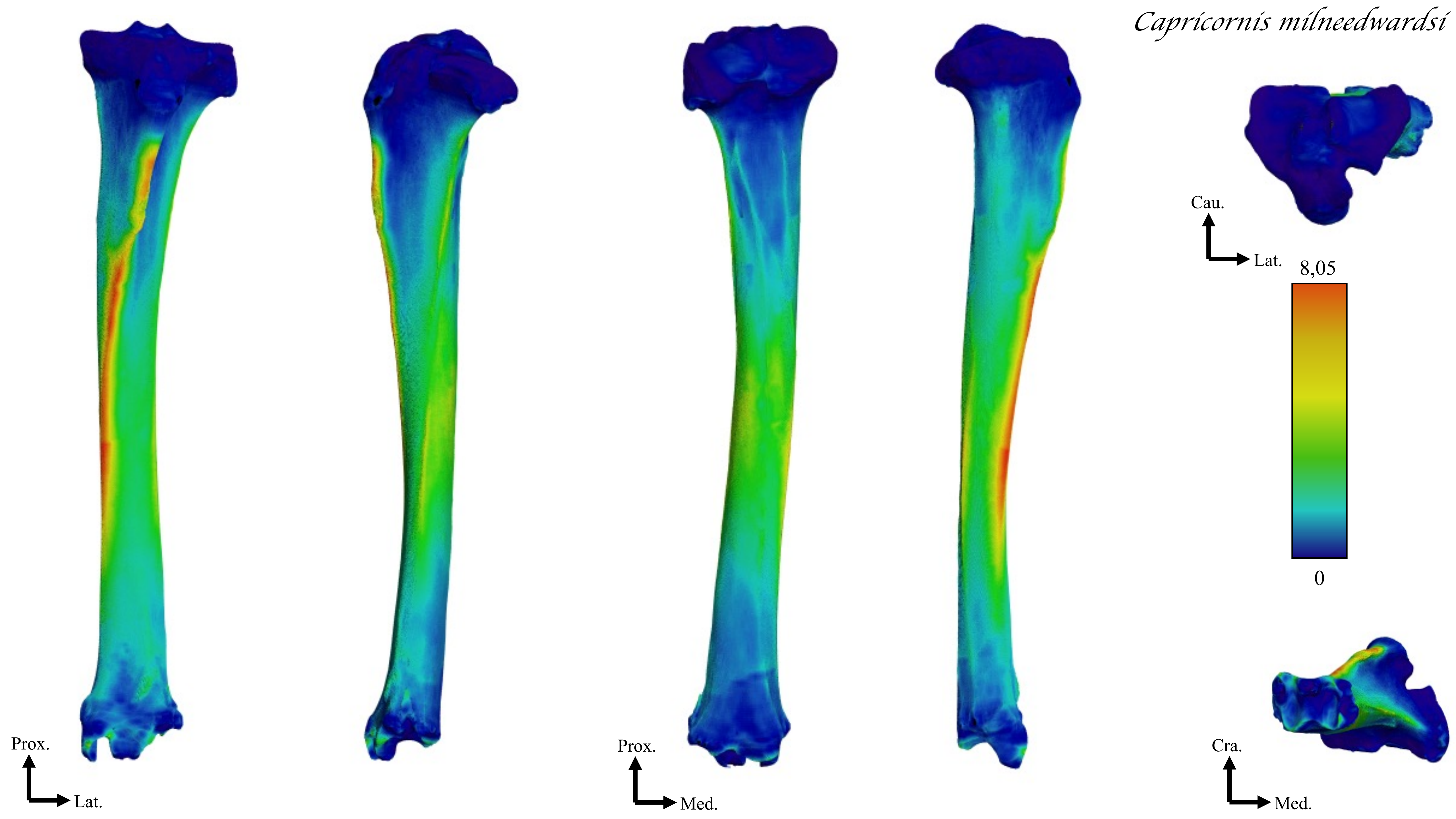

Capricornis milneedwardsi
Prox.
Lat.
Prox.
Med.
Cau.
Lat.
8,05
0
Cra.
Med.
